# Probing the Therapeutic Potential of Marine Phyla by SPE Extraction

**DOI:** 10.3390/md19110640

**Published:** 2021-11-16

**Authors:** Alejandro Moreiras-Figueruelo, Genoveffa Nuzzo, Christian Galasso, Clementina Sansone, Fabio Crocetta, Valerio Mazzella, Carmela Gallo, Giusi Barra, Angela Sardo, Antonella Iuliano, Emiliano Manzo, Giuliana d’Ippolito, Marte Albrigtsen, Jeanette H. Andersen, Adrianna Ianora, Angelo Fontana

**Affiliations:** 1 Bio-Organic Chemistry Unit, Institute of Biomolecular Chemistry, Consiglio Nazionale delle Ricerche, Via Campi Flegrei 34, Pozzuoli, 80078 Naples, Italy; a.moreiras@icb.cnr.it (A.M.-F.); carmen.gallo@icb.cnr.it (C.G.); giusi_barra@hotmail.it (G.B.); emanzo@icb.cnr.it (E.M.); gdippolito@icb.cnr.it (G.d.); 2 Consorzio Italbiotec, Via Gaudenzio Fantoli, 16/15, 20138 Milano, Italy; 3 Calabrian Researches Centre and Marine Advanced Infrastructures, Department of Marine Biotechnology, National Institute of Biology, Ecology and Marine Biotechnology, Stazione Zoologica Anton Dohrn, C.da Torre Spaccata, 87071 Amendolara, Italy; christian.galasso@szn.it; 4 Departmentt of Marine Biotechnology, National Institute of Biology, Ecology and Marine Biotechnology, Stazione Zoologica Anton Dohrn, 80121 Naples, Italy; clementina.sansone@szn.it (C.S.); angela.sardo@szn.it (A.S.); adrianna.ianora@szn.it (A.I.); 5 Department of Integrative Marine Ecology, National Institute of Biology, Ecology and Marine Biotechnology, Stazione Zoologica Anton Dohrn, 80121 Naples, Italy; fabio.crocetta@szn.it (F.C.); valerio.mazzella@szn.it (V.M.); 6 Department of Mathematics, Computer Science and Economics, Campus Macchia Romana, University of Basilicata, Viale dell’Ateneo Lucano 10, 85100 Potenza, Italy; antonella.iuliano@unibas.it; 7 The Norwegian College of Fishery Science, Marbio, UiT—The Arctic University of Norway, 9019 Tromsø, Norway; marte.albrigtsen@uit.no (M.A.); jeanette.h.andersen@uit.no (J.H.A.); 8 Laboratory of Bio-Organic Chemistry and Chemical Biology, Department of Biology, University of Naples “Federico II”, Via Cupa Nuova Cinthia 21, 80126 Napoli, Italy

**Keywords:** marine natural products, small molecules, drug discovery platform, pre-fractionation method, active metabolites, cytotoxic, antimicrobial and antidiabetic activity

## Abstract

The marine environment is potentially a prolific source of small molecules with significant biological activities. In recent years, the development of new chromatographic phases and the progress in cell and molecular techniques have facilitated the search for marine natural products (MNPs) as novel pharmacophores and enhanced the success rate in the selection of new potential drug candidates. However, most of this exploration has so far been driven by anticancer research and has been limited to a reduced number of taxonomic groups. In this article, we report a test study on the screening potential of an in-house library of natural small molecules composed of 285 samples derived from 57 marine organisms that were chosen from among the major eukaryotic phyla so far represented in studies on bioactive MNPs. Both the extracts and SPE fractions of these organisms were simultaneously submitted to three different bioassays—two phenotypic and one enzymatic—for cytotoxic, antidiabetic, and antibacterial activity. On the whole, the screening of the MNP library selected 11 potential hits, but the distribution of the biological results showed that SPE fractionation increased the positive score regardless of the taxonomic group. In many cases, activity could be detected only in the enriched fractions after the elimination of the bulky effect due to salts. On a statistical basis, sponges and molluscs were confirmed to be the most significant source of cytotoxic and antimicrobial products, but other phyla were found to be effective with the other therapeutic targets.

## 1. Introduction

Natural products and their derivatives have long been an inspiration in drug discovery. Among these, marine natural products (MNPs) are generally characterized by high chemical diversity and unusual pharmacological properties that make them suitable as lead structures in the development of new drugs. A significant number of pharmacological activities from MNPs have so far been reported in various fields, including analgesic, anti-inflammatory, antibiotic, antibacterial, antifungal, antiviral, and antiparasitic [1]. In recent years, the techniques used to identify and characterize the new products have been improved and a large number of new databases have been implemented, with the aim of classifying and simplifying their selection [2]. In addition, increasingly more advanced tests have been developed in order to improve both the accuracy of preclinical selection and the clinical prediction required for subsequent translational developments [3]. As a result of these efforts, a growing number of compounds from marine sources are in clinical trials, and there is a general consensus on the increasing impact of MNPs on the pharmaceutical industry in the coming years [4,5]. However, there are still several challenges associated with the experimental approaches in marine drug discovery, including supply limitation, structural complexity, rediscovery rates, moderate structure stability, and the masking of bioactive compounds. In particular, as preliminary screenings are carried out by using crude or semi-fractionated mixtures, a large amount of inorganic salts and neutral lipids can interfere with the biological response and can hide the presence of the active products. To bypass these problems, different fractionation methods have been developed to treat the extract before the biological assays [6,7].

In our laboratory, we set up and validated a Solid Phase Extraction (SPE) procedure based on a hydrophobic resin [8]. Compared to crude extracts, SPE samples, although still made up of complex mixtures, showed an improved screening performance (with a higher confidence in observed hit rates), enhanced biological activity due to the major concentration of active components present as only minor metabolites, as well as simplified processes for the dereplication and isolation of the bioactive molecules. In the present study, we aimed to test this approach in a totally automatized screening platform by using, as proof of concept, an in-house library of small molecules composed of the extracts from 57 marine macro- and micro-organisms chosen from among the major eukaryotic phyla so far considered for bioprospection. The samples were screened for antitumor, antibiotic, and antidiabetic activity by two complementary approaches based on a phenotypic (classical pharmacology) and a target-based assay (reverse pharmacology) [9]. The first approach consisted of viability tests for cytotoxic activity and bacterial density measurement for bactericide evaluation. The second approach was a type 2 diabetes enzymatic assay, used for the identification of molecules that are able to inhibit Protein Tyrosine Phosphatase 1B (PTP-1B), which arrests the signalling of the insulin receptor in vivo and is essential for the physiological control of glycaemia increase in insulin-responsive tissues [10].

## 2. Results and Discussion

### 2.1. Collection of Organisms

**Figure 1:**
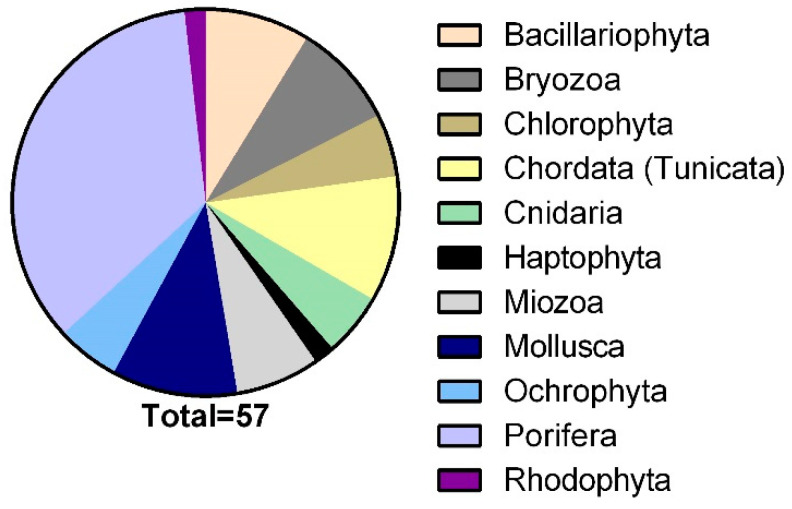
Phyla distribution of marine organisms processed by the screening platform.

Fifty-seven different marine organisms, selected from among benthic invertebrates and microorganisms, were collected in order to obtain a biologically diversified source of natural extracts (Figure 1). In addition to their availability, these specimens offered a wide evolutionary heterogeneity that allowed for a good diversification of secondary metabolites. The organism collection, based on their origin, can be classified into two main groups: cultured microorganisms (in-house and outsourced) and collected species. This latter group is formed by organisms coming from the Mediterranean Sea and Antarctica.

#### 2.1.1. Cultured Microorganisms

In-house cultured organisms included protists from the phyla Bacillariophyta (diatoms), Rhodophyta (red algae), Chlorophyta (green algae), Haptophyta, and Miozoa (dinoflagellates). The dinoflagellates *Heterocapsa* sp. and *Scrippsiella* sp. were isolated from seawater samples, while the epiphytes *Amphidinium massartii* (Biecheler) and *Amphidinium carterae* (Hulburt) were obtained from their algal hosts. Diatoms *Thalassiosira rotula* (Meunier) and *Phaeodactylum tricornutum* (Bohlin) were isolated from seawater samples, while *Thalassiosira pseudonana* (Hasle and Heimdal), *Skeletonema marinoi* (Sarno and Zingone), and *Cyclotella cryptica* (Reimann, J.C.Lewin, and Guillard) were purchased from the Culture Collection of Marine Phytoplankton (CCMP). Four species of microalgae (*Tretraselmis suecica*, *Tetraselmis* sp., *Nannochloropsis* sp., and *Isochrysis galbana*) were obtained as lyophilized samples from external producers. The source of each microorganism and the assigned codes are reported in Table 1. Unless differently stated, these species were cultured up to the stationary phase and centrifuged according to our previously reported procedures [8]. The wet pellets were immediately frozen and stored until extraction.

#### 2.1.2. Benthic Invertebrates and Algae

Invertebrates comprise the group richest in biodiversity among animals and include about 30 phyla. The major phyla include Porifera, Cnidaria, Annelida, Bryozoa, Mollusca, Arthropoda, and Echinodermata [11]. Some of these phyla are characterized by few species, while others include more than 85% of all described animal species and consist of over a million species [11]. In 2012, Leal et al. [12] reported the characterized MNPs and classified them according to phyla based on their invertebrate source. They found that approximately 80% of these were from the phyla Porifera (47.1%) and Cnidaria (32.9%).

As shown in Figure 1, poriferans, molluscs, and tunicates account for more than fifty percent of the organisms that were selected in the present study. Such a distribution reflects the number of studies that have so far been carried out on MNPs [13]. In particular, poriferans are one of the main sources of bioactive MNPs, providing a good number of molecules already available as drugs or candidates in clinical trials [14], along with algae, molluscs, and corals as well as microorganisms, which have recently and significantly contributed to the discovery of novel MNPs [14,15]. Eighteen of the forty-four organisms were collected in the Antarctic Ocean (Ross Sea), whereas the remaining ones come from the Mediterranean Sea, mostly collected in the Gulf of Naples (Italy). For most of them, the taxonomical identification was available (Table 2), while for the taxonomically undefined samples, voucher specimens in ethanol are deposited at the CNR-Institute of Biomolecular Chemistry.

### 2.2. Library of Marine Natural Products Extracts

Raw extracts were obtained by an exhaustive extraction of each marine organism with MeOH. After the evaporation of the solvent by rotavapor, the samples were transferred using a mixture of MeOH/Dichloromethane (DCM) 2:1 (*v*/*v*) to partially desalt the extracts by salt precipitation. After the removal of the solvent under nitrogen, the dry extracts were stored under argon at −80 °C until use.

In agreement with [8], SPE fractionation was based on the use of the hydrophobic solid phase of polystyrene–divinylbenzene. Each marine extract (50 mg for benthic species and 25 mg for microalgae) was loaded as a water suspension, and fractionation was achieved by a stepwise solvent gradient elution. Briefly, the first mobile phase used pure water (fraction A) for an efficient desalting. Organic compounds were then eluted in the four next fractions with MeOH:H_2_O 1:1 (*v*/*v*), Acetonitrile (ACN):H_2_O 7:3 (*v*/*v*), ACN, and DCM:MeOH 9:1 (*v*/*v*) (fractions B–E). This procedure gave a reproducible and accurate fractionation of the organic chemical classes, from polar to apolar products. The percentage of each fraction in the whole extract for phylum is reported in Table 3. The distribution of the metabolites in the enriched fractions for each SPE was monitored by thin layer chromatography (TLC) and ^1^H NMR. Overall, the pre-fractionation platform generated an MNP library composed of 228 enriched fractions and 57 raw extracts to be tested.

### 2.3. Bioassays

For the biological screening, three different bioassays were implemented, including both cell-based assays and a biochemical assay. The test panel relied on the three different screening platforms, fast to perform with very low amount of material: two phenotypic assays, to test cytotoxicity and antimicrobial activity, and one targeted to evaluate antidiabetic activity.

#### 2.3.1. Cytotoxic and Antimicrobial Bioassays

The extracts and fractions were tested on two human cancer cell lines, A549 (adenocarcinoma human alveolar basal epithelial cells) and A2780 (human ovarian cancer cell line), together with PNT2 (normal prostate epithelium immortalized with SV40), which was used as control. The choice of these cell lines was associated with the frequency of these tumors, their metastatic potential, their easy manipulation, and their high proliferation rate compared with other available cell lines.

**Figure 2:**
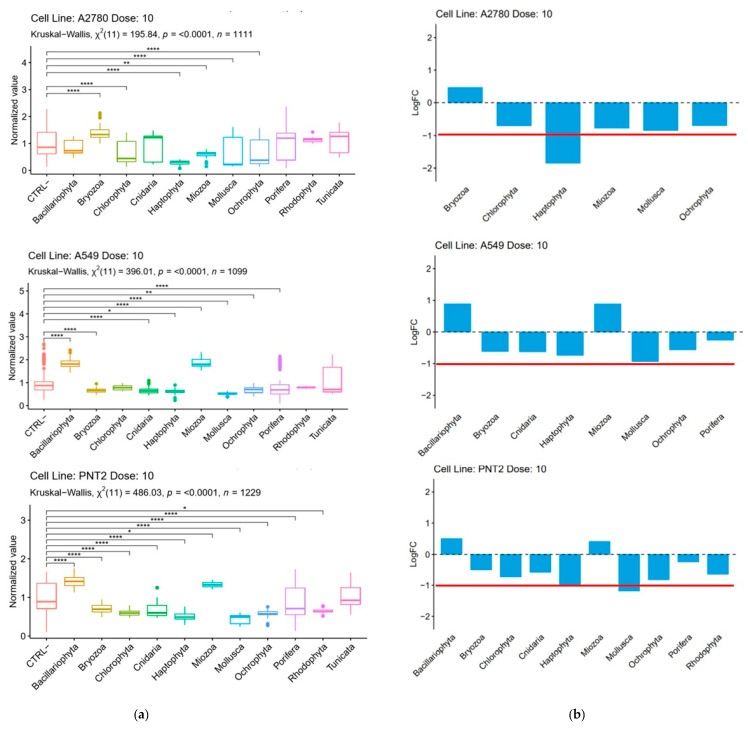
Distribution of positive hits among the taxonomic groups represented in the library. (**a**) Kruskal–Wallis test (non-parametric test) performed for each cell line (A2780, A549, PNT2) at a dose of 10 µg/mL. Post hoc tests were applied to identify which phylum groups were statistically different (significant codes: 0.0001 ‘****’, 0.01 ‘**’, 0.05 ‘*’). (**b**) Positive (negative) log2 fold-change for significant comparisons (i.e., *p*-value < 0.05) between phylum groups and untreated cells (CTRL-) are shown. Red line indicates treatment with 50% mortality.

Cytotoxicity was measured after 48 h of incubation with the MTT assay [16] (File S1 of the Supporting Material). The results were expressed as the percentage of cell vitality after treatment at three different concentrations (1, 10, and 100 μg/mL) with extracts and fractions; values were calculated as the ratio between the mean absorbance of each treatment and the mean absorbance of control (untreated cells). The effect on the survival of control cells at multiple extract concentrations allowed for the prioritization of hit extracts before further studies. Samples resulting as cytotoxic only at the highest concentration (100 μg/mL) were considered not of interest. A statistical analysis of phyla with a non-parametric Kruskal–Wallis test revealed that the most significant results were observed at 10 μg/mL (Figure S1). At this concentration, treatment with Haptophyta, Bryozoa, Miozoa, Mollusca, and Ochorophyta showed a significant difference versus control (*p*-value < 0.05) in all the cell lines (Figure 2), although for Bryozoa and Miozoa, these differences were ascribable to the increase of cell proliferation instead of cytotoxicity. Considering only the effects related to toxicity, Cnidaria and Porifera induced a significant reduction of growth on A549 and PTN2, as well as Chlorophyta on A2789 and PTN2.

**Figure 3:**
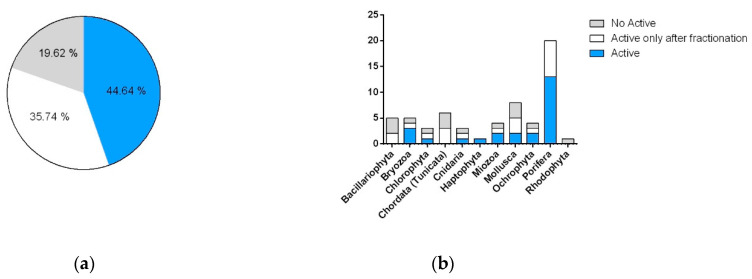
Cytotoxic activity of the taxonomic groups represented in the library. (**a**) Total percentages of positive hits against cancer cell lines A549 and A2780. (**b**) Number of cytotoxic species grouped by phyla. Blue indicates organisms with activity in raw extracts and SPE fractions. White indicates organisms with activity only in SPE fractions. Grey indicates samples that are not active.

Exclusively considering samples that were able to reduce cell vitality to below 80% at 1 or 10 μg/mL in the A549 and A2780 lines, we compared the number of organisms active as raw extracts with organisms active only after fractionation. Results showed an increase in positive hits by more than 35% after SPE fractions (Figure 3). Analysis of these data underlined that Bacillariophyta, Mollusca, and Chordata (Tunicata) were the phyla whose activity took most advantage of the fractionation and showed the highest increase in positive hits in comparison to raw extracts. Only in one case, namely, the bryozoan *Amathia verticillata* CBC30A, was the activity detected solely in the extract. Parallel tests on the three cell lines allowed for the identification of samples that differentially affected the two cancer models, as well as the detection of non-specific cytotoxic compounds and false positives (data not shown).

**Figure 4:**
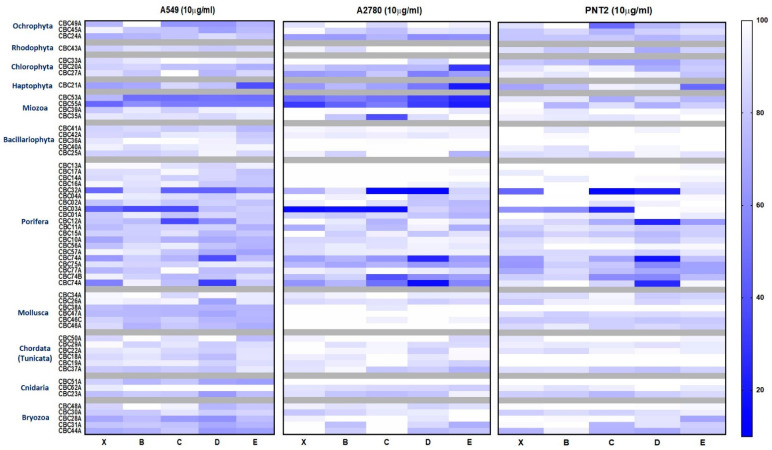
Heatmap showing the percentage of MTT viability in the three cell lines after treatment with the raw extracts and enriched fractions at 10 μg/mL. X = raw extracts; B–E = SPE fractions.

Figure 4 summarizes the activity on the three cell lines after treatment with the raw extracts (X) and related SPE fractions (B, C, D, E) at 10 μg/mL. Among the protist phyla, the two dinoflagellates, *Heterocapsa* sp. (CBC53A) and *Scrippsiella* sp. (CBC55A), were cytotoxic, with an enrichment of the activity after fractionation and a mild effect on the non-tumorigenic cell line PNT2. Moreover, fraction E of the diatom *Tetraselmis* sp. (CBC20A) displayed a specific cytotoxicity on the A2780 cell line, showing a strong inhibition of cell proliferation (29.8% of cell viability) at 1 µg/mL (Figure S3). Similar results were obtained with fraction C of *Amphidinium carterae* (CBC35A), which affected A2780 cell viability (37.8%) at 1 µg/mL of the tested concentration (Figure S3).

On the whole, in line with the literature, the most active extracts were associated with the phylum Porifera (Figure 4). Sponge fractions showed little specificity since the most cytotoxic samples were indifferently active on both the two tumor cell lines and controls. These samples included species already known for the presence of toxic metabolites such as *Spirastrella cunctatrix* CBC32A [17,18], *Crambe* CBC03A [19], and the Antartic sponge *Dendrilla* sp. CBC74A [20,21].

Between the two cancer cell lines, pulmonary adenocarcinoma cells (A549) appeared to be more resistant to treatment with samples from molluscs, cnidarians, and bryozoans. Within molluscs, fraction D of *Philina quadripartita* (CBC26A) reduced viability to 66.2% at 10 µg/mL, with a clear dose-dependent response effect. Analogously, fractions D and E of the cnidarian extract CBC51A and fractions C and D of *Bugula neritina* (CBC28A) reduced the cell survival of A549 by a percentage (around 65% at 10 µg/mL) strongly dependent on the concentration of the active component.

#### 2.3.2. Antimicrobial Bioassay

The second biological investigation of our MNP library included tests for potential antimicrobial activity. The large use of antibiotics and the concomitant development of biological resistance have rendered the identification of novel antimicrobial compounds of vital importance to the prevention of the spread of infections due to resistant bacteria [22]. For antibacterial screening, we selected a small panel of diverse pathogens, including three Gram+ (*Staphylococcus. Aureus*; *Enterococcus faecalis,* and *Streptococcus agalactiae*) and two Gram− (*Escherichia coli* and *Pseudomonas aeruginosa*) strains that have been routinely used as quality controls in clinical laboratories (EUCAST E. Dis 5.1. March 2003). The bacterial strains were grown in multi-well plates and treated with 50 µg/mL of either extract or fractions B-E. The effects of this treatment were measured after 20 h, and the results were expressed as percentage of growth control (untreated bacteria) (File S2 of Supplementary Material).

**Figure 5:**
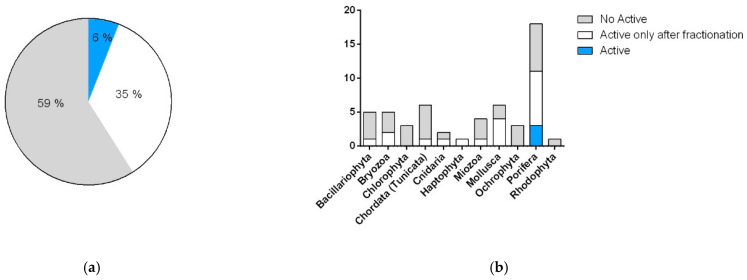
Antibacterial activity of the taxonomic groups represented in the library. (**a**) Total percentages of positive hits against Gram+ and Gram− strains. (**b**) Number of species with antimicrobial activity, grouped by phyla. Blue indicates organisms with activity in raw extracts and SPE fractions. White indicates organisms with activity only in SPE fractions. Grey indicates samples that are not active.

In comparison to the cytotoxicity reported above, most of the samples (59%) did not show any antimicrobial potential. However, the tests showed a clear increase in activity after fractionation, with a positive response in 6% of the raw extracts versus 35% of positive hits in the enriched SPE fractions (Figure 5). With the exception of dinoflagellates and diatoms, microalgae did not seem to produce antimicrobial products. On the contrary, the best positive score was observed in molluscs, with almost 80% of the samples showing antimicrobial activity, and sponges.

**Figure 6:**
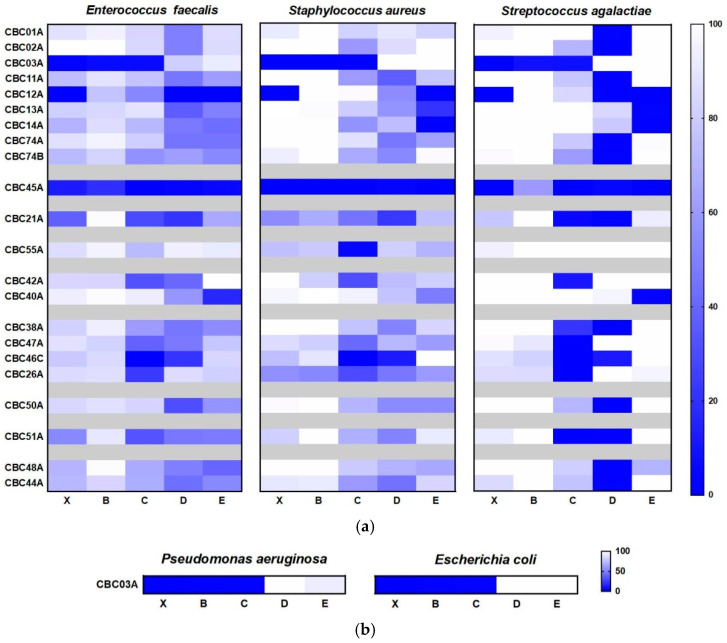
Heatmap showing the percentage of inhibition of Gram+ (**a**) and Gram− (**b**) bacterial strains after treatment with the active samples of the MNP library at the concentration of 50 mg/mL. X = raw extracts; B–E = SPE fractions.

As reported in Figure 6, most of the active samples exhibited activity only against Gram+ strains. In general, *Streptococcus agalactiae* was much more sensitive than *Staphylococcus aureus* and *Enterococcus faecalis* in the tests with the MNPs. Regardless of the taxonomic groups, most of the activities were related to the SPE fractions C and D, which are particularly enriched in small molecules with little polarity such as terpenes, sterols, and polyketides. In agreement with the literature [23,24], the extract and the SPE fractions B and C of the sponge *Crambe crambe* (CBC03A) were active against both Gram− and Gram+ strains. This sponge showed almost 100% inhibition of bacterial growth and was also the only species with promising effectiveness in targeting Gram− pathogens.

Crambescedins are the main metabolites of *C. Crambe* [19]. These compounds are good candidates for the antimicrobial activity of this sponge. Nevertheless, the identification of the single bioactive moelcule was aside the aim of the present study, and further investigations are necessary to confirm the chemical agents responsible for the observed activity.

#### 2.3.3. Antidiabetic Bioassay

In addition to the two phenotypic assays, we tested the potential of our chemical library of MNPs to target protein tyrosine phosphatase 1B (PTP-1B), a key signal-transduction regulator involved in the etiology of diabetes mellitus [25]. PTP-1B functions as a negative regulator of insulin and as a drug target in order to ameliorate resistance to the hormone [26]. For the screening of the MNP library, we used an enzymatic assay for the inhibition of the recombinant human PTP-1B protein together with the T Cell-PTP counter screen assay to check for enzymatic specificity [27]. We also tested 50 μg/mL extracts or fractions of the MNP library, and inhibition was calculated by comparing measurements with controls (no treatment). Activity threshold was set to less than 30% of the enzymatic activity, and fractions that were active only against the PTP-1B were selected.

**Figure 7:**
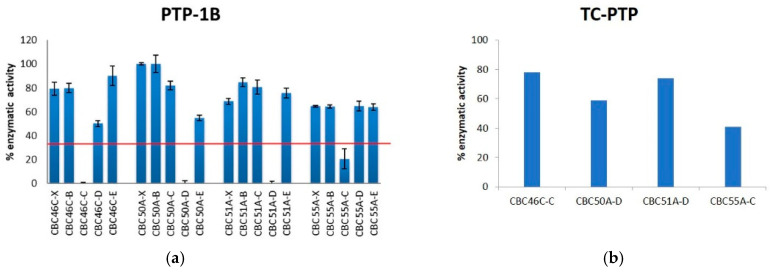
Enzymatic tests for the selection of antidiabetic candidates. (**a**) PTP 1B inhibition by selected samples from the MNP library. Activity threshold was below 30% (red line). (**b**) Inhibition of T Cell PTP.

Only four organisms, including the mollusc *Gastropteron rubrum* (CBC46C), the chordate CBC50A, the cnidarian CBC51A, and the dinoflagellate *Scrippsiella* sp. (CBC55A), showed noteworthy properties (Supporting Material, File S3). Significantly, PTP-1B activity was below 30% only in enriched samples after SPE fractionation (Figure 7), thus further confirming the potential of the pre-fractionation platform. Similar to the antimicrobial activity, SPE fractions C and D were those containing the active compounds. Fractions of *Gastropteron rubrum* (CBC46C-C) and the cnidarian CBC51A (CBC51A-D) were specific for PTP-1B, thus resulting as the most promising candidates for further development. In contrast to the cytotoxic and antimicrobial tests, sponges did not give positive hits.

## 3. Materials and Methods

### 3.1. General

All the chemicals and solvents (Sigma-Aldrich, Milan, Italy) were of analytical reagent grade and were used without any further purification. For the centrifugation of in-house cultured organisms, we used an Allegra X-12R centrifuge (Beckman Coulter, Milan, Italy).

The solid phase extraction was carried out using both prepacked and non-polystyrene–divinylbenzene columns (CHROMABOND^®^ HR-X, Macherey-Nagel, Düren, Germany). Automated fractionations were carried out on the GX-271 ASPEC Gilson apparatus equipped with TRILUTION^®^ LH Software (version No. 3.0, Gilson, Middleton, WI, USA). Silica gel chromatography was performed using precoated Merck F254 plates.

^1^H NMR spectra were recorded on a Bruker DRX 600 spectrometer equipped with an inverse TCI CryoProbe or 400 equipped with a CryoProbe Prodigy. Extracts were dissolved in 700 µL CDCl_3_/CD_3_OD 1:1 (*v/v*) and transferred to the 5 mm NMR tube. Chemical shift was referred to CHD_2_OD signal at δ 3.34.

### 3.2. Marine Organism Sources

#### 3.2.1. Cultured Algae

Nine of our microalgae were cultured in-house (see Table 1), in 10 or 2 L polycarbonate carboys, in a room with controlled temperature (20–22 °C) via sterile air injectors, and artificial illumination using cool fluorescent tubes.

Miozoa were isolated directly from seawater by the capillary pipette method. The free-living species *Heterocapsa* sp. and *Scrippsiella* sp. were directly picked up from seawater samples, while the epiphytes *Amphidinium massartii* and *Amphidinium carterae* first required detachment from the macroalgae *Asparagopsis taxiformis* (Delile) Trevisan and *Dictyota dichotoma* (Hudson) J.V. Lamouroux samples, respectively. To do this, the host seaweeds were vigorously shaken in plastic bags containing filtered seawater, which was then used to apply the conventional procedure of single-cell isolation. All species were cultured in K medium [28] using air bubbling (ca. 3 L/min) provided by filter venting devices. Dinoflagellates were grown under an artificial light intensity of 100 μmol photons m^−2^ s^−1^ and a 14:10 light:dark photoperiod. The culture lasted for 12–15 days until the stationary phase, which was then harvested by centrifugation (2300× *g*, 12 °C, 10 min per round) and stored frozen until extraction.

Bacillariophyta (diatoms) were cultured in f/2 medium [29] at higher irradiances (200 μmol photons m^−2^ s^−1^); other conditions were similar to those already mentioned. *Thalassiosira rotula* and *Phaeodactylum tricornutum* were obtained directly from seawater samples and isolated via the capillary pipette method. The remaining three diatoms, *Thalassiosira pseudonana*, *Skeletonema marinoi,* and *Cyclotella cryptica,* were purchased from the National Center for Marine Algae and Microbiotics (NCMA).

*Tetraselmis suecica* was bought as dry biomass from Neoalgae Microseaweeds Products, Gijón, Spain, while *Tetraselmis* sp., *Nannochloropsis* sp., and *Isochrysis galbana* were purchased from Phytobloom, Necton.

#### 3.2.2. Benthonic Invertebrates and Algae Sampled

Benthic organisms were collected directly from the sea during various sampling campaigns (see Table 2). They were frozen as soon as possible and stored at −20 °C until they were processed.

### 3.3. Methanol Extraction of the Organisms

All the samples were weighted prior to proceeding with solvent extraction. Porifera were lyophilized and then weighted as dry biomass.

Extraction proceeded by covering each organism with methanol and grounding it with a mortar in such way that most of the volume was exposed to the solvent; it was then extracted exhaustively using mechanical means and sonication. This procedure was repeated at least two times, and the recovered solvent was mixed. During the extraction of the samples, an ice bath was used to keep the temperature of the extract low and prevent degradation. For the extractions of microalgal pellets, a volume of 5 mL of methanol per gram of sample was used, combined with sonication to disrupt the cells.

The extracts were filtered with a rinsed filter paper, and dried in a rotatory evaporator using a maximum temperature of 27 °C. This full crude extract was weighted and aliquoted when transferred to vials, using methanol–dichloromethane 2:1 (*v/v*) as solvent. Finally, this crude extract was dried under a nitrogen flow and kept dry at −80 °C.

### 3.4. Solid Phase Extraction

An aliquot (about 50 mg, and 25 mg for more lipophilic samples) of raw extracts was suspended in 1 mL of MilliQ water, using sonication as needed, and then incorporated into the columns of the automatic SPE device.

The instrument was first programmed to activate the column by eluting with 5 mL methanol, followed by equilibration with 10 mL of MilliQ water. The next step consisted in loading the 1 mL extract suspension to the column. Then, another 2 mL of MilliQ water was added to the extract vial to be pipetted in order to resuspend as much remaining extract as possible. The leftover was then added to the column. Then, another 6 mL of MilliQ water was added directly to the column to complete the elution of fraction A. Afterwards, 9 mL of each eluent was used stepwise to obtain the corresponding fractions B (methanol-water 1:1), C (acetonitrile-water 7:3), D (pure acetonitrile), and E (dichloromethane-methanol 9:1). The eluted fractions were transferred to round bottom flasks and dried in a rotatory evaporator using a maximum temperature of 27 °C. The content from the round bottom flasks was then transferred to vials, dried under nitrogen flow, weighted, and conserved at −80 °C.

Chemical fractions to be tested were prepared by dissolving raw extract X and SPE fractions B, C in methanol, whereas the more lipophilic SPE fractions D and E were diluted in methanol–dichloromethane 1:1. The solvent was removed under vacuum, and the samples were immediately frozen and kept at −20 °C until performance of the assay.

### 3.5. Cytotoxicity Assays

Cytotoxicity experiments were performed on three different cell lines: human cancer cell lines, A549 (adenocarcinoma human alveolar basal epithelial cells), and A2780 (human ovarian cancer cell line), together with PNT2 (normal prostate epithelium immortalized with SV40), all obtained from the American Type Culture Collection (ATCC). A549 cells were cultured in DMEM F12 (Dulbecco’s modified Eagle’s medium) supplemented with 10% fetal bovine serum (FBS) (Sigma Aldrich), while A2780 and PNT2 cells were in RPMI (Aurogene) completed with 10% FBS; a total of 100 units mL^−1^ penicillin and 100 µg·mL^−1^ streptomycin were added to both media; cells were grown in a 5% CO_2_ atmosphere at 37 °C.

When cells arrived at the maximum confluence of 80%, they were harvested with Trypsin (Sigma Aldrich), counted, and seeded in 96 (TTP) or 384 (Sarstedt)-well plates. For 96-well plates, we used 2 × 10^3^ cells·well^−1^ (final volume per well was 100 µL), while for 384-well plates, we used 6 × 10^2^ cells well^−1^ in 30 µL (final volume per well was 30 µL). Treatments were added 12–24 h after the seeding.

Chemical extracts and fractions were dissolved in dimethyl sulfoxide (DMSO) and used for the treatment of cells. The final concentration of the DMSO used was 2% (*v*/*v*) for each treatment. Eighty percent confluent cells were treated in triplicate with fractions of 1, 10 µg mL^−1^, and in duplicate with those at 100 µg mL^−1^, for 24 and 48 h in complete cell medium. Control cells were incubated in complete cell medium with 2% of DMSO.

The antiproliferative effect of samples on cell viability was evaluated using the 3-(4,5-Dimethylthiazol-2-yl)-2,5-diphenyl tetrazolium bromide (MTT) assay (Applichem A2231), according to Gerlier et al. [16]. Briefly, 48 h after treatments, the cell culture media was discarded and substituted with serum-free media containing 5 µg/mL of the MTT solution. The plate was incubated for 3 h at 37 °C in a 5% CO_2_ atmosphere. After incubation, the MTT solution was removed and cells were lysed with isopropanol solution and incubated at room temperature for 15 min in an orbital shaker. The volumes of the MTT solution and isopropanol were adapted according to the type of plate used. Absorbance was read at 570 nm using Infinite M1000Pro (TECAN) or MultiSkan FC (Thermo Scientific, Rodano (MI), Italy) plate readers. The effect of the extracts and fractions at different concentrations was reported as percent of cell viability, calculated as the ratio between the mean absorbance of each treatment and the mean absorbance of control (cells treated with only 2% of DMSO).

### 3.6. Antibacterial Assays

The antibacterial activity was determined by using the following bacterial strains: *Staphylococcus aureus* (ATCC 25923), *Escherichia coli* (ATCC 25922), *Enterococcus faecalis* (ATCC 29212), *Pseudomonas aeruginosa* (ATCC 27853), and *Streptococcus agalactiae* (ATCC 12386). Antibacterial test was performed in sterile Muller Hinton broth for *E. coli*, *P. aeruginosa* and *S. aureus* (Becton Dickinson, Sparks, MD, USA), and in brain heart infusion broth for *E. faecalis* and *S. agalactiae* (Becton Dickinson). The bacterial cultures were obtained from the stock (−80 °C) and were grown on blood agar (acquired from the University hospital, UiT, Tromsø, Norway) at 37 °C for 24 h, and the working bacterial stock culture was maintained at 4 °C. An overnight culture of each strain was prepared and 2 mL of overnight culture was inoculated into 25 mL of growth media and incubated at 37 °C in the shaker at 180 rpm, until the culture reached turbidity, according to 0.5 McFarland standard (1.0 × 10^8^ colony forming unit (CFU)/mL). In this study, bacterial cultures were diluted in 1:100 and then 1:10 in growth media, and the final concentration of bacterial cells in the wells were adjusted to 0.5–3.0 × 10^5^ (CFU)/mL of *S. aureus*, *E. coli*, *E. faecalis*, and *Streptococcus agalactiae* and 3.0–7.0 × 10^4^ CFU/mL of *P. aeruginosa*.

Chemical fractions were solved in MilliQ H_2_O with a 20% *v/v* of DMSO to obtain a final concentration of 1 µg/µL. The test concentration of 50 µL (=50 µg) was transferred into a 96-well microtiter plate (Nunclon^TM^ Delta Surface, Thermo Fisher Scientific, Waltham, MA USA). Subsequently, 50 µL of the final concentration of bacterial cells was added and incubated at 37 °C for 20 h. After incubation, the activity was measured as absorbance at 600 nm on a plate reader (1420 Victor3 TM multilabel counter, Perkin Elmer^®^, JTC MedTech Hub @ MedTech Park, Singapore) and WorkOut 2.0 software (Dazdaq Ltd., Brighton, UK) was used for plate reading. A bacterial suspension with MilliQ water was used as growth control and growth medium, with MilliQ water as media control. In parallel to these tests, a proper MIC assay was performed as a quality control of the sensitivity of the strains, using all the mentioned bacterial strains; testing against dilutions of gentamycin (Amresco, Solon, OH, USA) was used as the reference control for this assay (data not shown). The inhibition was evaluated by the average of the parallel OD value. The OD value ≤0.05 was considered as active and ≥0.09 was considered inactive.

### 3.7. Antidiabetic Assays

To test for an anti-diabetic effect, we applied the enzymatic human recombinant protein tyrosine phosphatase 1B (PTP-1B, Calbiochem) assay using the fluorescent substrate 6,8—difluoro-4-methylumbelliferyl phosphate (DiFMUP, VWR, Leuven, Belgium). Activity is proportional to fluorescence. The assay buffer (pH 7.2) consisted of 25 mM Hepes, 50 mM NaCl, 2 mM Dithriothethreiol, 2.5 mM EDTA, and 0.01 mg/mL Bovine Serum Albumine (BSA). Assay buffer was used as negative control. The positive control consisted of a 160 µM solution of PTP inhibitor IV (Calbiochem) in assay buffer.

The concentration tested (50 µg/mL of extract or fraction) was obtained by diluting 20 µL of the abovementioned stock solution (1000 µg/mL of extract or fraction, with 20% vol of DMSO, see Section 3.6) in 80 µL of the assay buffer solution, and using 25 µL of this working solution in a total reaction volume of 100 µL per well. A total of 50 µL of the PTP-1B enzyme (31.2 ng/mL) was added to the wells, and this was incubated for 30 min to allow any potential interaction between the enzyme and the natural products present. After this first incubation, 25 µL of the 10 µM substrate DiFMUP was added into the wells, reaching the final reaction volume of 100 µL, and was incubated again at 37 °C for 10 min.

Fluorescence was measured using a DTX 880 Multimode Detector (Beckman Coulter, Brea, California, USA), with an excitation wavelength at (λ) 360 nm and emission at 465 nm. Inhibition was calculated by comparing measurements with controls. The “active” threshold was set to be below 30% activity. The PTP-1B assay is very sensitive, and specificity against PTP-1B was checked using the T–Cell-PTP counter screen assay. TC-PTP is essential for life and very similar to PTP-1B, so if inhibited, the hit will not be followed up. The assay is similar to the PTP-1B assay, except that it uses TC-PTP (R&D Systems, Minneapolis, MN 55413, Toll Free USA, Canada) instead of PTP-1B.

With the positive control, the normal function of the enzyme was established (assigned to a 100% threshold), and with the negative control, a severe inhibition was measured (assigned to a 0% threshold). The enzyme activity measurements were expressed as a percentage related to the controls of the respective plate, and calculated as follows: (average of test triplicates − negative control average)/(positive control average − negative control average) × 100.

### 3.8. Statistical Analisys

Statistical analysis on Phyla data was performed by using non-parametric tests (Kruskal–Wallis tests) for each cell line (A2780, A549, PNT2) at a dose of 100, 10 and 1 µg/mL. As the Kruskal–Wallis test is significant, i.e., *p*-value < 0.05, multiple pairwise comparisons between phylum groups, with corrections for multiple testing (Dunnett’s test), were computed to identify which phylum groups were statistically different (signif. codes: 0.0001 ‘****’, 0.001 ‘***’, 0.01 ‘**’, 0.05 ‘*’). For our purpose, we show only the difference between phylum levels and the control group CTRL- (see Figure 2A).

Similarly, Kruskal–Wallis tests followed up by Dunn’s test were applied to identify which species X, B, C, D, E, including the control group (CTRL-), were different for each phylum, cell line (A2780, A549, PNT2), and dose (100, 10 and 1 µg/mL).

## 4. Conclusions

Since the first studies in the early 1960s, MNPs have proved to be a prolific source of drug candidates, featuring a large chemical diversity and a wide array of biological activities. The early development of these molecules has had to face several challenges, including the supply issue and the development of suitable methodologies for chemical and biological screenings. Thus, despite the progress made in the last decade, there is a continuous need to explore novel strategies and innovative methods of assessing or examining the therapeutic potential of these products.

In this work, we tested a library of marine extracts constituted by raw and SPE-derived fractions against cancer cells, bacterial strains, and cell receptors for potential anti-diabetic evaluation. Although the SPE samples were composed of heterogeneous classes of molecules, the pre-fractionation significantly increased the chance of detecting positive hits. This was particularly evident for the antibacterial and antidiabetic candidates that were detectable only after fractionation. SPE fractionation also reduced the chemical variability of small molecules in each fraction, thus simplifying the steps related to the purification and identification of the active compounds. We focused the analysis on the major eukaryotic phyla so far explored in marine bioprospecting. An analysis of the positive hits confirmed sponges as the most prolific source of antitumoral and antibiotic activities, but it also suggested that other phyla are more suitable in addressing other therapeutic targets.

## Figures and Tables

**Table 1:** Cultured microorganisms used to prepare the MNP library.

Phylum	Taxon	Ident Code	Origin
Bacillariophyta	*Cyclotella cryptica*	CBC25A	CCMP331 (NCMA)
Bacillariophyta	*Thalassiosira pseudonana*	CBC36A	CCMP1335 (NCMA)
Bacillariophyta	*Skeletonema marinoi*	CBC40A	CCMP2092 (NCMA)
Bacillariophyta	*Thalassiosira rotula*	CBC41A	New isolate (Gulf of Naples, Western Mediterranean Sea)
Bacillariophyta	*Phaeodactylum tricornutum*	CBC42A	New isolate (Gulf of Naples, Western Mediterranean Sea)
Chlorophyta	*Tetraselmis suecica*	CBC20A	Neoalgae
Chlorophyta	*Tetraselmis* sp.	CBC27A	Phytobloom
Haptophyta	*Isochrysis galbana*	CBC21A	Phytobloom
Ochrophyta	*Nannochloropsis* sp.	CBC24A	Phytobloom
Miozoa	*Amphidinium massartii*	CBC39A	New isolate (Gulf of Naples, Western Mediterranean Sea)
Miozoa	*Amphidinium carterae*	CBC35A	New isolate (Fusaro lake, brakish lagoon)
Miozoa	*Heterocapsa* sp.	CBC53A	New isolate (Gulf of Naples, Western Mediterranean Sea)
Miozoa	*Scrippsiella* sp.	CBC55A	New isolate (Gulf of Naples, Western Mediterranean Sea)

**Table 2:** Benthic organisms used to prepare the MNP library. The biological samples were collected in the Mediterranean Sea and Antarctic Ocean.

Phylum	Taxonomy/Voucher Code	Identification Code	Collection Site
Bryozoa	*Bugula neritina* complex	CBC28A	Gulf of Naples, Western Mediterranean Sea
Bryozoa	*Amathia verticillata*	CBC30A	Gulf of Naples, Western Mediterranean Sea
Bryozoa	*Schizoporella errata*	CBC31A	Gulf of Naples, Western Mediterranean Sea
Bryozoa	Lepraliellidae ind.	CBC44A	Gulf of Naples, Western Mediterranean Sea
Bryozoa	ITA-AM 15 12/13	CBC48A	Antarctica
Chlorophyta	*Codium bursa*	CBC33A	Gulf of Naples, Western Mediterranean Sea
Chordata (Tunicata)	*Ciona robusta*	CBC18A	Gulf of Naples, Western Mediterranean Sea
Chordata (Tunicata)	*Halocynthia papillosa*	CBC19A	Gulf of Naples, Western Mediterranean Sea
Chordata (Tunicata)	*Styela plicata*	CBC22A	Gulf of Naples, Western Mediterranean Sea
Chordata (Tunicata)	*Ascidiella aspersa*	CBC29A	Off the coast of Crotone, Ionian Sea
Chordata (Tunicata)	*Botryllus schlosseri* complex	CBC37A	Gulf of Naples, Western Mediterranean Sea
Chordata (Tunicata)	ITA-AM 12 12/13	CBC50A	Antarctica
Cnidaria	*Leptogorgia sarmentosa*	CBC23A	Gulf of Naples, Western Mediterranean Sea
Cnidaria	ITA-AM 13 12/13	CBC51A	Antarctica
Cnidaria	ITA-AM 14 12/13	CBC62A	Antarctica
Mollusca	*Philine quadripartita*	CBC26A	Off the coast of Crotone, Ionian Sea
Mollusca	*Hexaplex trunculus* complex	CBC34A	Gulf of Naples, Western Mediterranean Sea
Mollusca	*Euthria cornea*	CBC38A	Gulf of Naples, Western Mediterranean Sea
Mollusca	*Gasteropteron rubrum*	CBC46C	Off the coast of Crotone, Ionian Sea
Mollusca	*Gasteropteron rubrum*	CBC46A	Western Mediterranean Sea
Mollusca	*Scaphander lignarius* complex	CBC47A	Off the coast of Crotone, Ionian Sea
Ochrophyta	*Cystoseira dubia*	CBC49A	Off the coast of Crotone, Ionian Sea
Ochrophyta	*Dictyota* sp.	CBC45A	Gulf of Naples, Western Mediterranean Sea
Porifera	*Haliclona mediterranea*	CBC1A	Gulf of Naples, Western Mediterranean Sea
Porifera	*Chondrosia reniformis*	CBC2A	Gulf of Naples, Western Mediterranean Sea
Porifera	*Crambe crambe*	CBC3A	Gulf of Naples, Western Mediterranean Sea
Porifera	*Chondrilla nucula*	CBC4A	Gulf of Naples, Western Mediterranean Sea
Porifera	ITA-AM 09 12/13	CBC10A	Antarctica
Porifera	*Spongia* sp. (ITA-AM 06 16)	CBC12A	Off the coast of Crotone, Ionian Sea
Porifera	ITA-AM 07 02	CBC11A	Antarctica
Porifera	ITA-AM 08 02	CBC15A	Antarctica
Porifera	ITA-AM 02 02	CBC13A	Antarctica
Porifera	ITA-AM 04 02	CBC14A	Antarctica
Porifera	ITA-AM 05 02	CBC16A	Antarctica
Porifera	ITA-AM 03 02	CBC17A	Antarctica
Porifera	*Spirastrella cunctatrix*	CBC32A	Gulf of Naples, Western Mediterranean Sea
Porifera	ITA-AM 10 12/13	CBC56A	Antarctica
Porifera	ITA-AM 11 12/13	CBC57A	Antarctica
Porifera	*Dendrilla* sp. (GAN-38)	CBC74A-1	Antarctica
Porifera	*Dendrilla* sp. (GAN-54)	CBC74B	Antarctica
Porifera	*Dendrilla* sp. (GAN-53)	CBC74A-3	Antarctica
Porifera	GAN-3	CBC75A	Antarctica
Porifera	GAN-37	CBC77A	Antarctica
Rhodophyta	*Pterocladiella capillacea*	CBC43A	Gulf of Naples, Western Mediterranean Sea

**Table 3:** Percentage contribution of each SPE fraction to the total loaded extract considered for the taxonomic groups represented in the library. Recovery of total organic compounds (OCs) was calculated as the sum of the SPE fractions B–E.

Phylum	Frac. A %	Frac. B %	Frac. C %	Frac. D %	Frac. E%	Total OCs %
Bacillariophyta	39.8 ± 9.3	11.7 ± 6.4	11.4 ± 3	10.4 ± 4.2	15.2 ± 10.4	48.7
Bryozoa	71.3 ± 8.2	3.2 ± 2.0	3.6 ± 1.2	2.1 ± 0.7	5.3 ± 2.0	14.2
Chlorophyta	52.6 ± 18.8	5.0 ± 6.1	8.0 ± 6.7	5.8 ± 4.3	6.6 ± 5.0	25.4
Chordata (Tunicata)	72.3 ± 9.7	2.6 ± 0.8	3.3 ± 1.7	3.1 ± 1.8	3.9 ± 1.8	12.9
Cnidaria	44.1 ± 12.9	3.6 ± 1.0	4.1 ± 1.4	5.8 ± 1.5	7.0 ± 3.6	20.6
Haptophyta	49.4	8.4	0.9	16.9	7.8	34.0
Miozoa	44.7 ± 8.1	3.9 ± 2.3	6.8 ± 4.1	8.4 ± 4.5	9.0 ± 5.1	28.0
Mollusca	63.7 ± 7.2	3.9 ± 0.9	3.9 ±1.4	2.9 ± 1.3	5.8 ± 2.4	16.5
Ochrophyta	56.1 ± 10.6	1.8 ± 2.4	4.1 ± 3.6	4.2 ± 4.7	6.2 ± 6.3	16.4
Porifera	62.0 ± 17.4	4.7 ± 2.1	3.7 ± 2.4	3.8 ± 2.7	5.8 ± 3.0	18.1
Rhodophyta	71.0	1.1	2.9	0.7	1.2	5.9

## Supplementary Materials

The following are available online at https://www.mdpi.com/article/10.3390/md19110640/s1, Figure S1: Non-parametric test and Post-hoc analysis: for each cell line (A2780, A549, PNT2) and dose (100, 10, 1 µg/mL), we computed the Kruskal–Wallis test (non-parametric test). (1) As the p-value was less than the significance level 0.05, we can conclude that there are significant differences between the Phylum. (2) A multiple pairwise comparison between Phylum is then performed to calculate pairwise comparisons between Phylum levels, with corrections for multiple testing (Dunnett’s Test). (3) We show only the comparisons vs. CTRL; Figure S2. Barplot: Log Fold-Change for significant comparisons (vs. CTRL-); Figure S3: Heatmap showing the percentage of vitality on the three cell lines (A549, A2780, PTN2) after treatment with the raw extracts (X) and the corresponding four SPE fractions (B, C, D, E) at 1 and 10 µg/mL; File S1 (cytotoxicity data); File S2 (antibacterial data); File S3 (antidiabetic data).
