# Supplementary material for: Probing the Therapeutic Potential of Marine Phyla by SPE Extraction
Source: Mar Drugs. 2021 Nov 16;19(11):640. doi: 10.3390/md19110640 (PMC8625500; doi:10.3390/md19110640)

MTT assay of the in-house library of Marine Natural Products on PNT2 (prostate epithelium immortalized with SV40), A549 (pulmonary adenocarcinoma) and A2780 (ovarian carcinoma) cell lines after 48h of incubation. The results were expressed as percentage of vitality after treatment with extracts (X) and related SPE fractions (B-E), compared to untreated cells at 1 (orange bars), 10 (green bars), and 100 μg/mL (brown bars).

CBC 1 A: (Porifera)

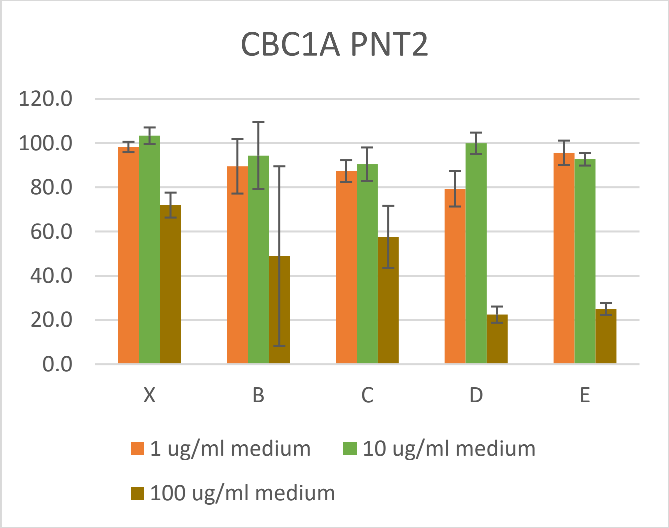

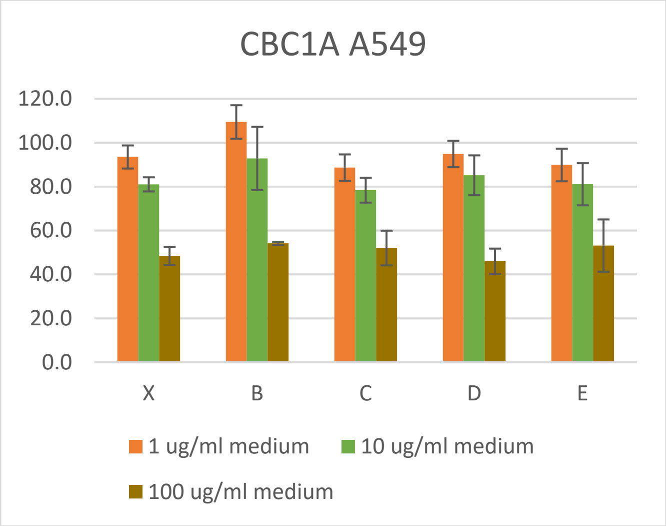

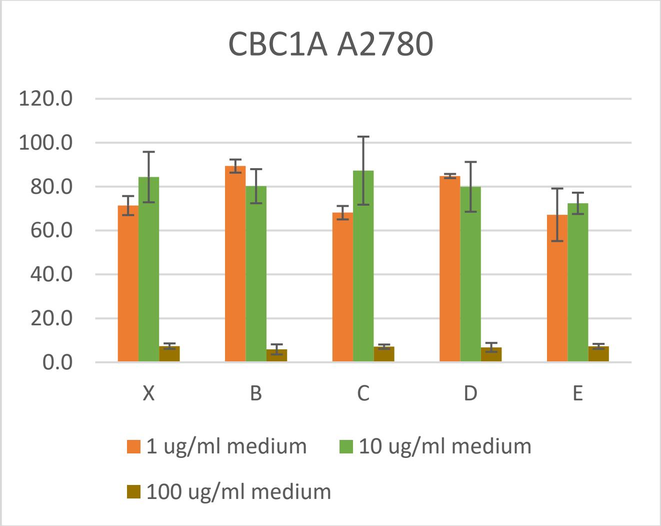

CBC 2 A: (Porifera)

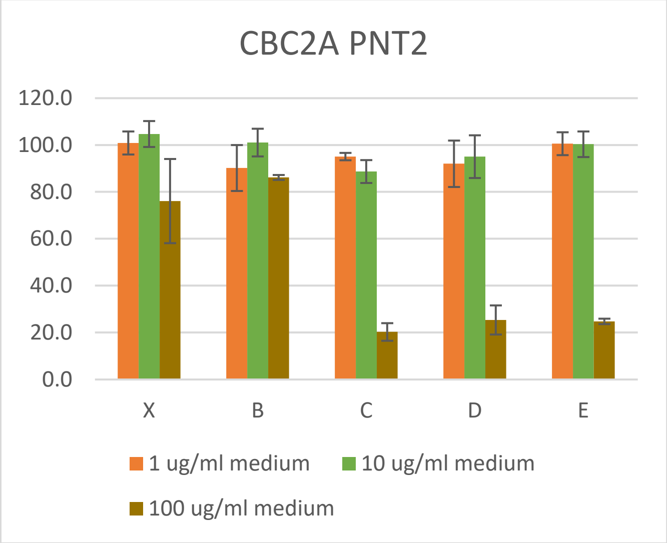

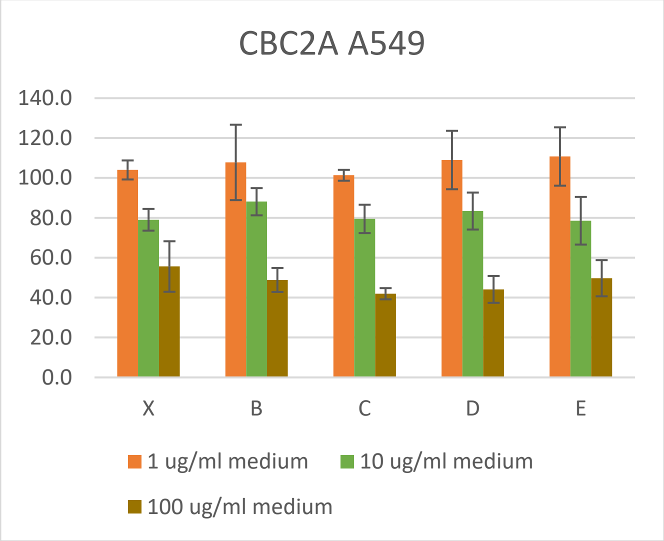

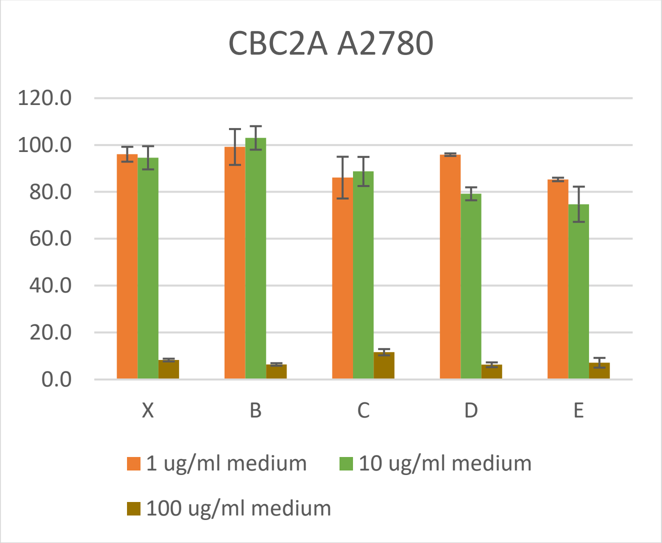

CBC 3 A: (Porifera)

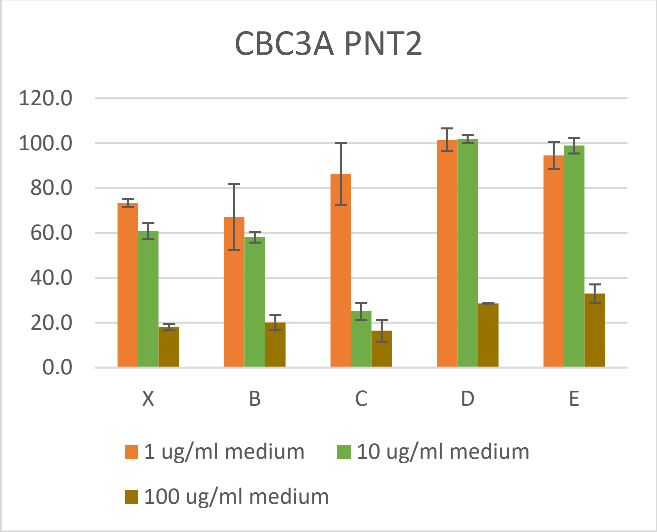

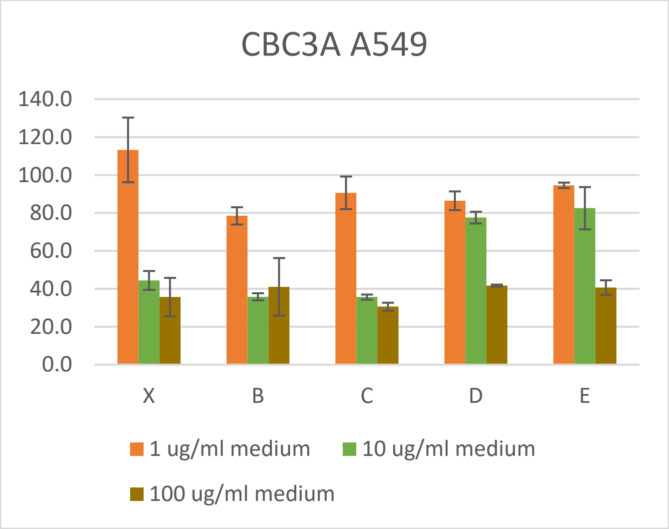

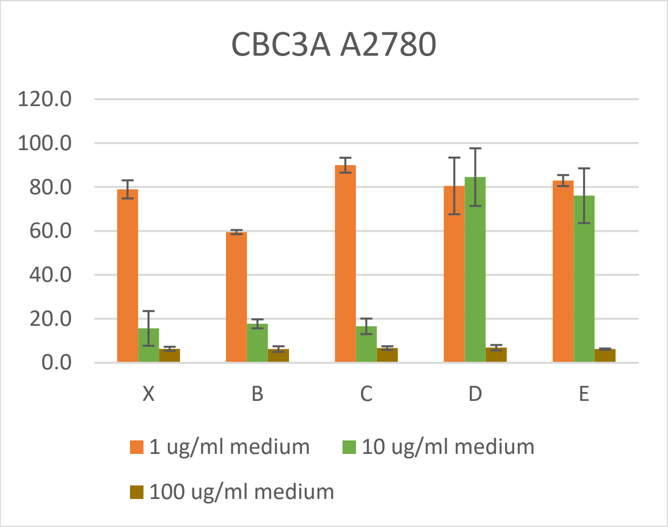

CBC 4 A: (Porifera)

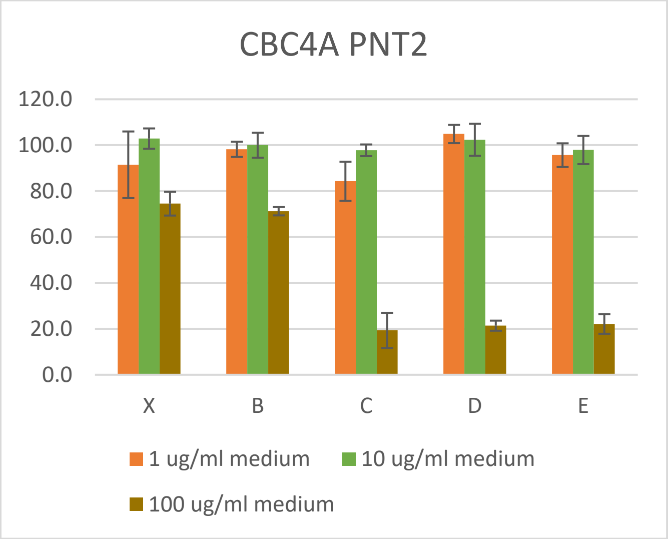

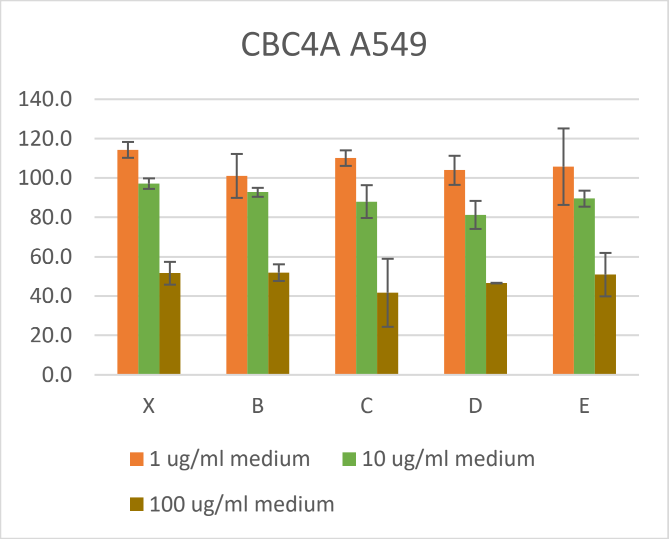

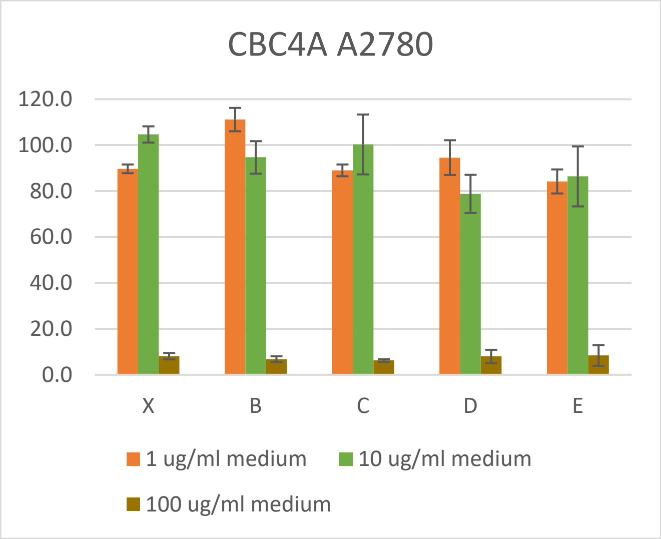

CBC 10 A: (Porifera)

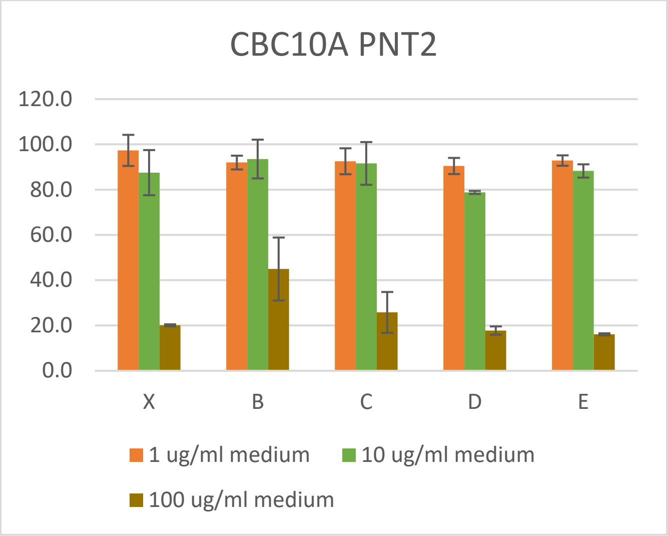

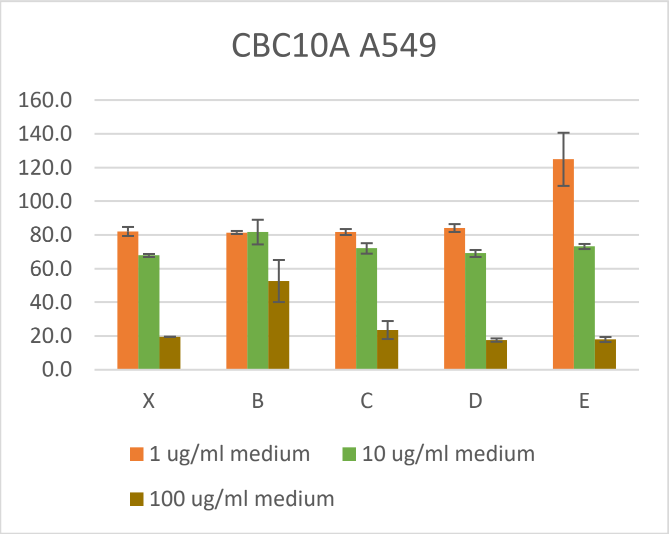

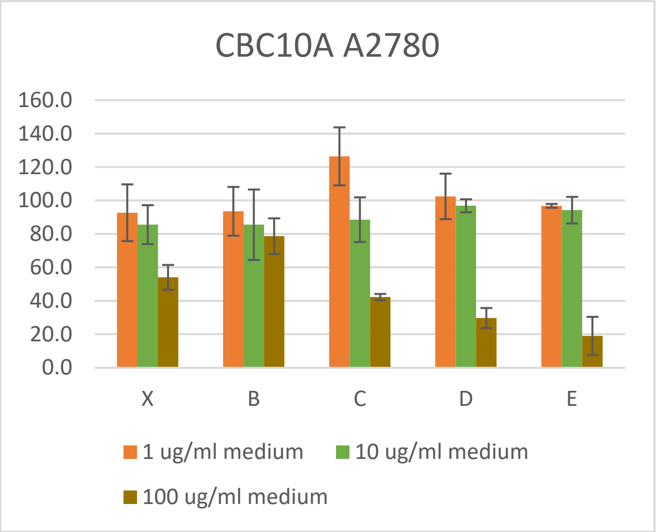

CBC 11 A: (Porifera)

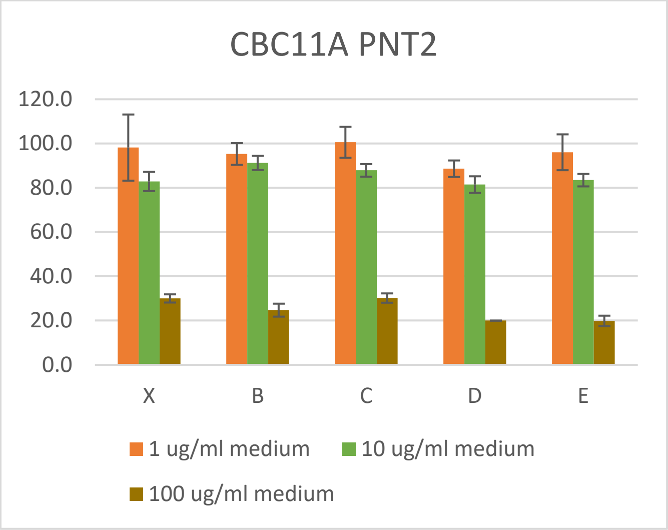

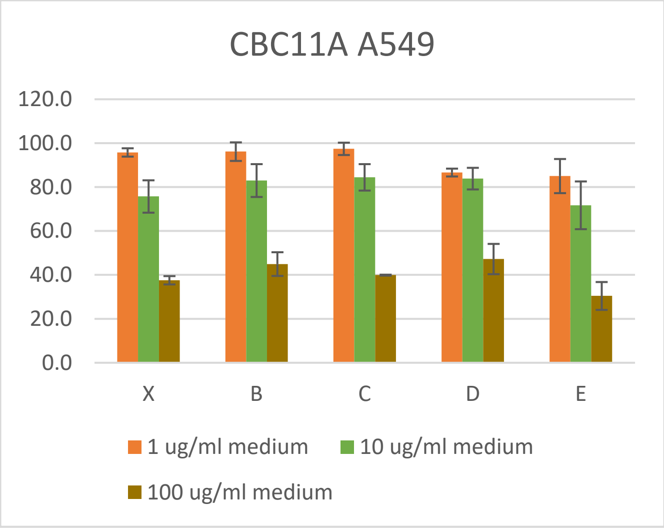

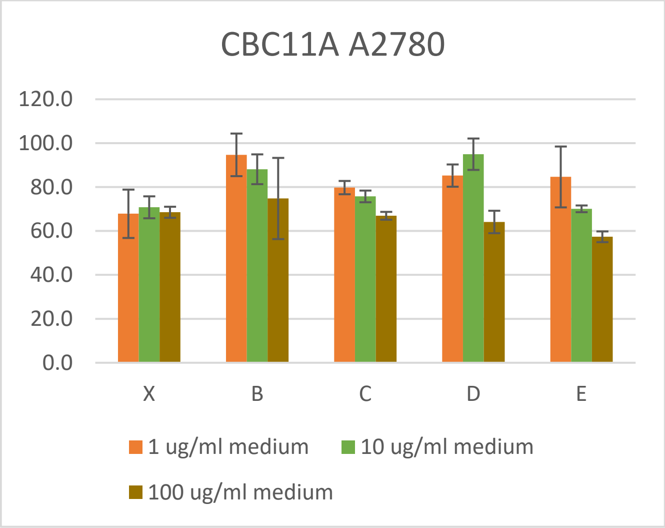

CBC 12 A: (Porifera)

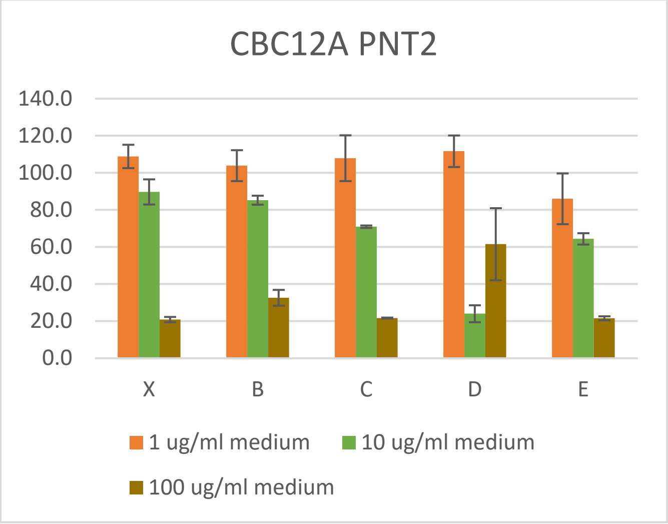

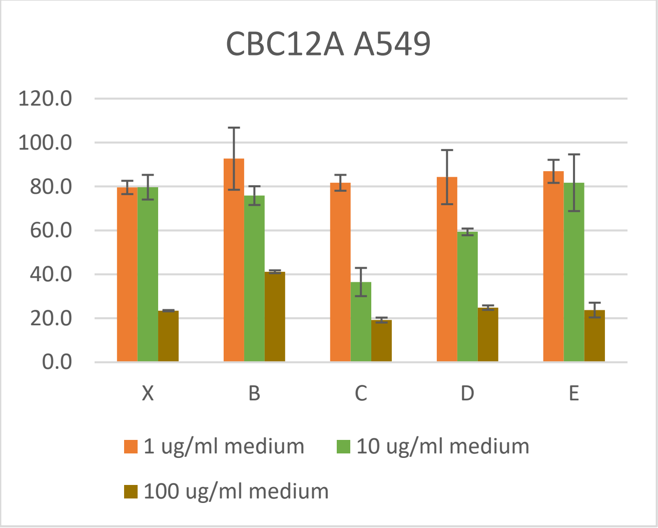

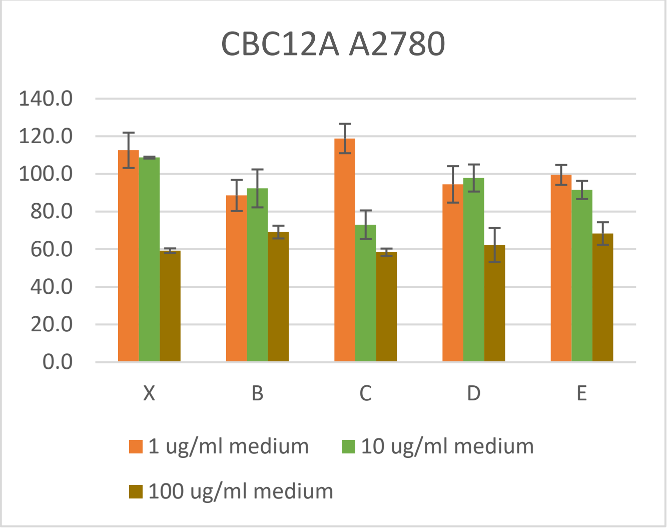

CBC 13 A: (Porifera)

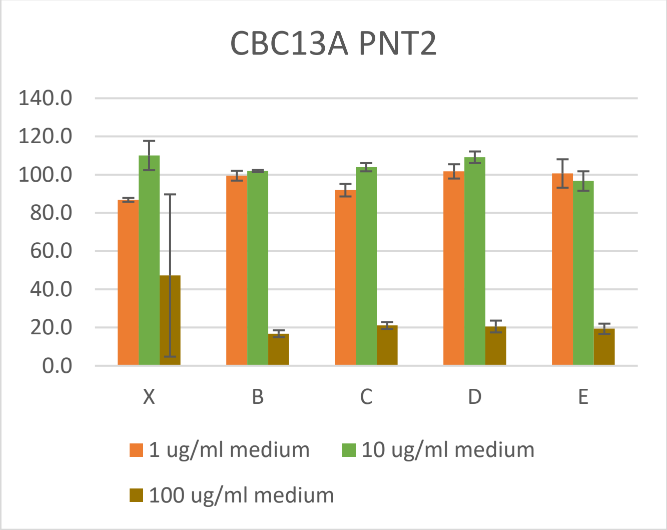

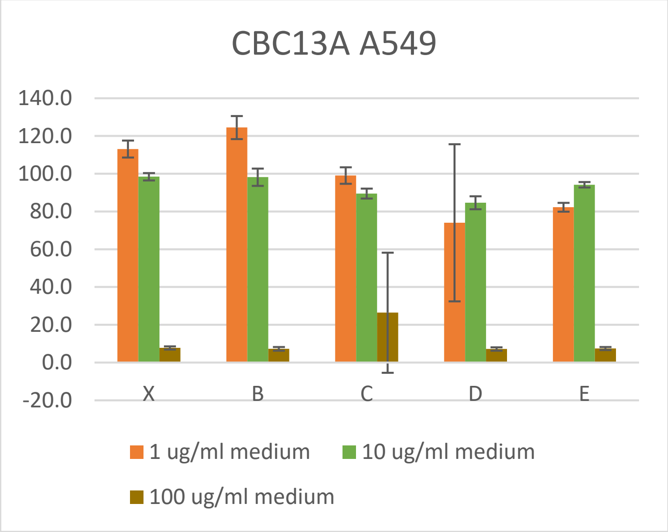

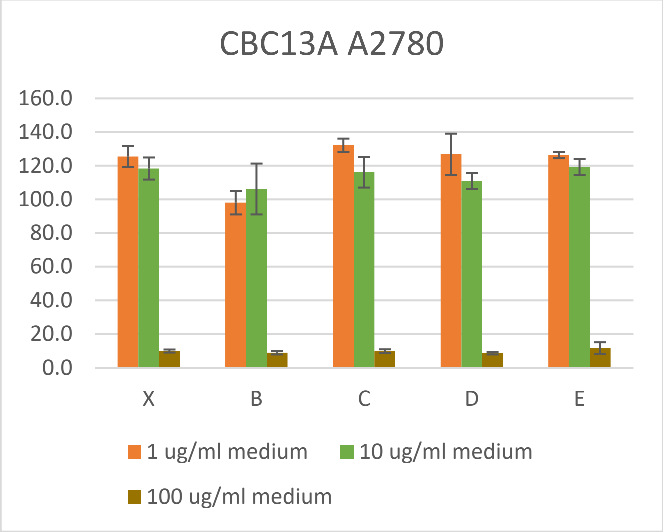

CBC 14 A: (Porifera)

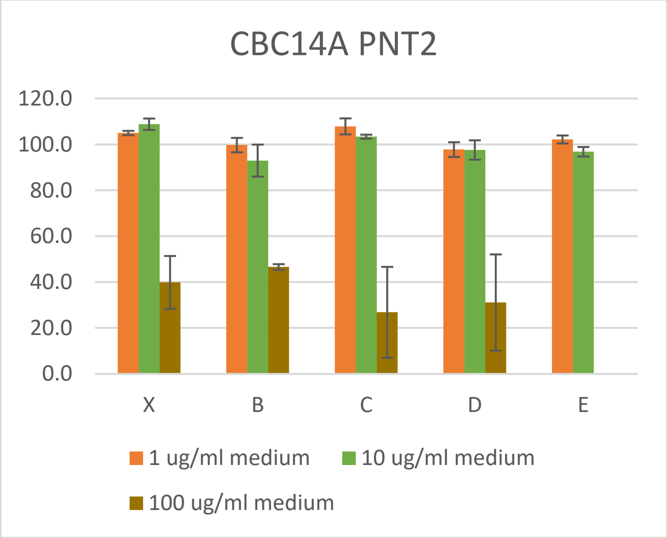

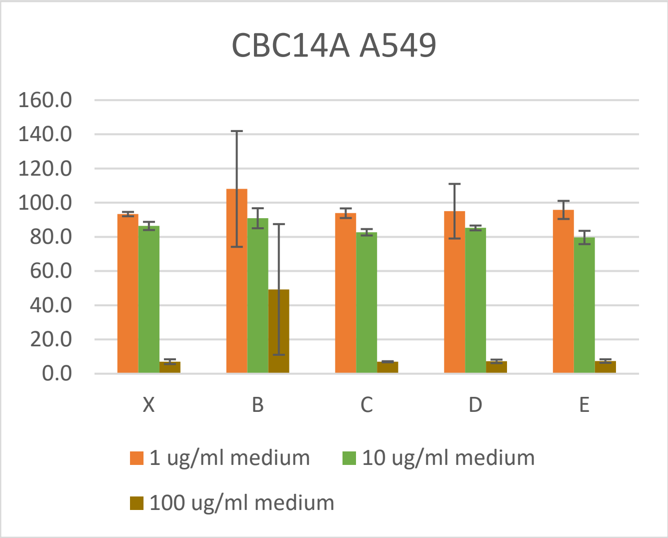

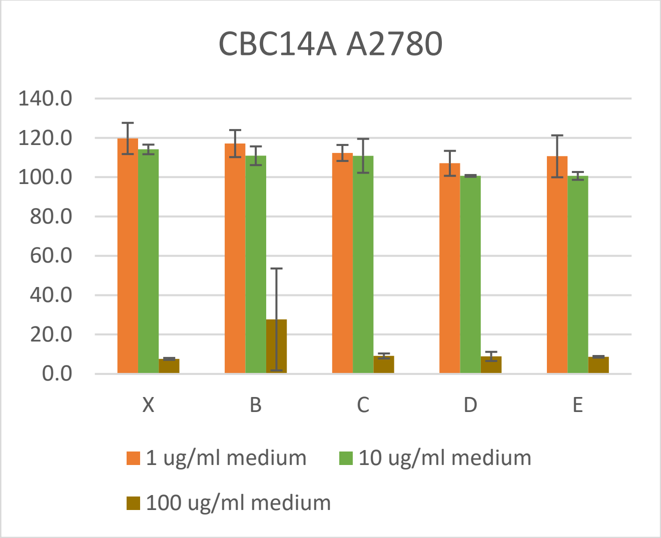

CBC 15 A: (Porifera)

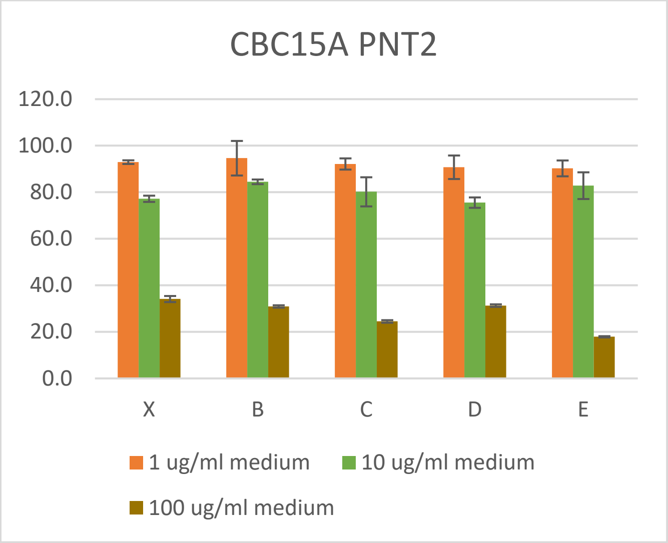

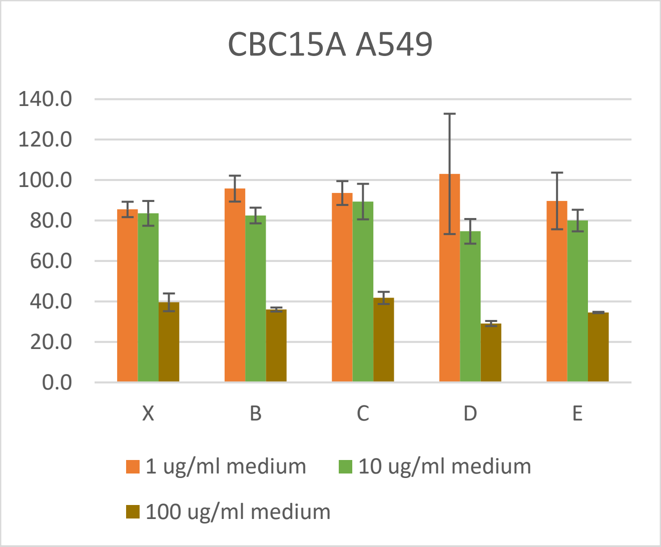

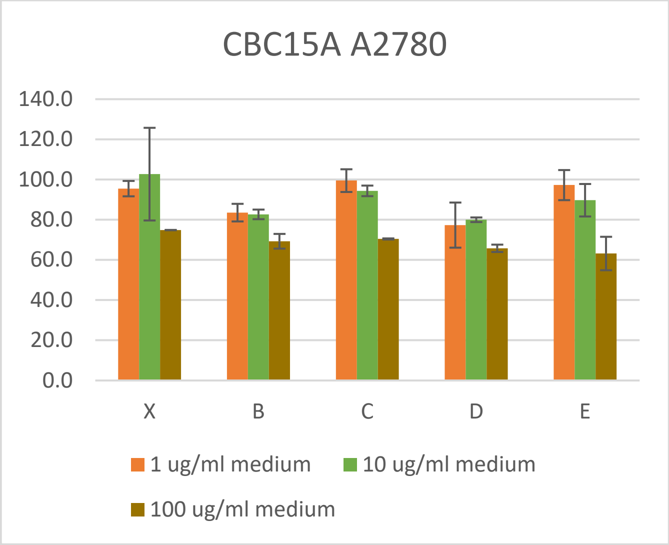

CBC 16 A: (Porifera)

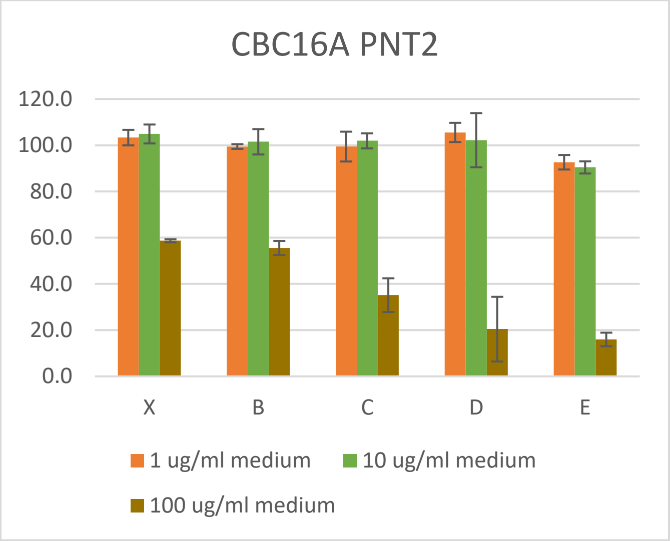

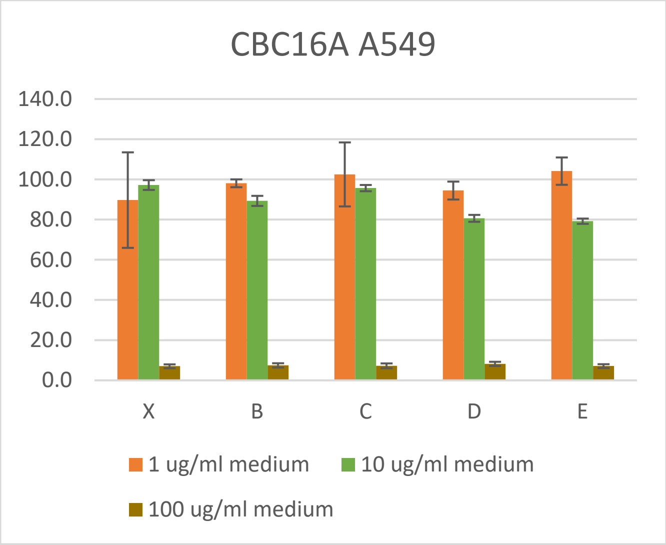

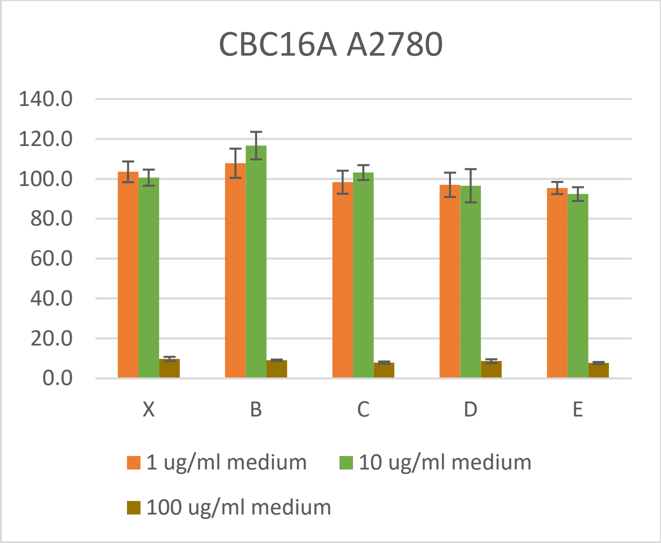

CBC 17 A: (Porifera)

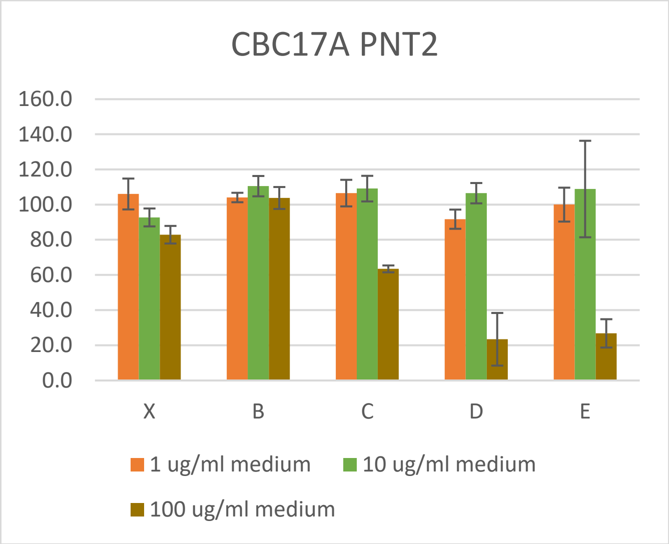

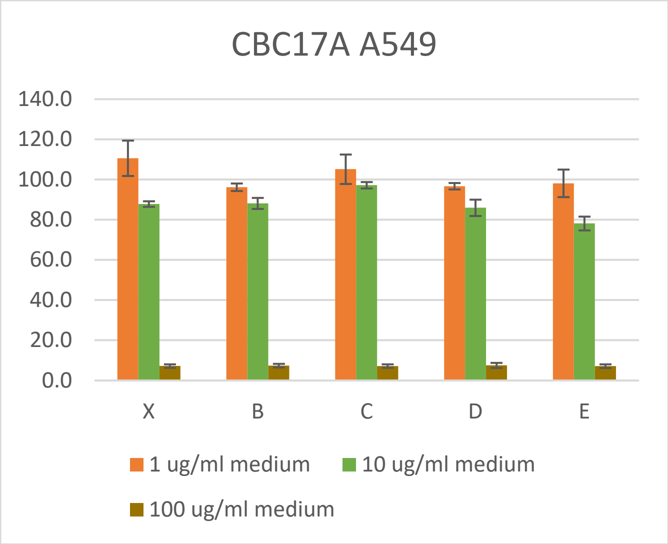

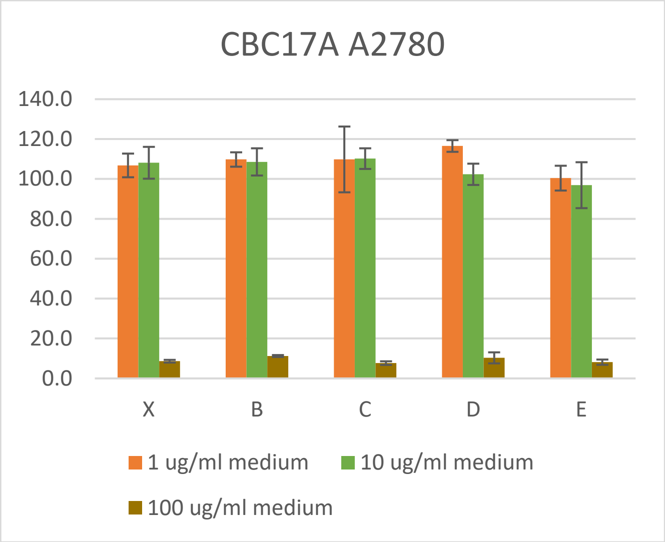

CBC 18 A: (Chordata)

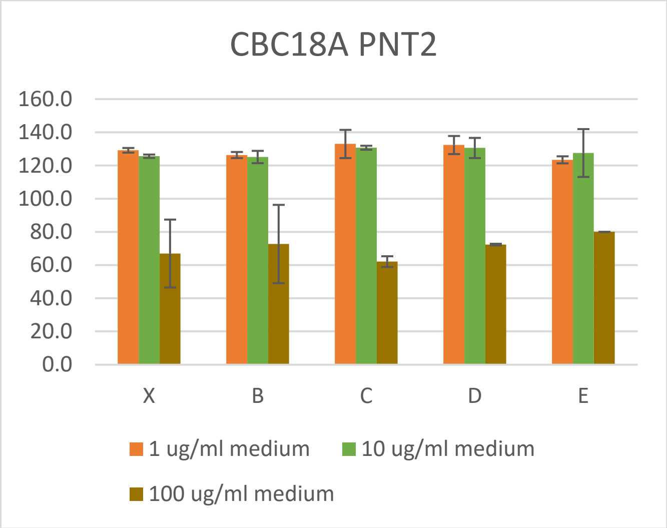

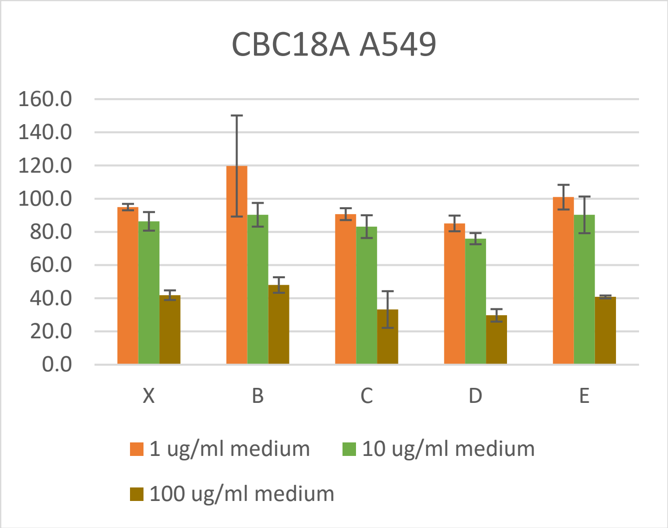

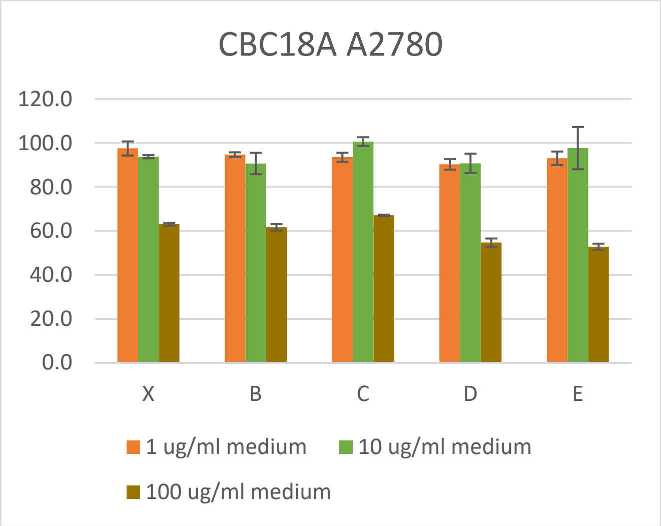

CBC 19 A: (Chordata)

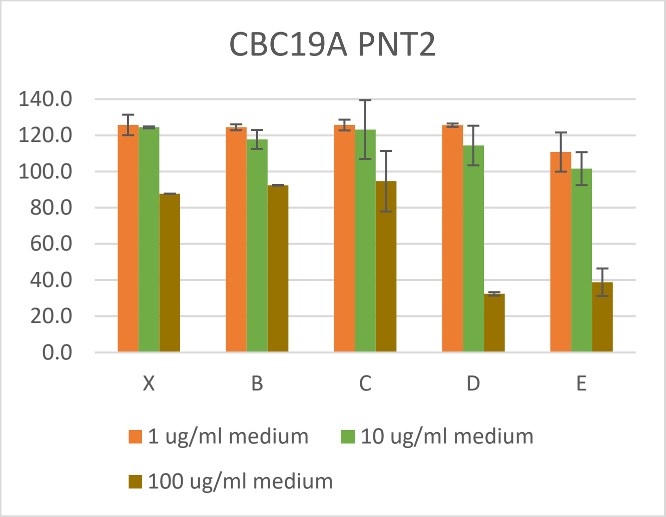

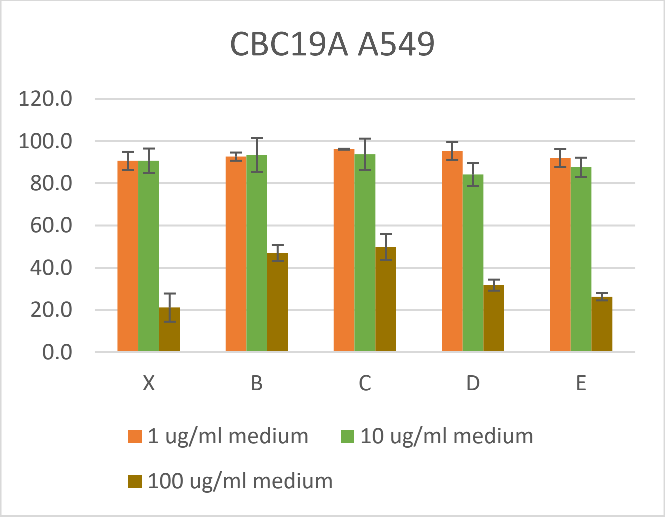

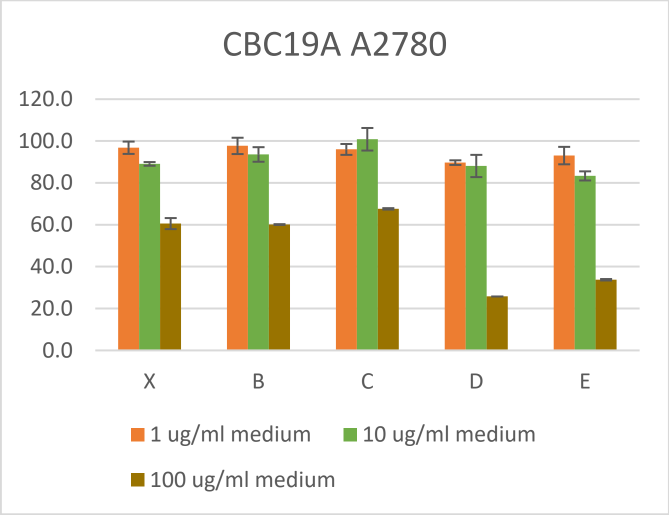

CBC 20 A: (Chlorophyta)

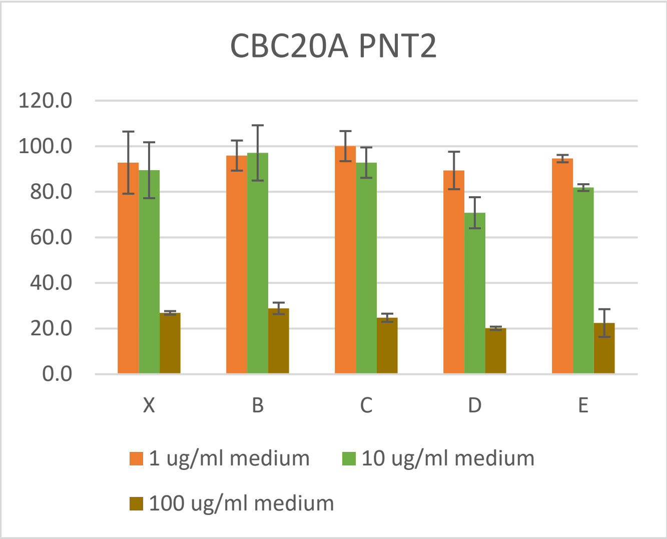

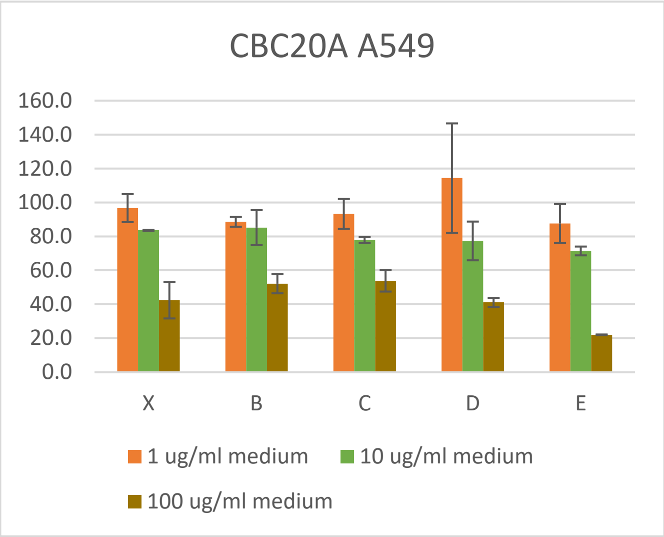

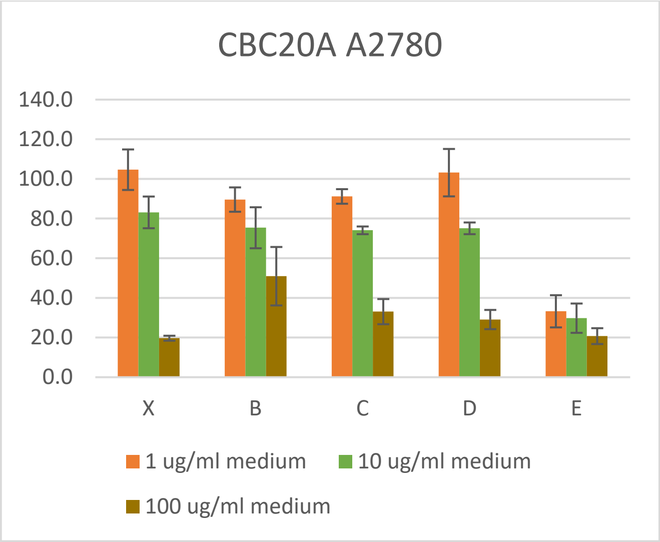

CBC 21 A: (Haptophyta)

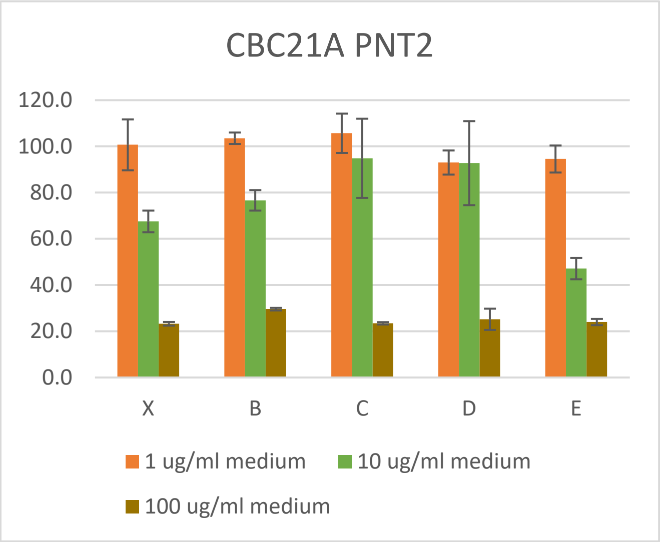

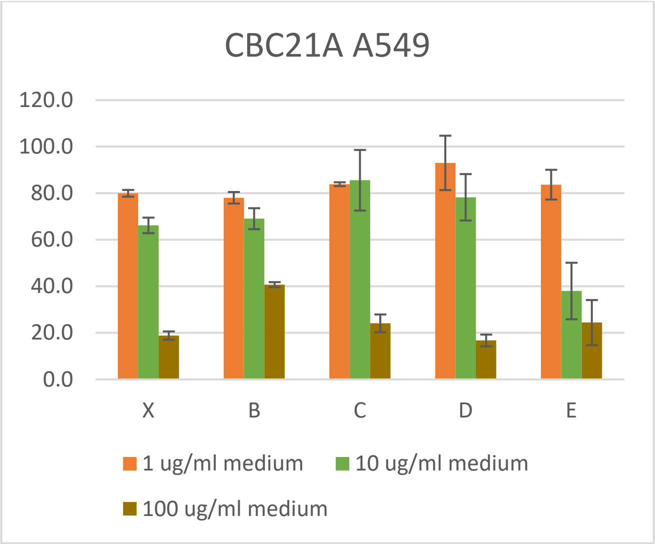

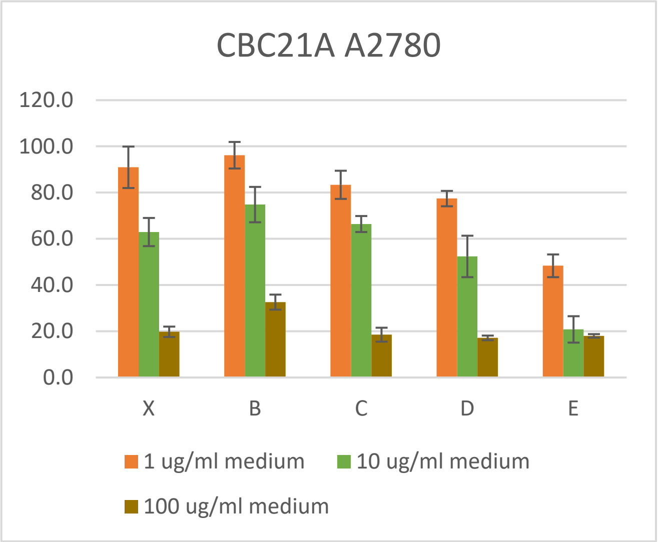

CBC 22 A: (Chordata)

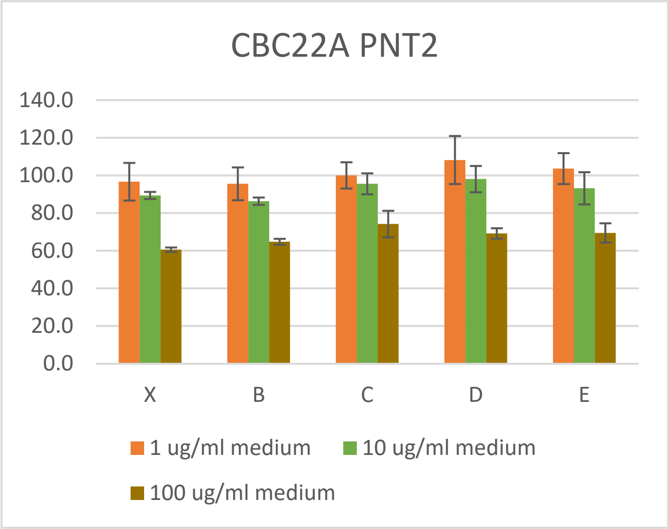

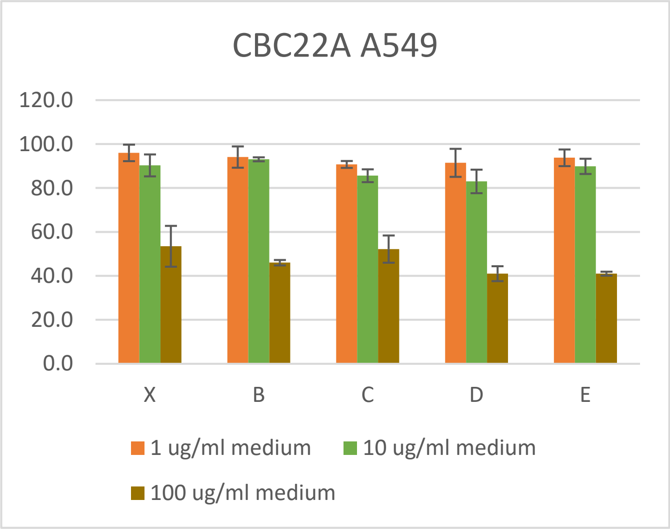

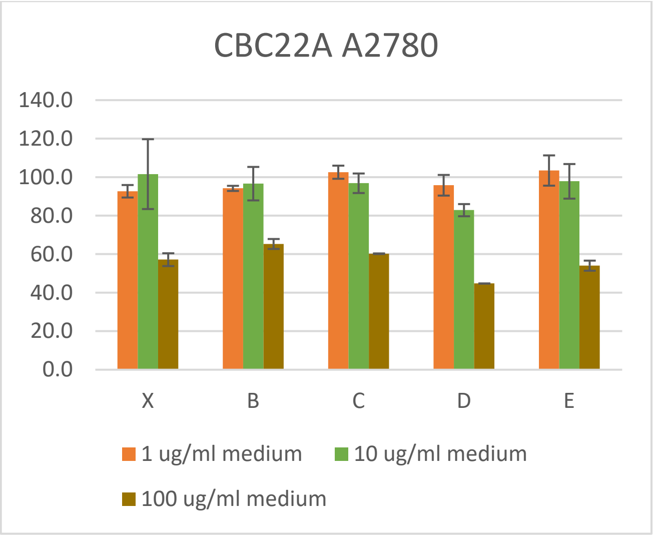

CBC 23 A: (Cnidaria)

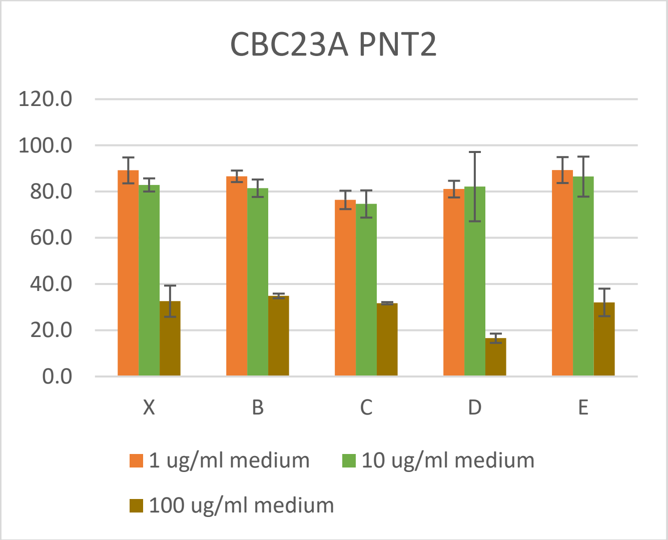

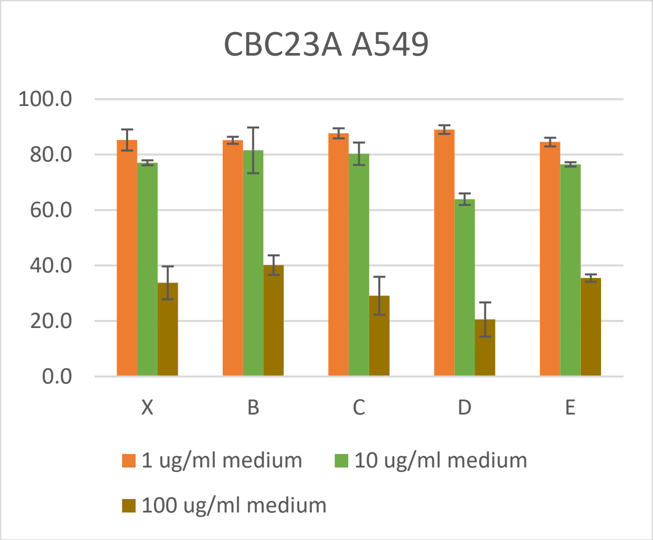

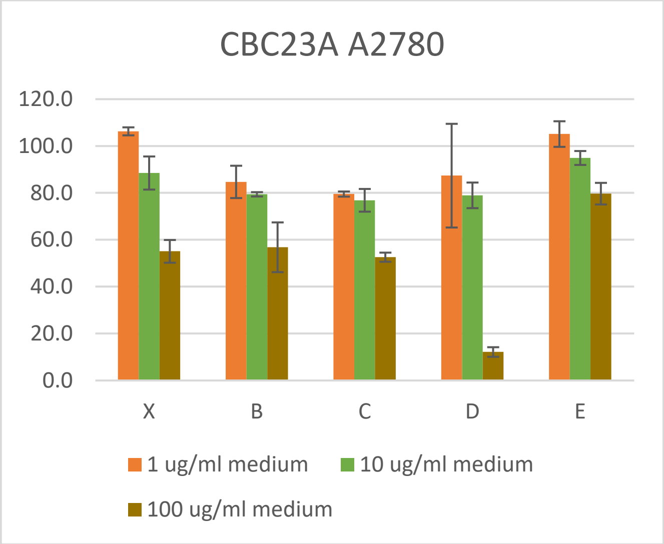

CBC 24 A: (Ochrophyta)

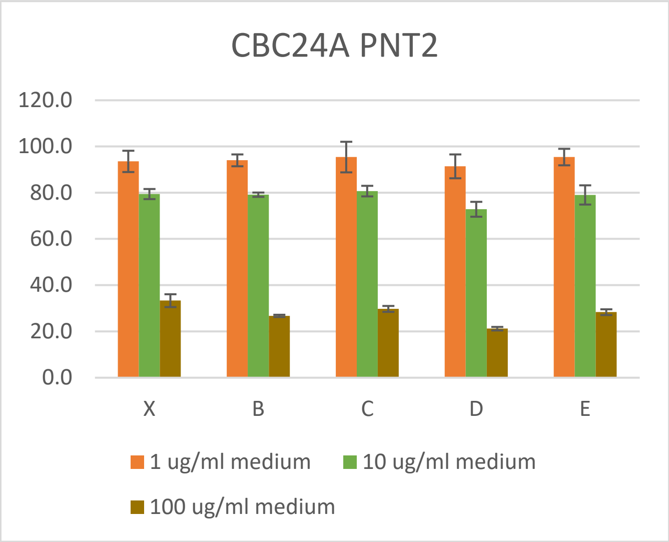

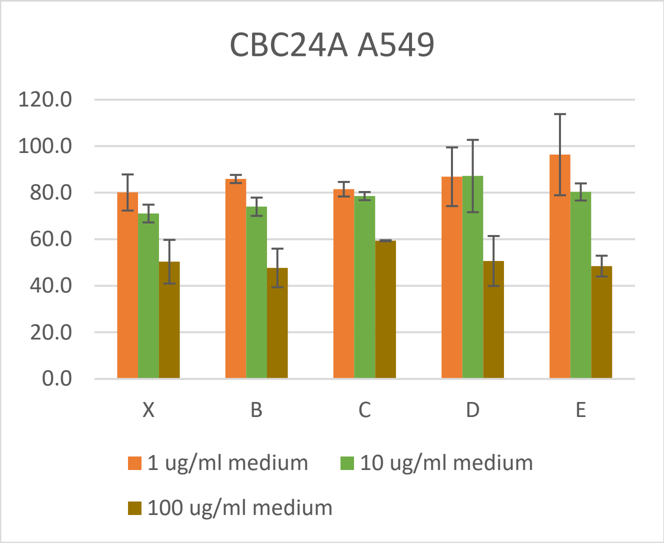

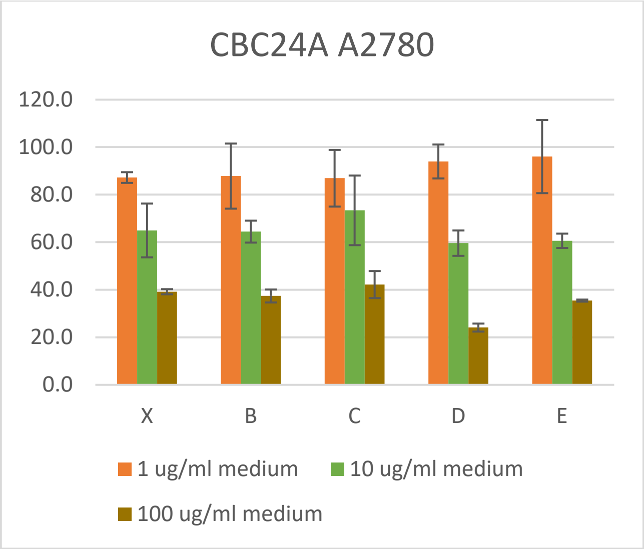

CBC 25 A: (Bacillariophyta)

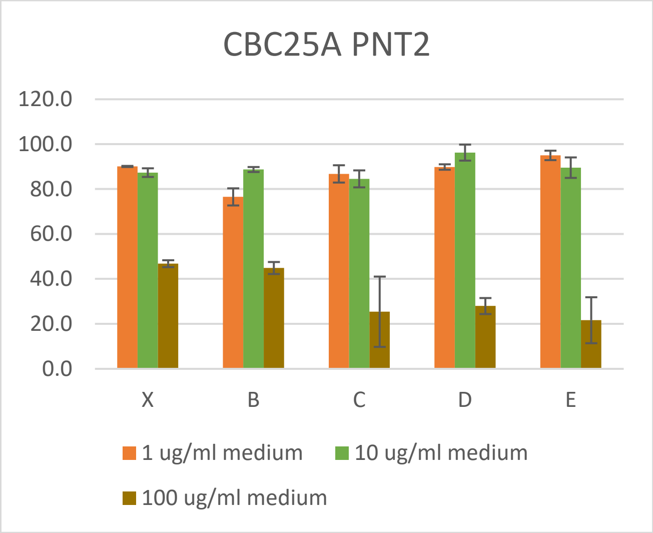

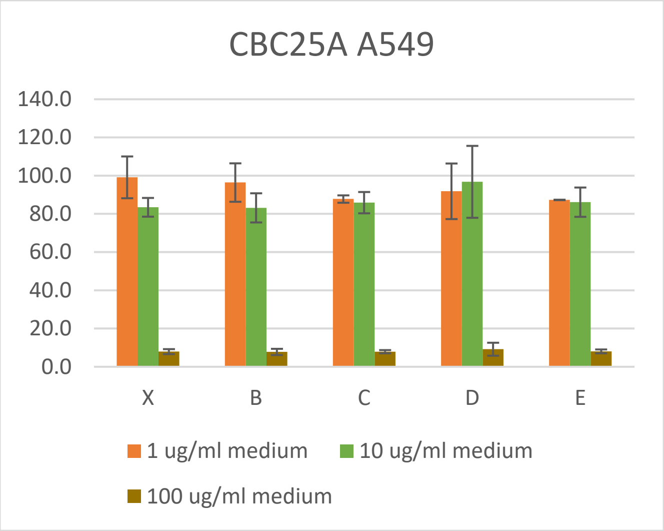

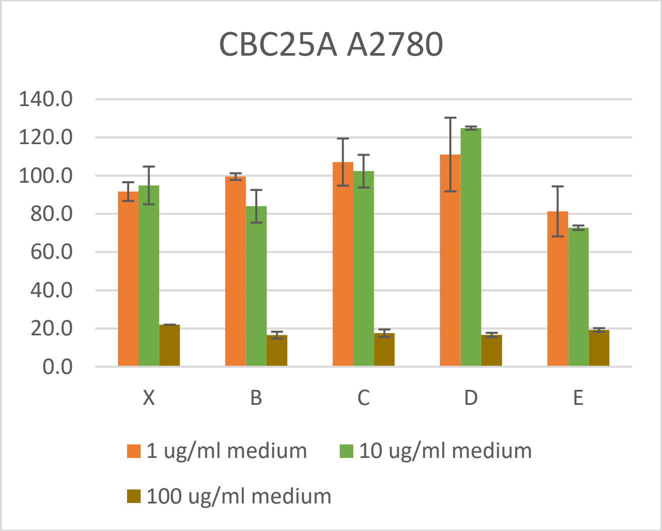

CBC 26 A: (Mollusca)

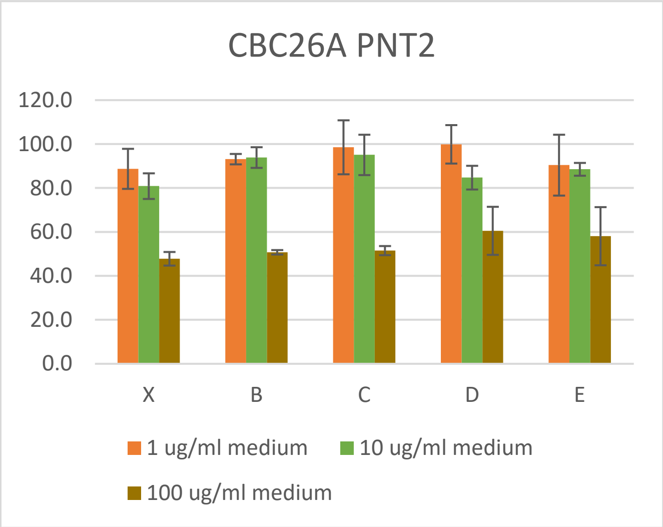

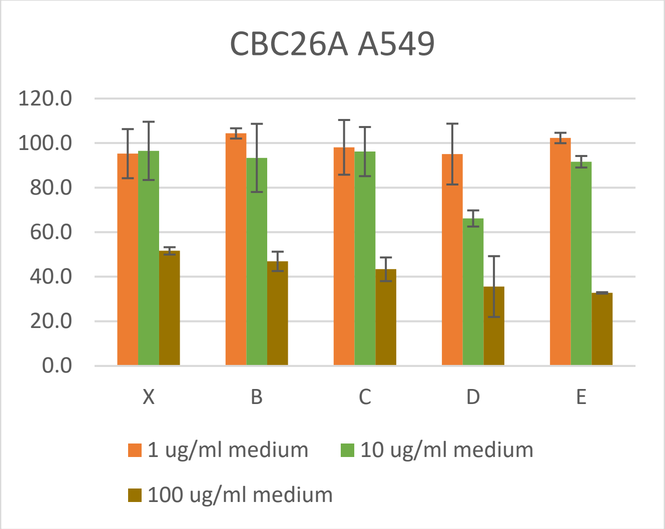

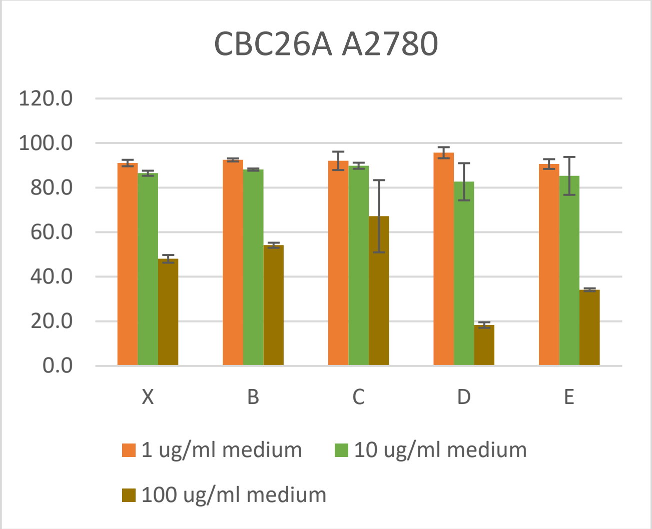

CBC 27 A: (Chlorophyta)

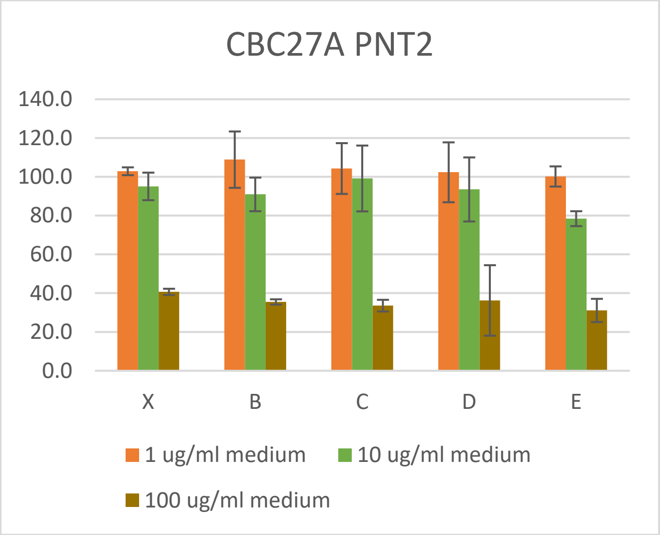

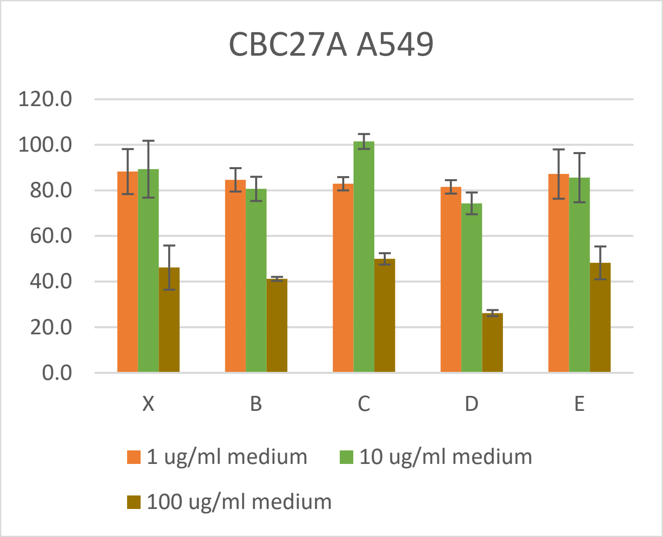

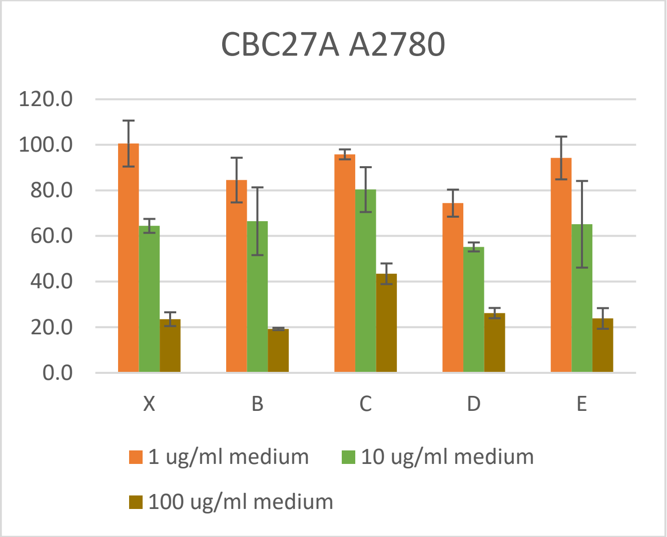

CBC 28 A: (Briozoa)

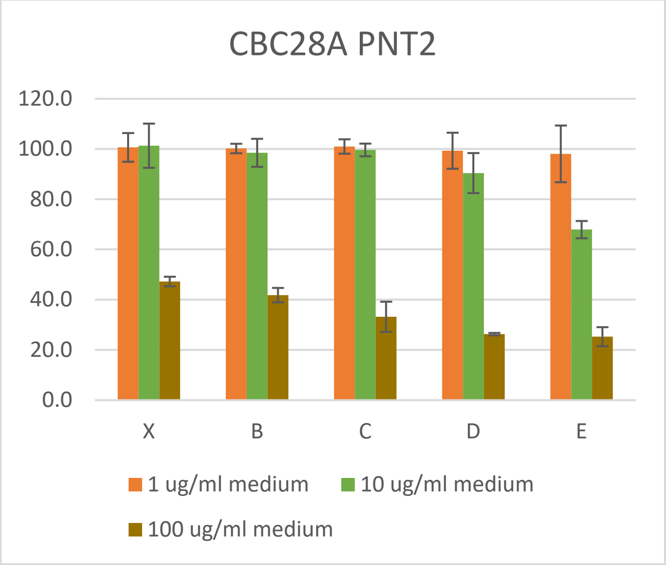

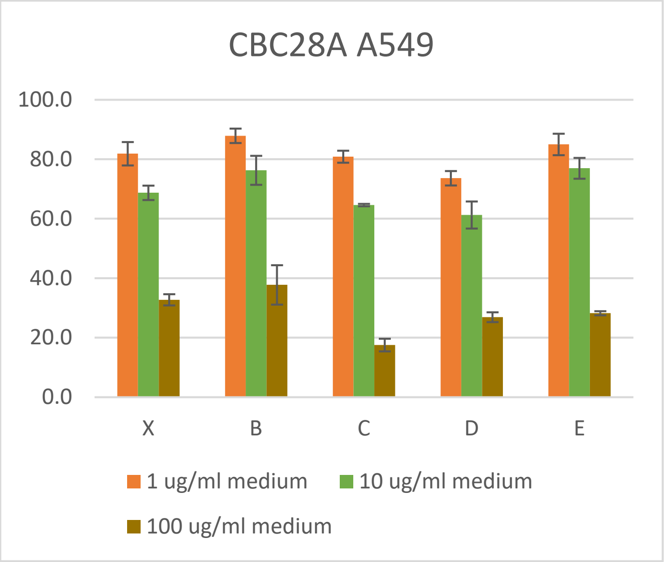

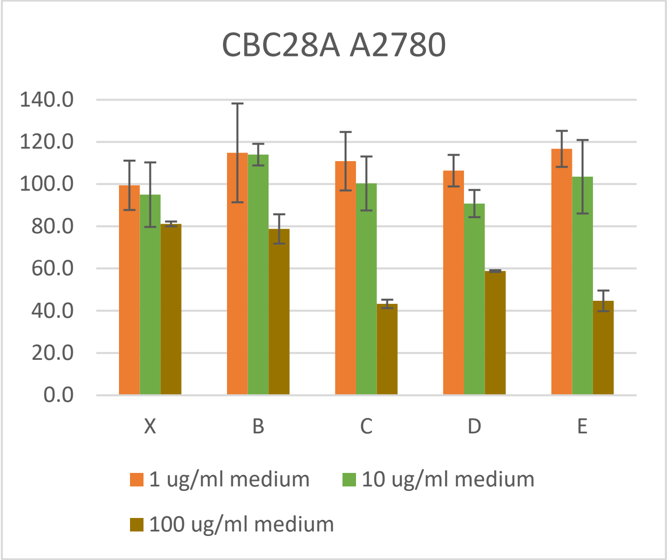

CBC 29 A: (Chordata)

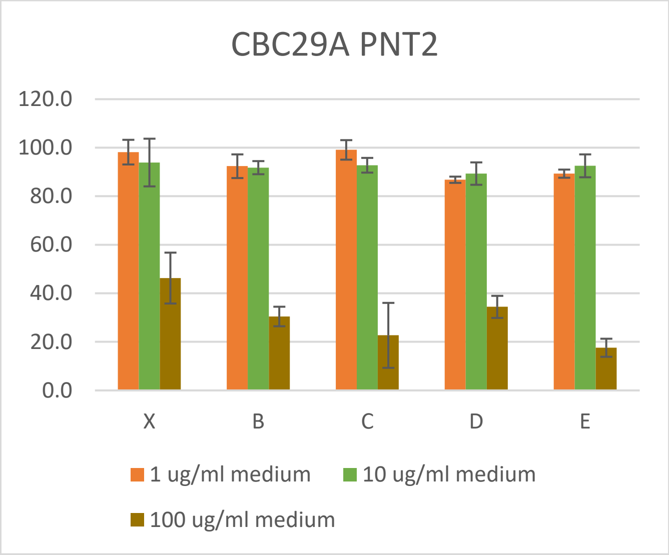

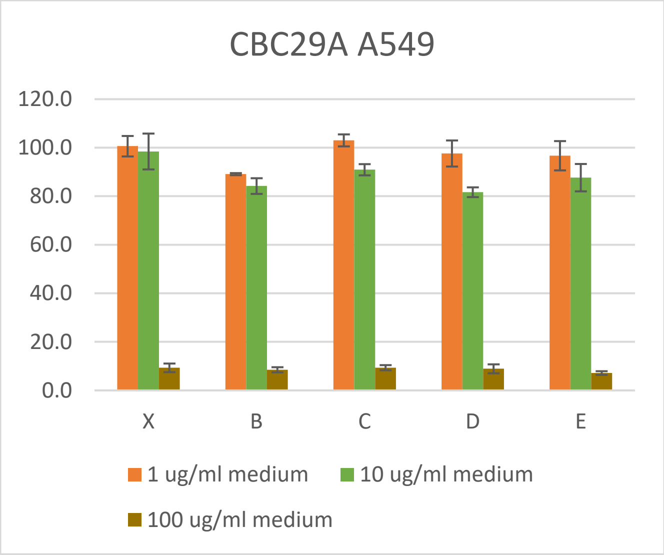

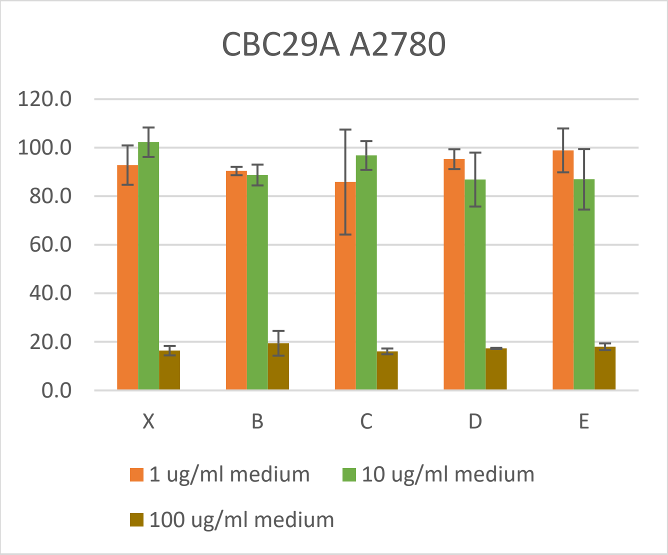

CBC 30 A: (Briozoa)

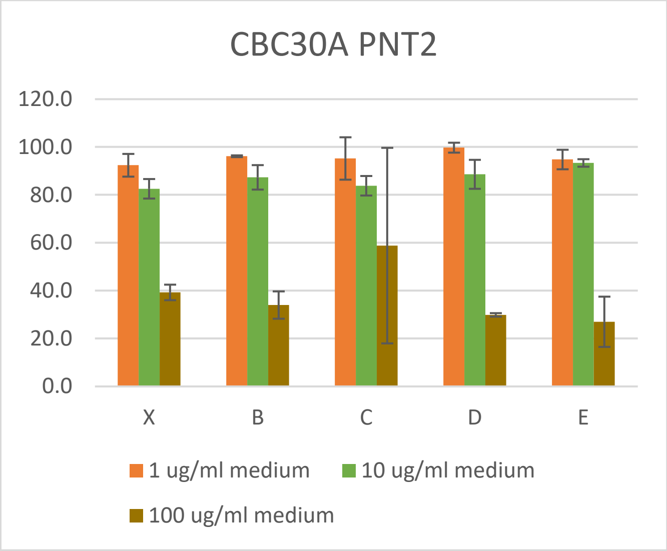

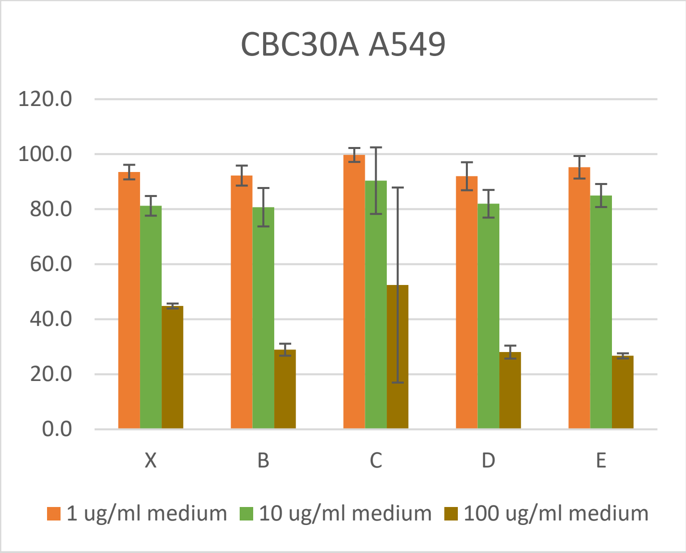

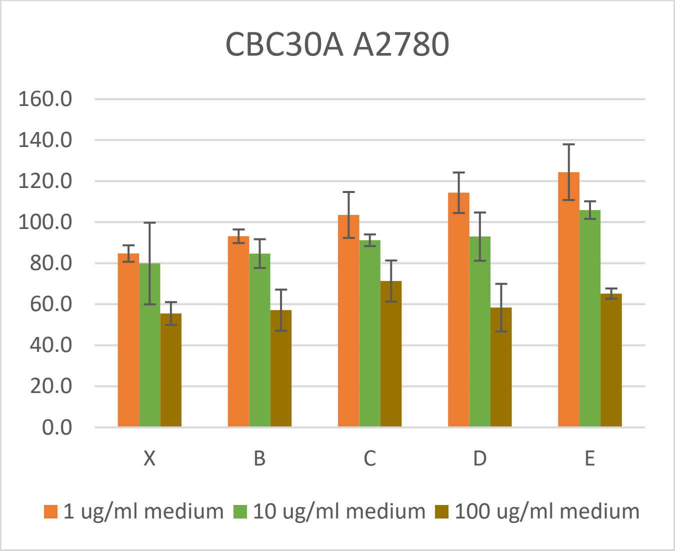

CBC 31 A: (Briozoa)

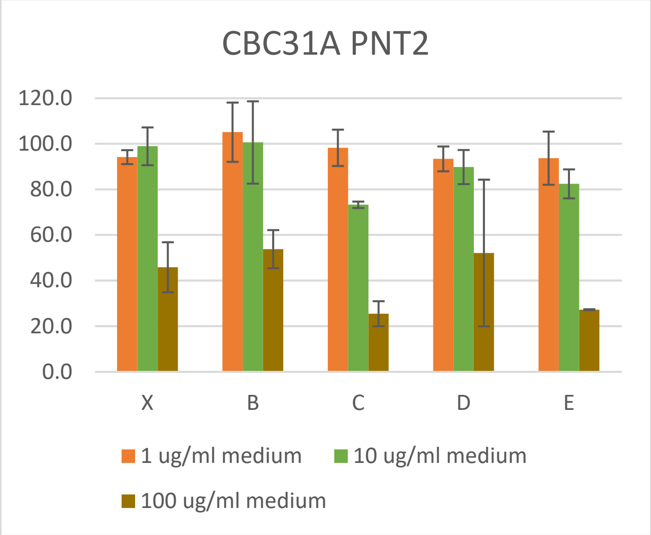

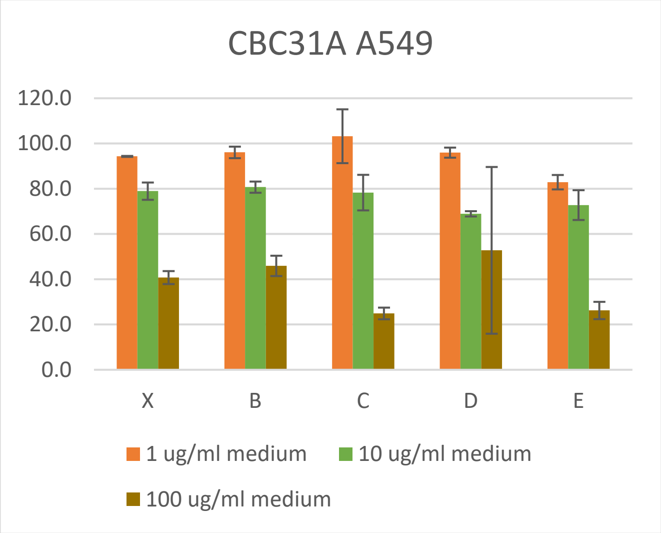

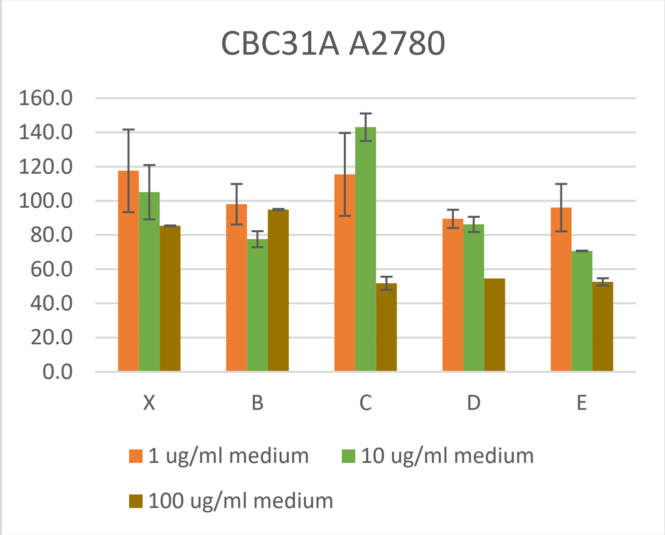

CBC 32 A: (Porifera)

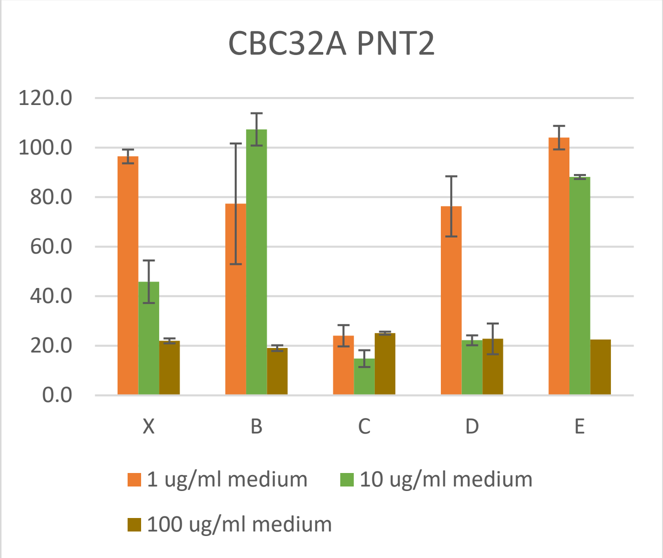

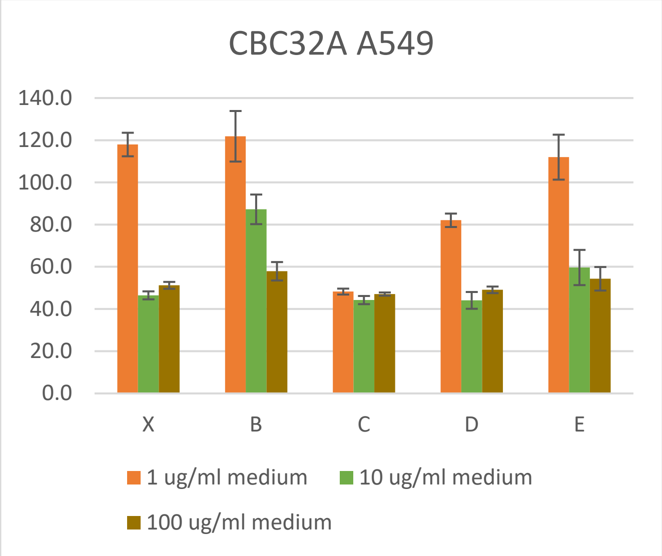

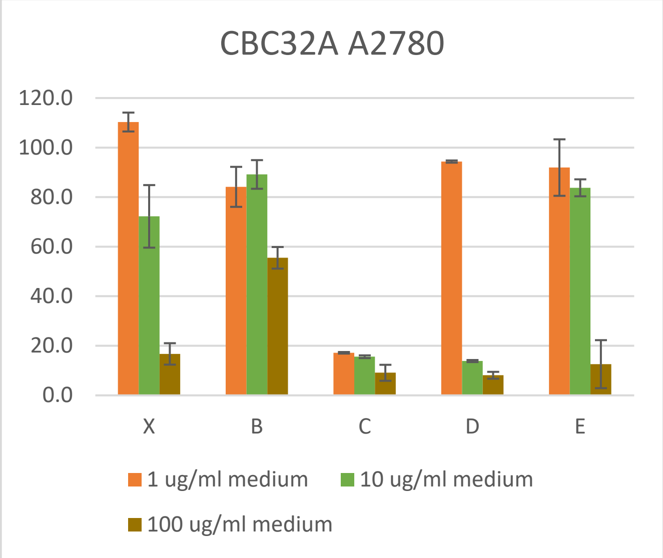

CBC 33 A: (Chlorophyta)

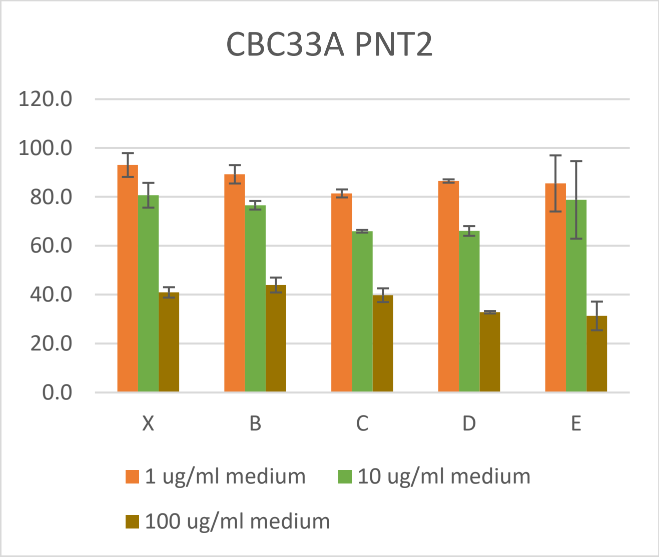

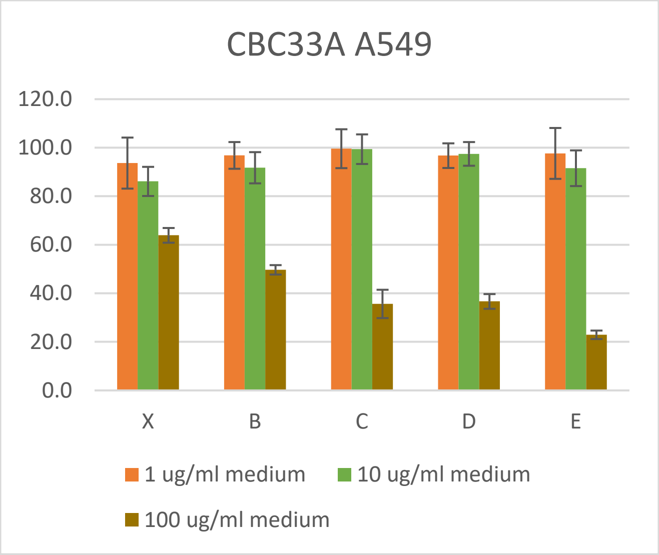

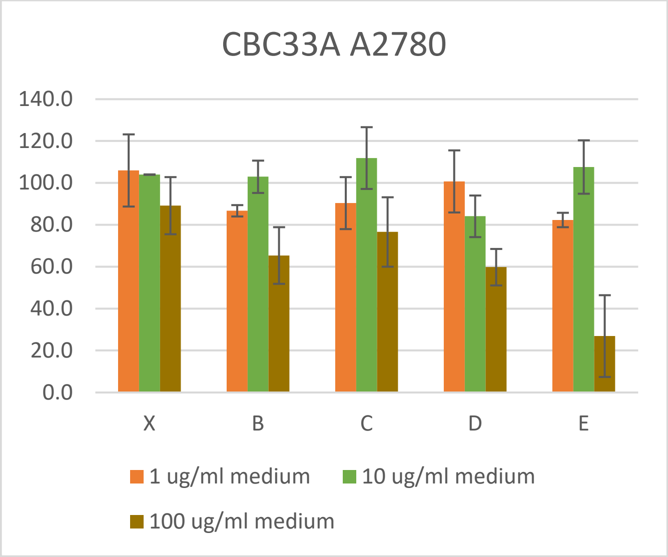

CBC 34 A: (Mollusca)

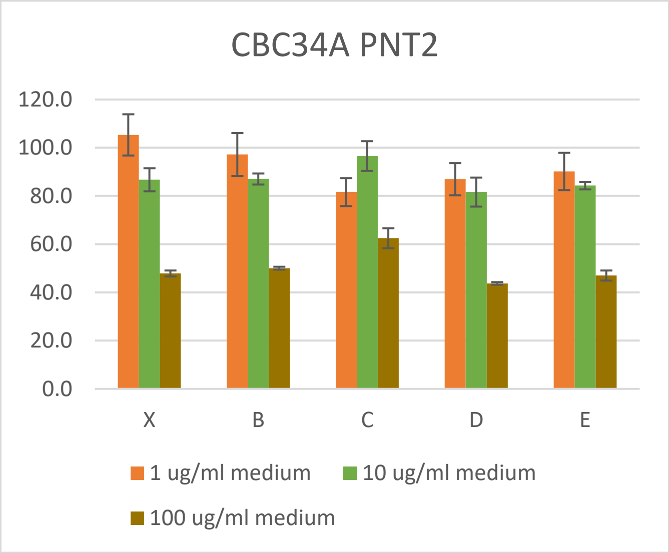

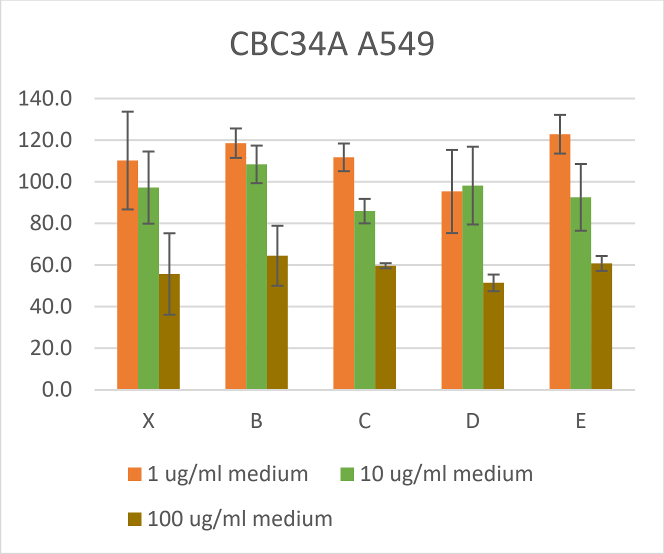

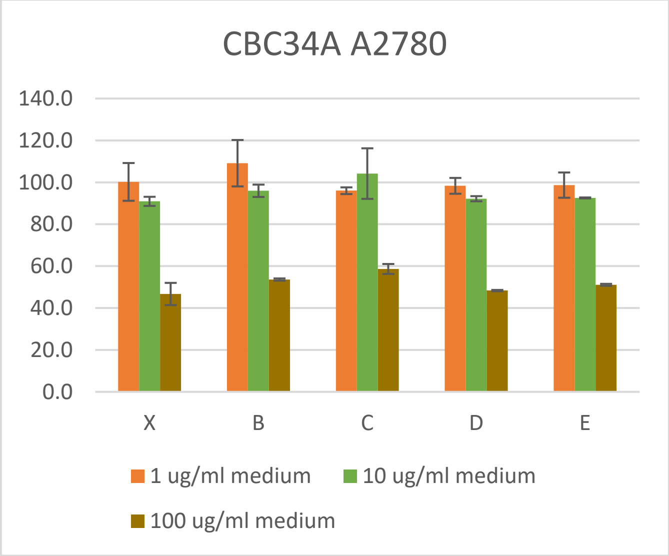

CBC 35 A: (Miozoa)

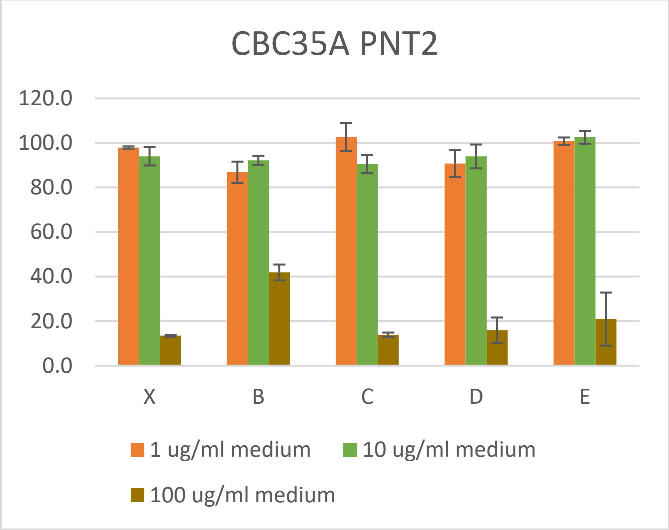

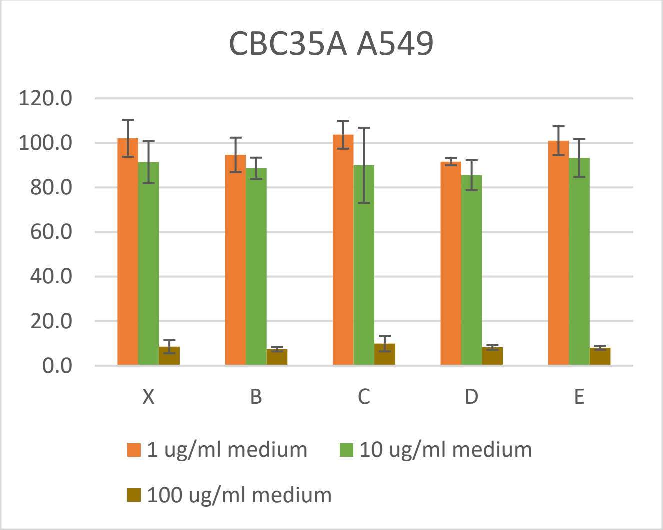

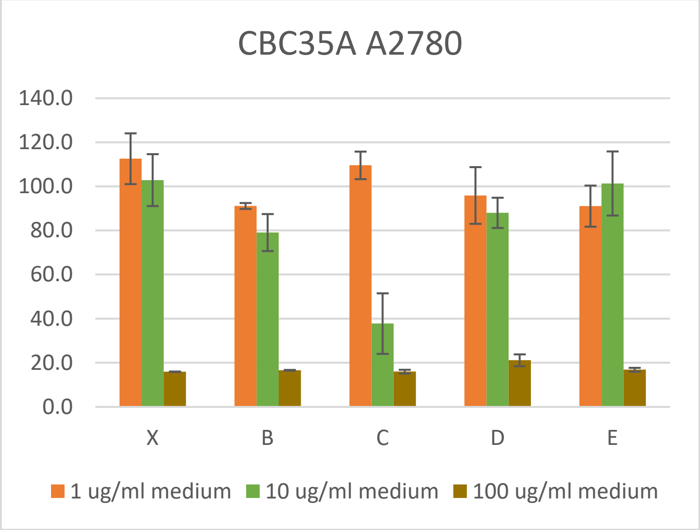

CBC 36 A: (Bacillariophyta)

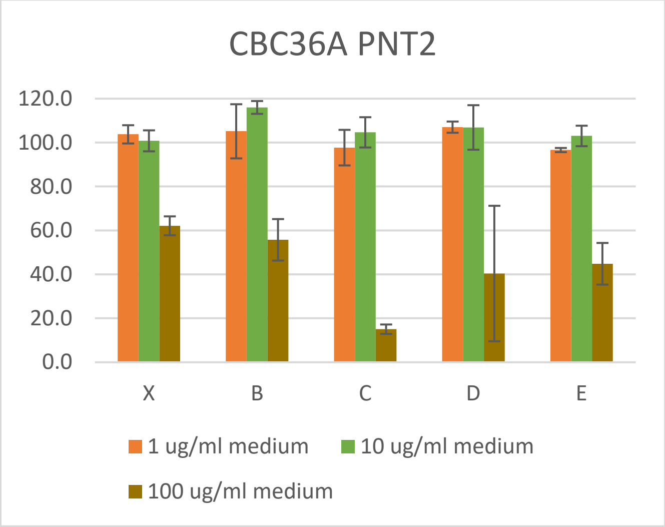

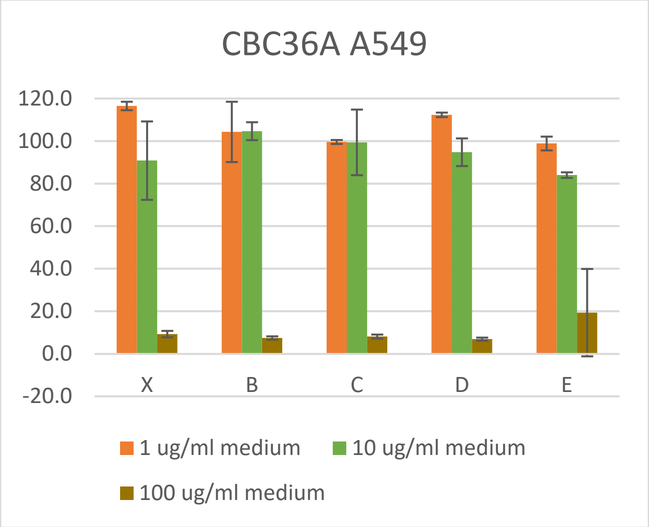

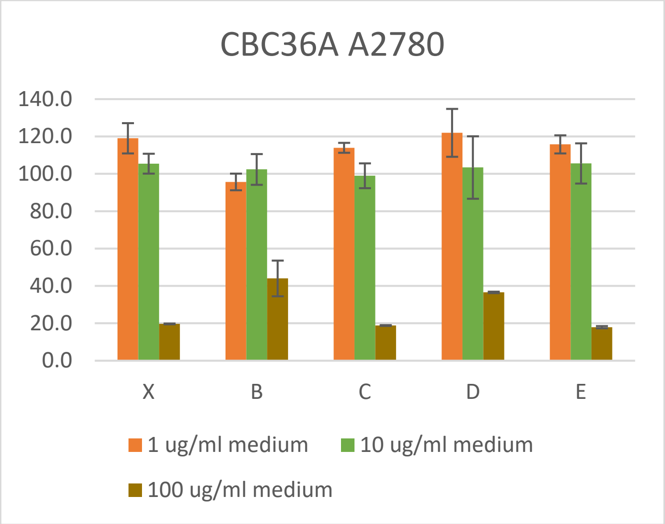

CBC 37 A: (Chordata)

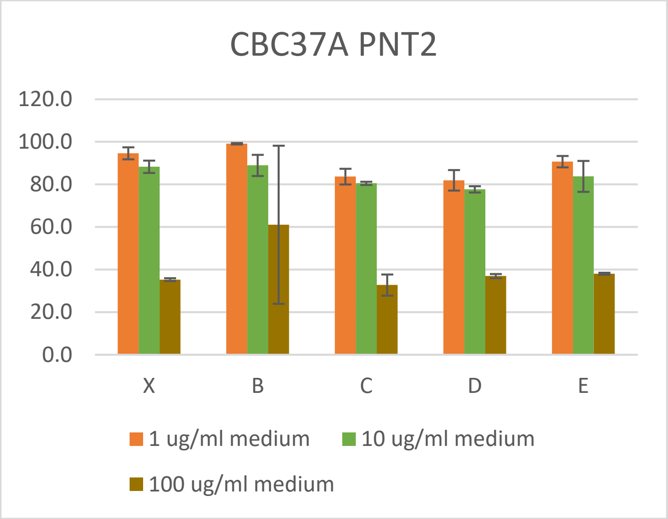

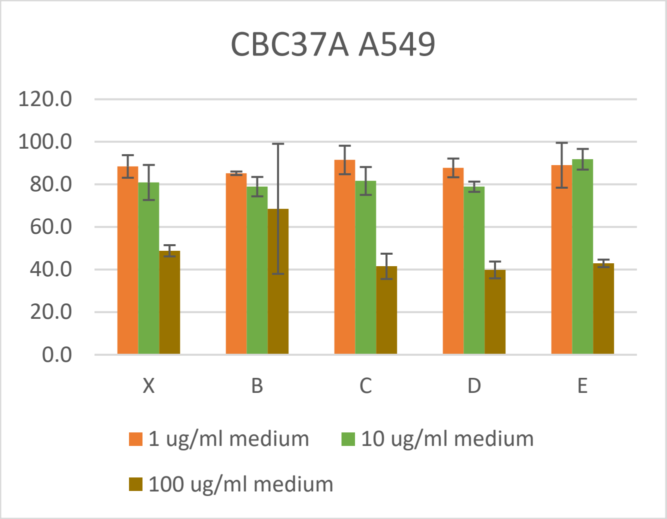

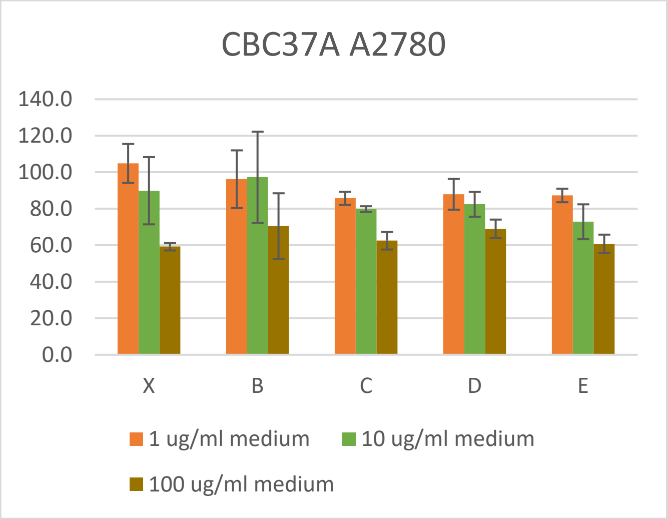

CBC 38 A: (Mollusca)

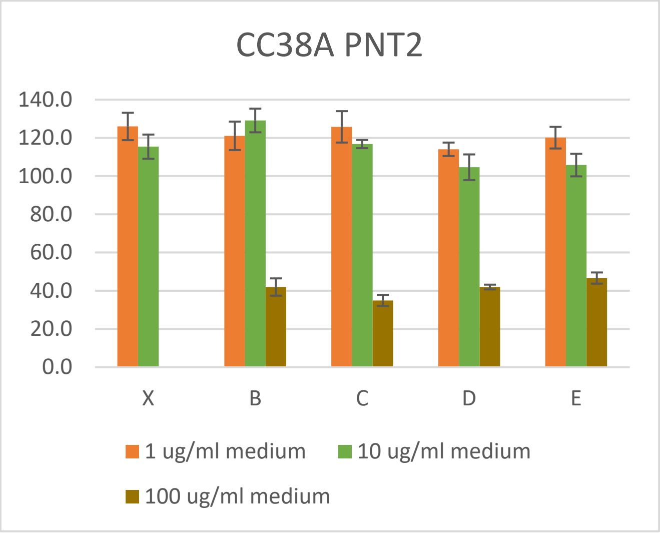

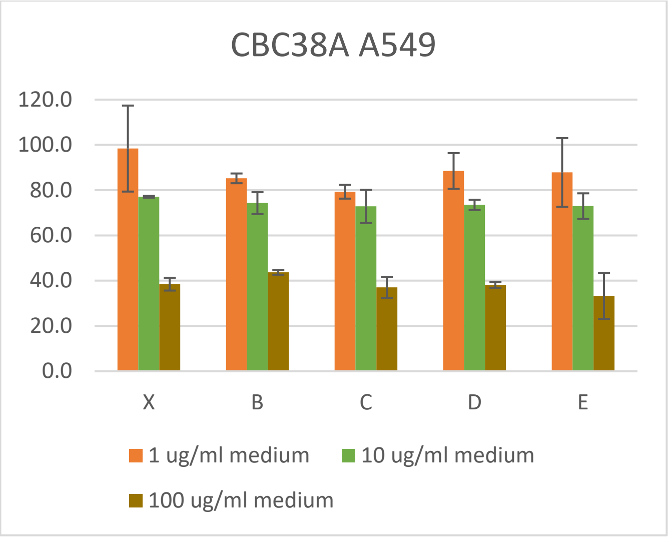

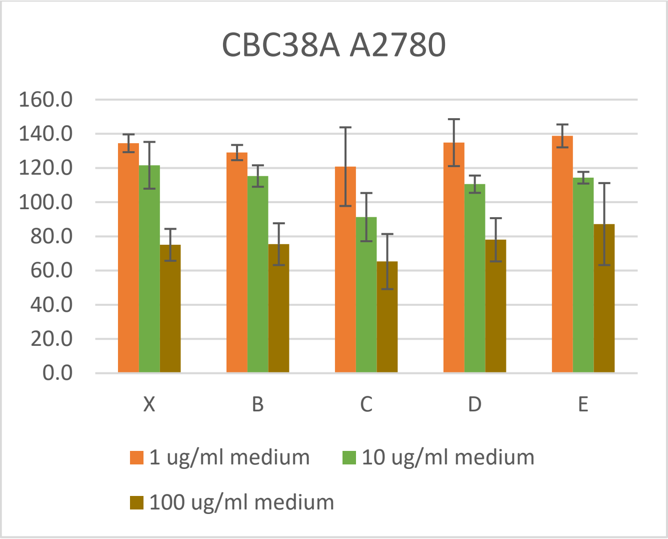

CBC 39 A: (Miozoa)

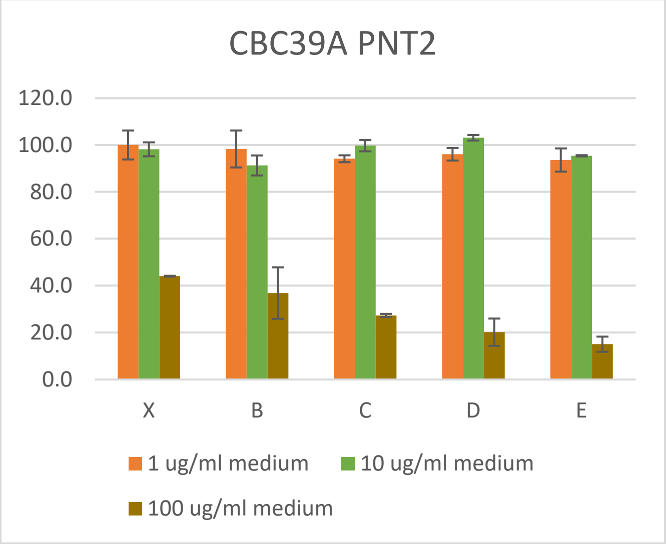

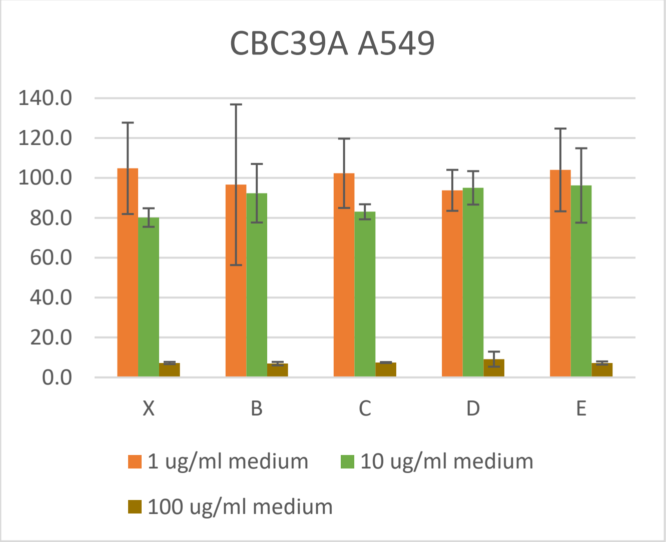

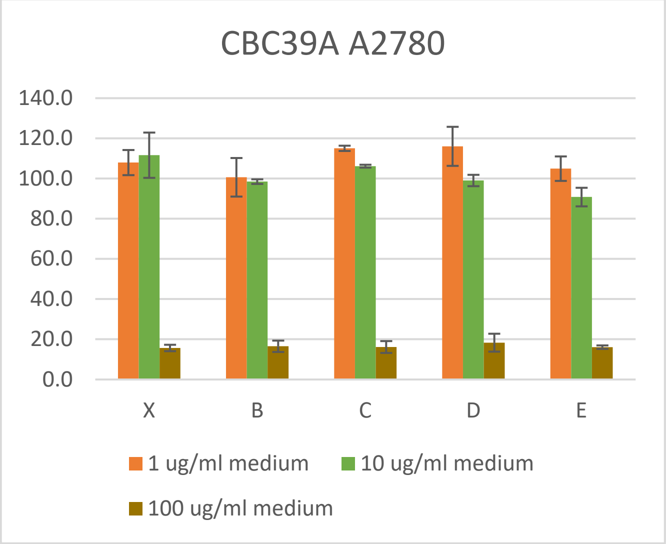

CBC 40 A: (Bacillariophyta)

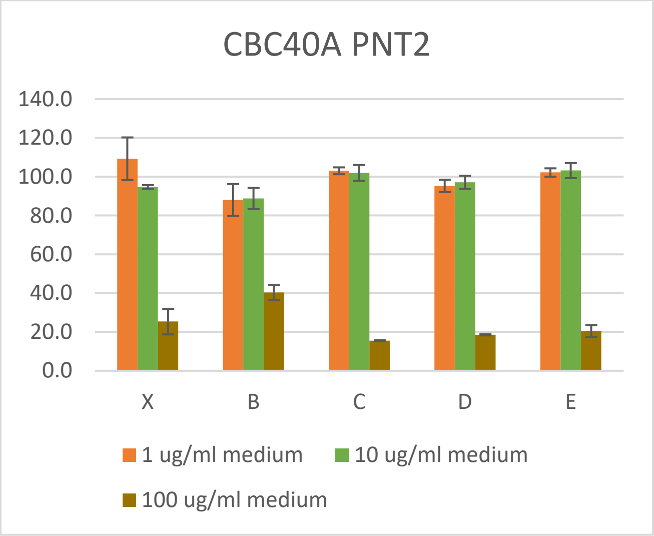

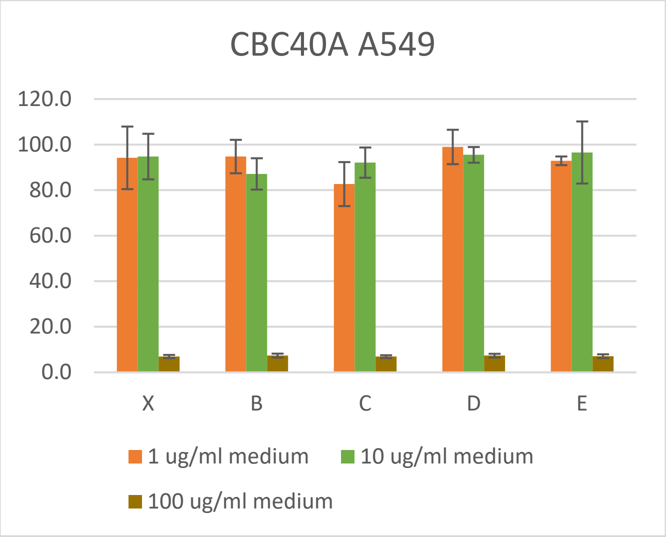

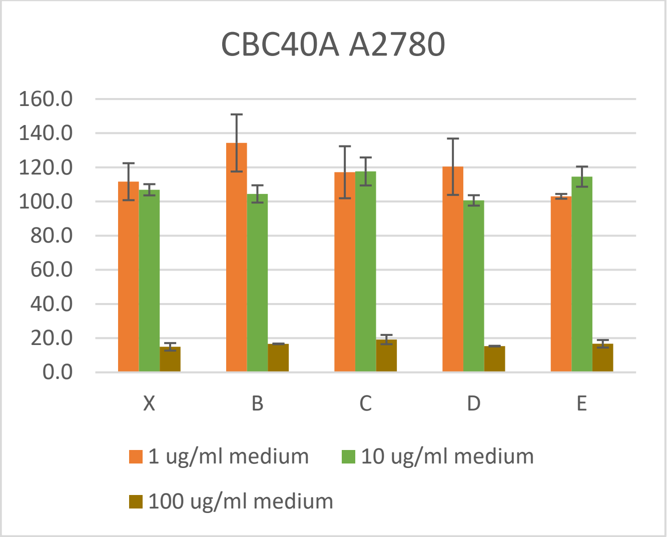

CBC 41 A: (Bacillariophyta)

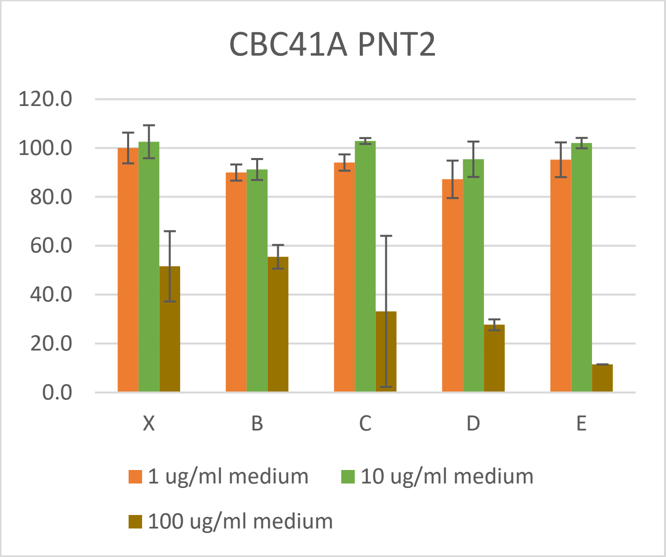

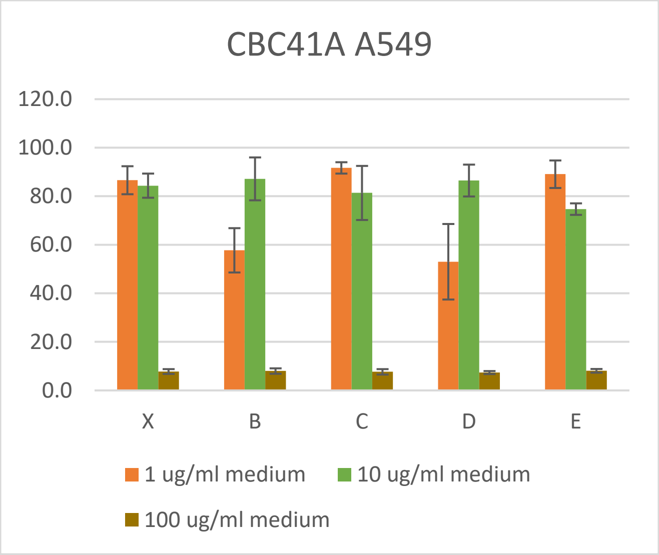

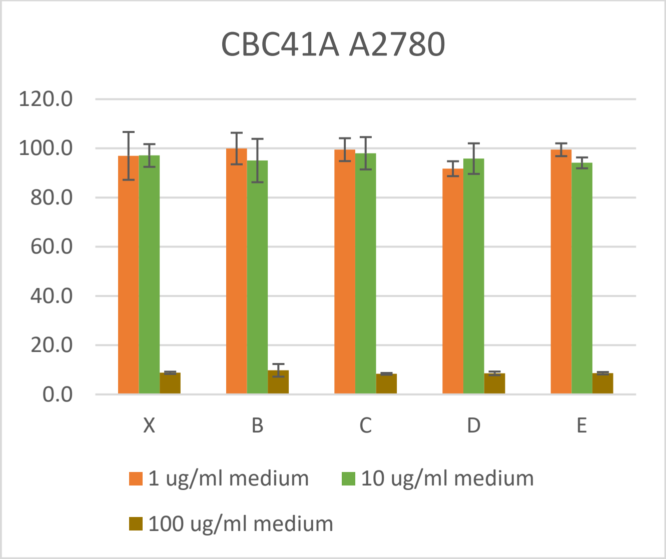

CBC 42 A: (Bacillariophyta)

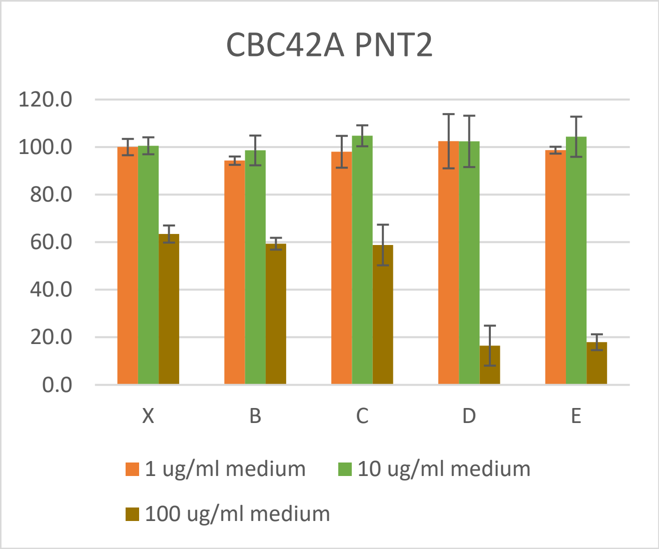

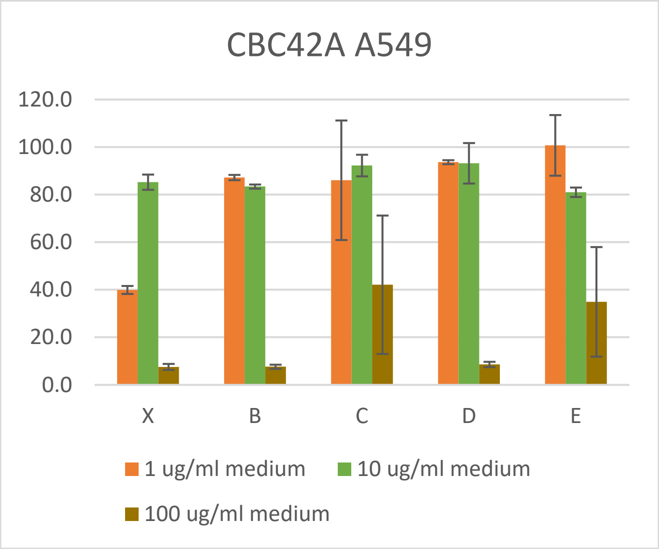

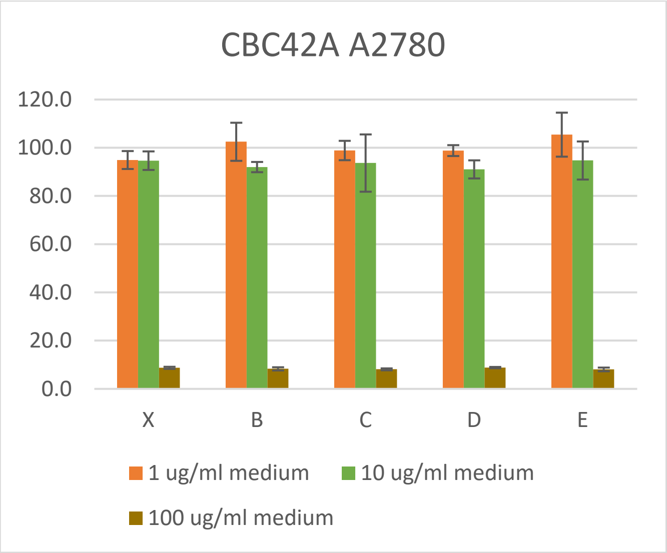

CBC 43 A: (Rhodophyta)

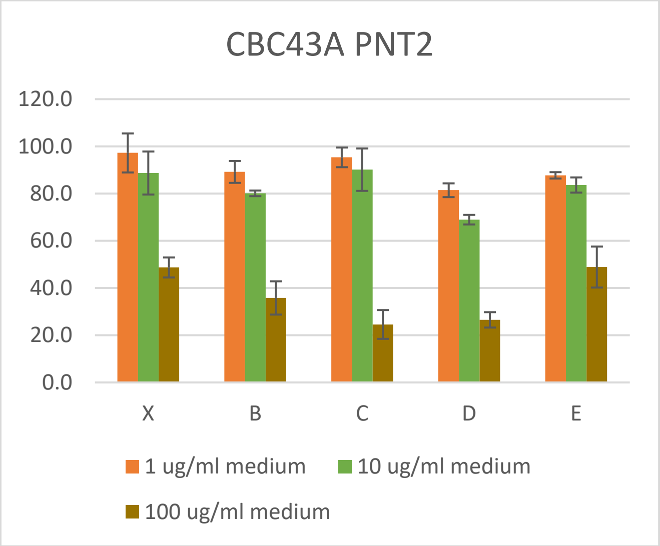

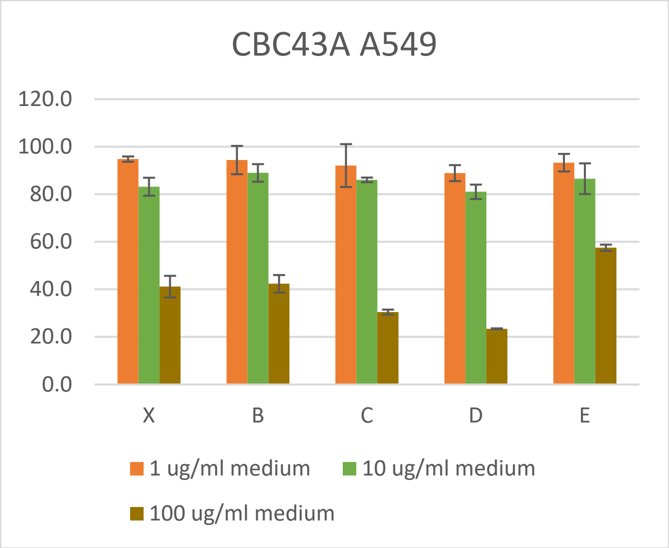

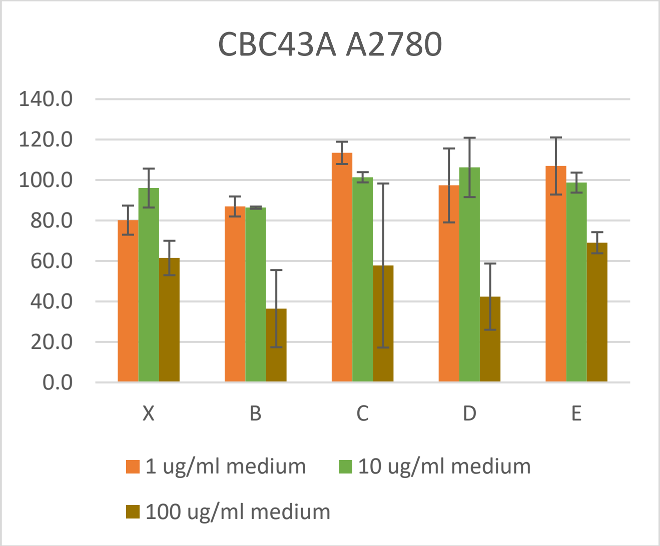

CBC 44 A: (Briozoa)

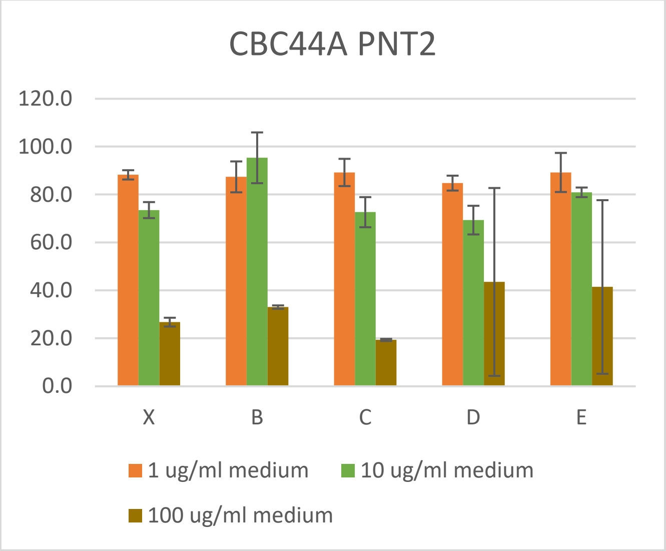

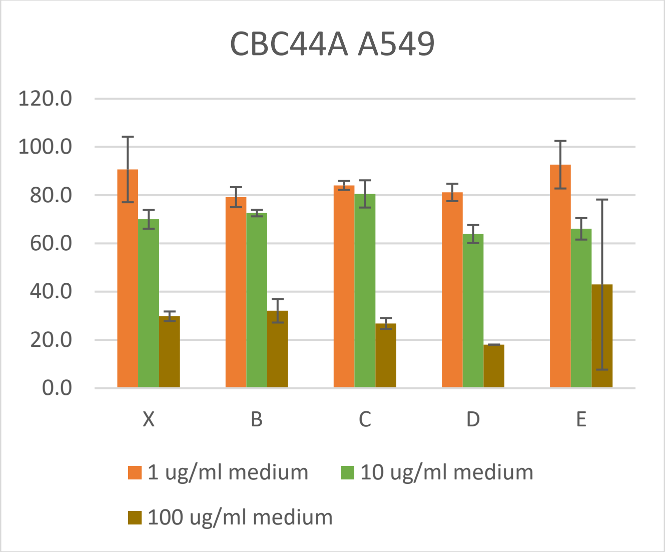

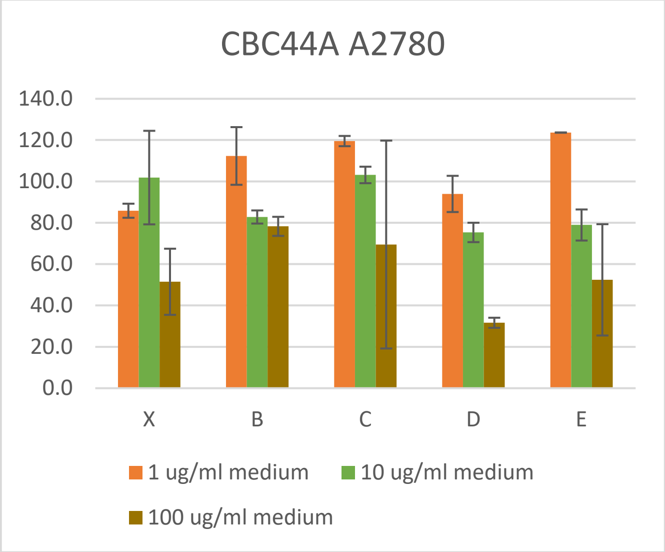

CBC 45 A: (Ochrophyta)

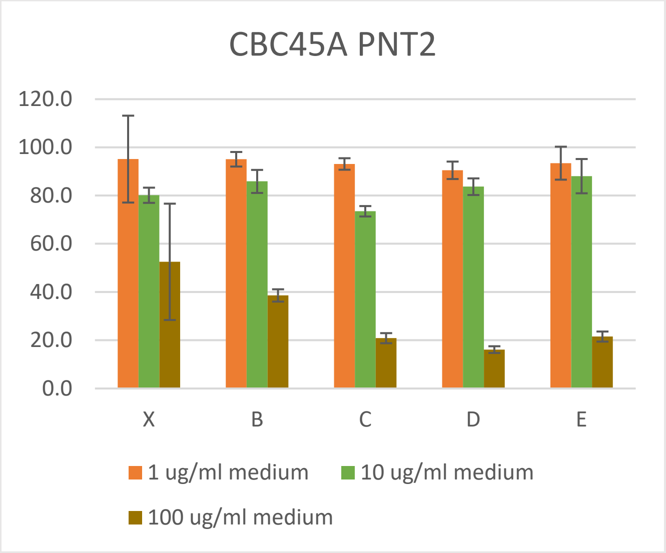

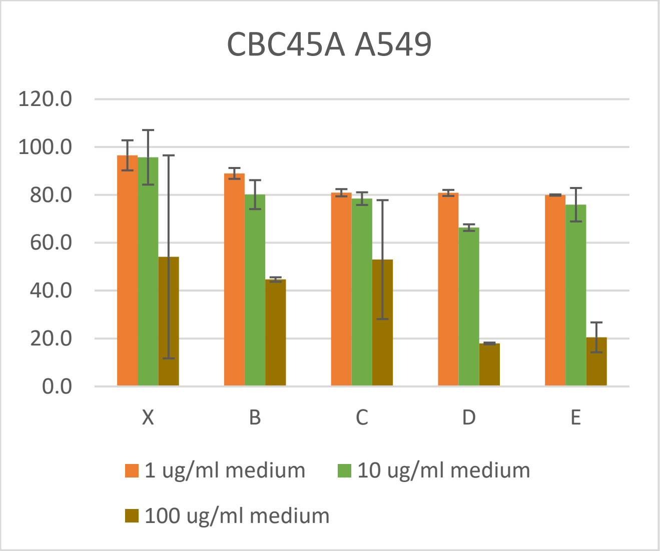

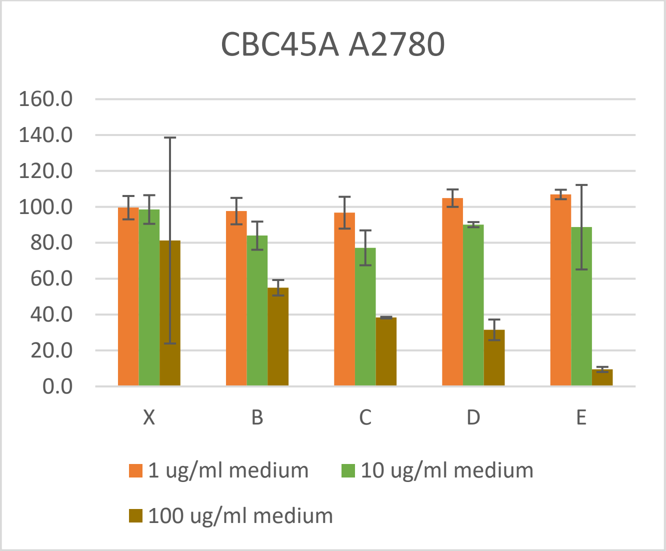

CBC 46 A: (Mollusca)

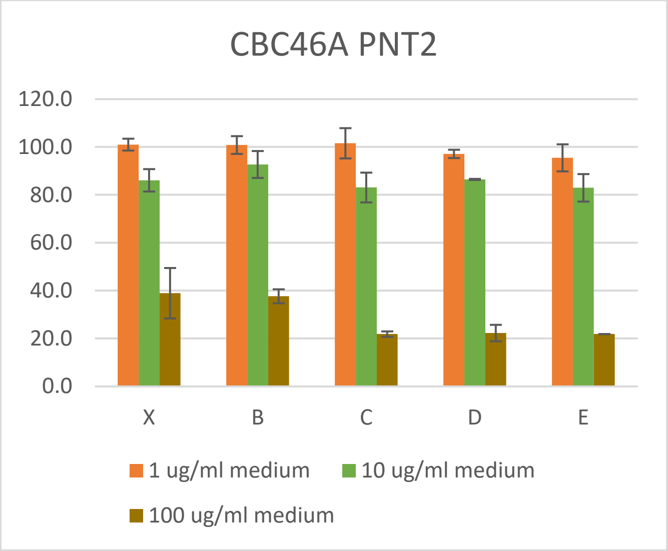

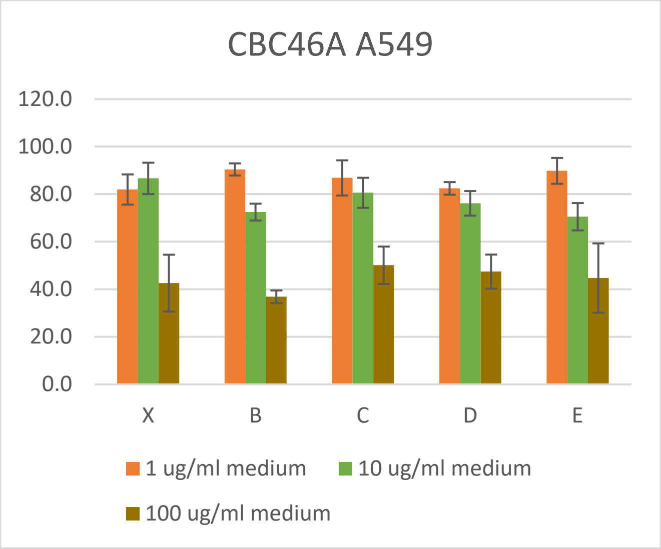

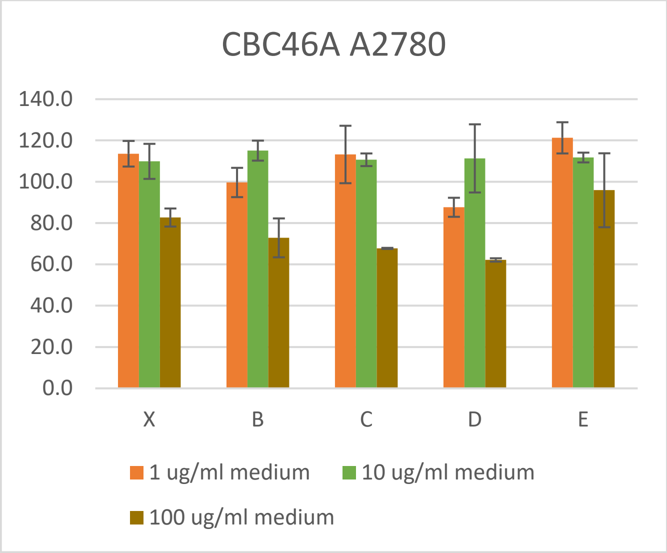

CBC 46 C: (Mollusca)

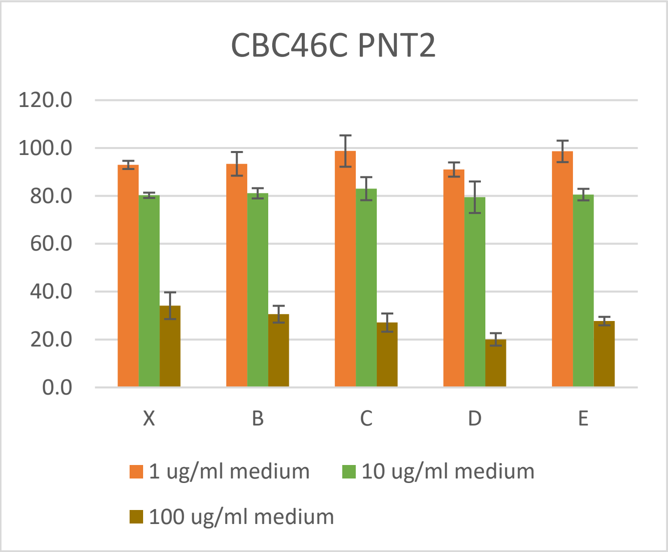

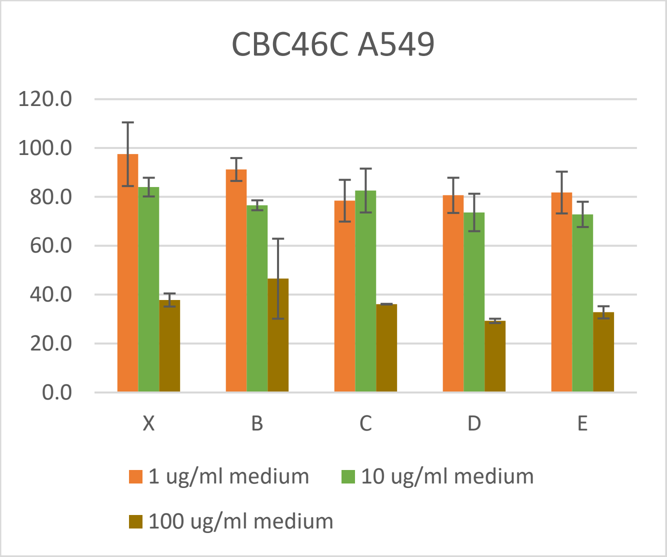

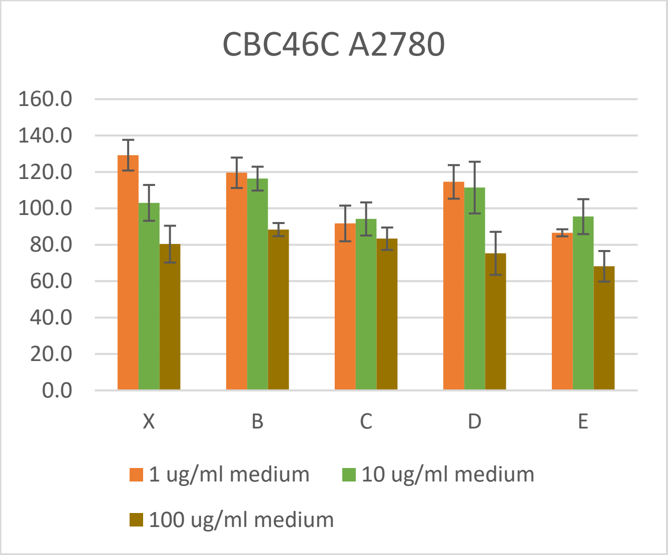

CBC 47 A: (Mollusca)

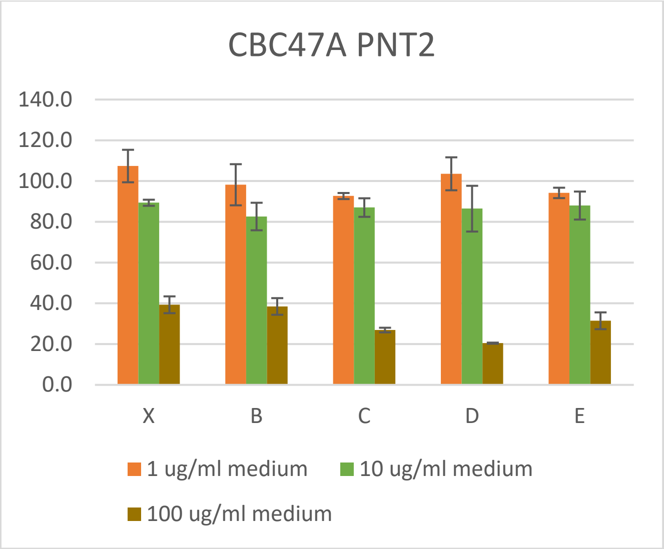

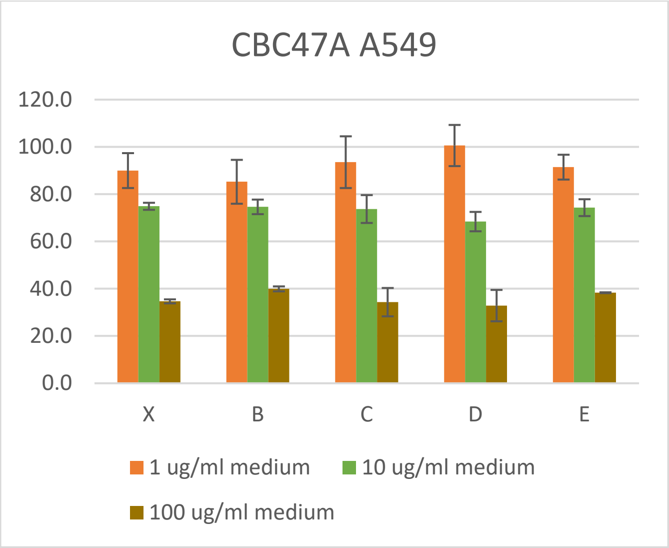

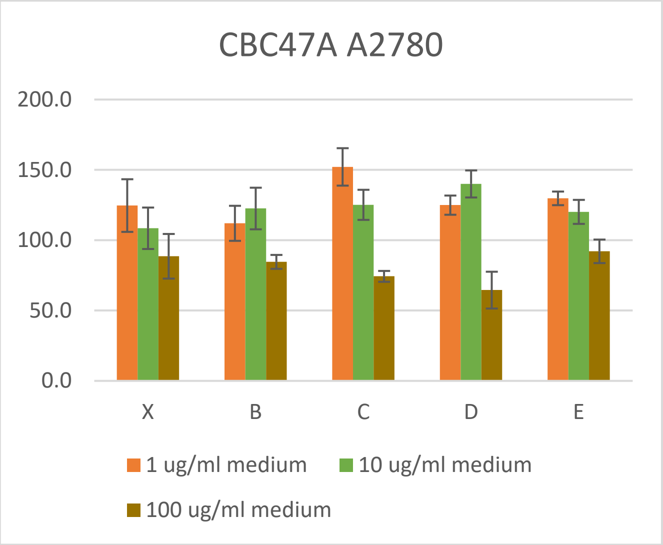

CBC 48 A: (Briozoa)

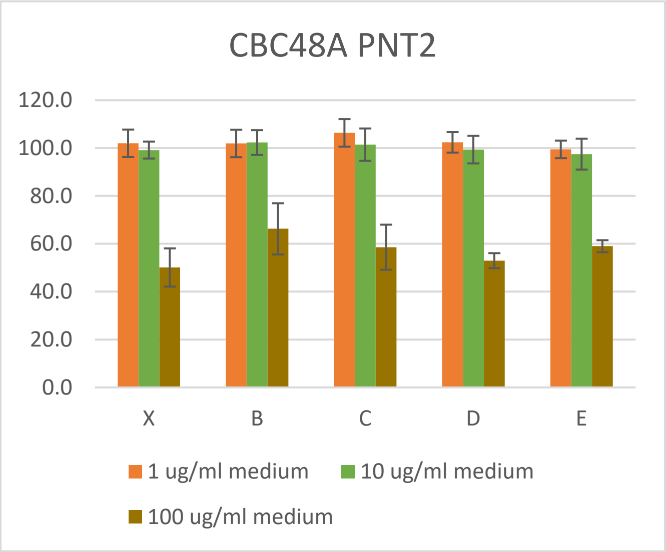

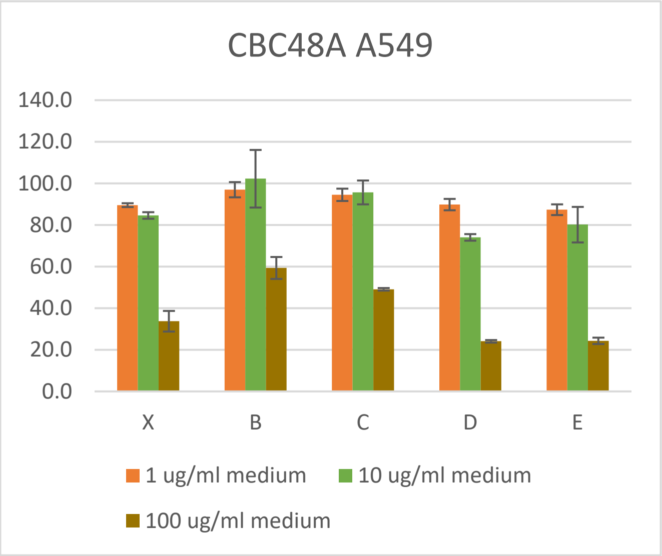

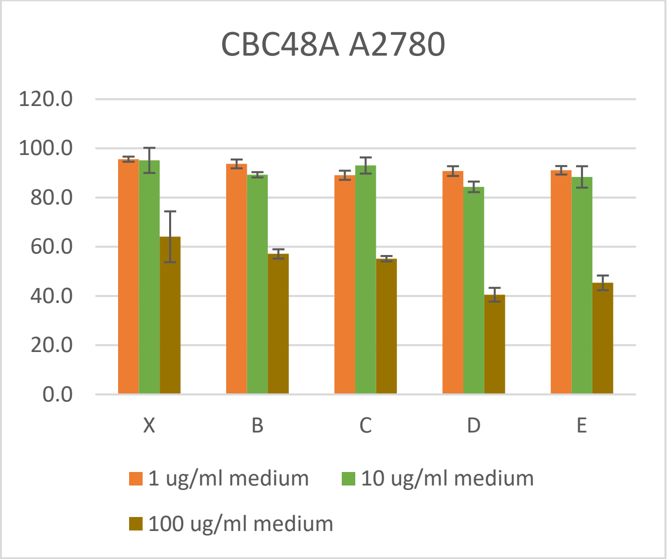

CBC 49 A: (Ochrophyta)

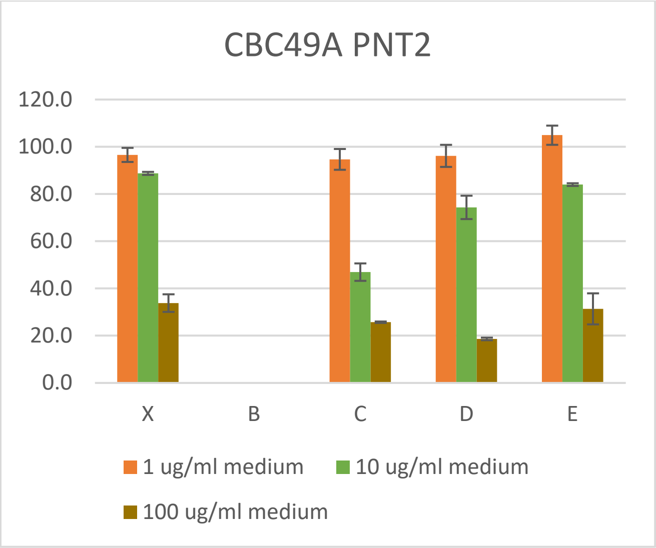

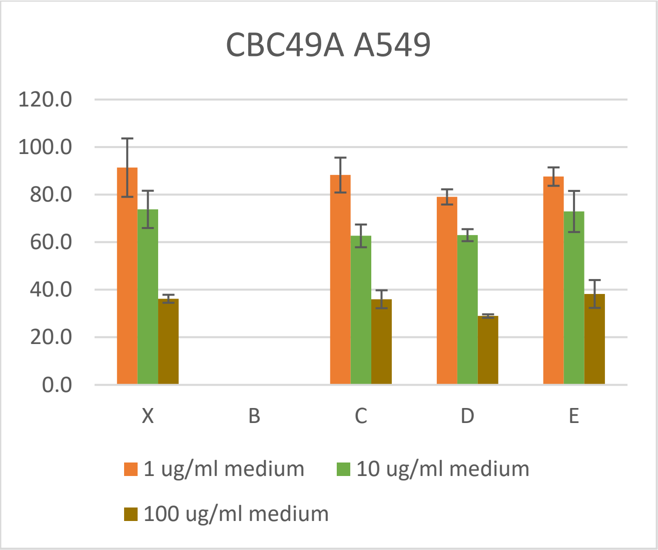

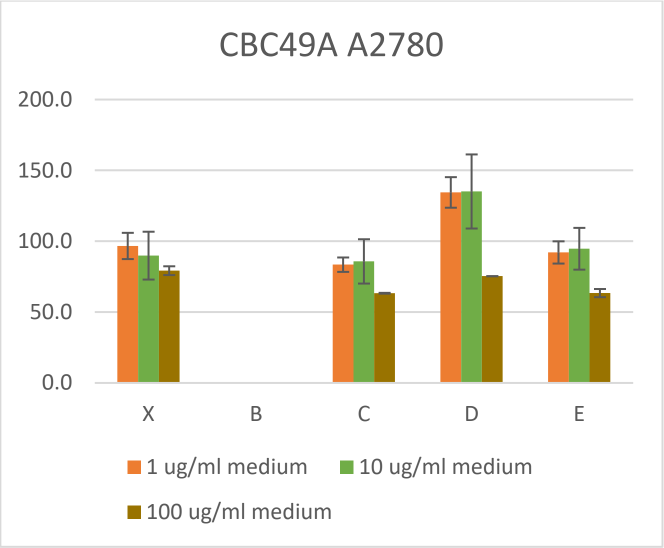

CBC 50 A: (Chordata)

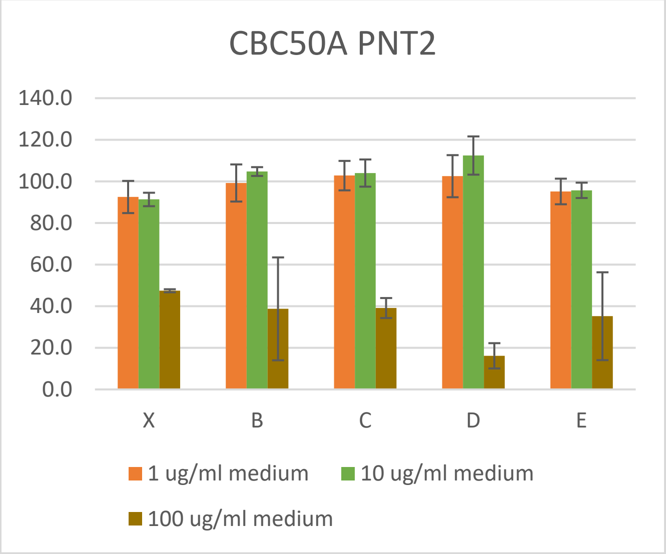

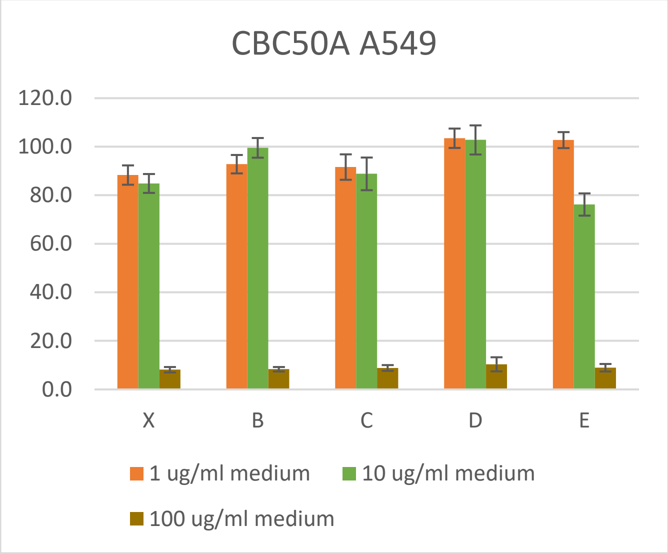

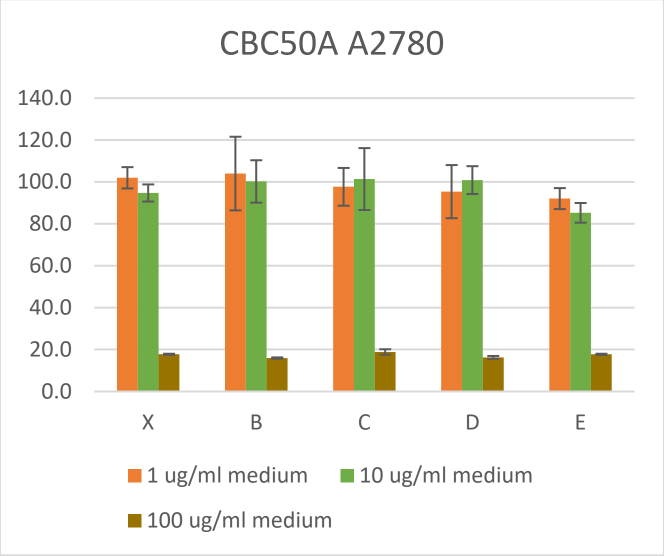

CBC 51 A: (Cnidaria)

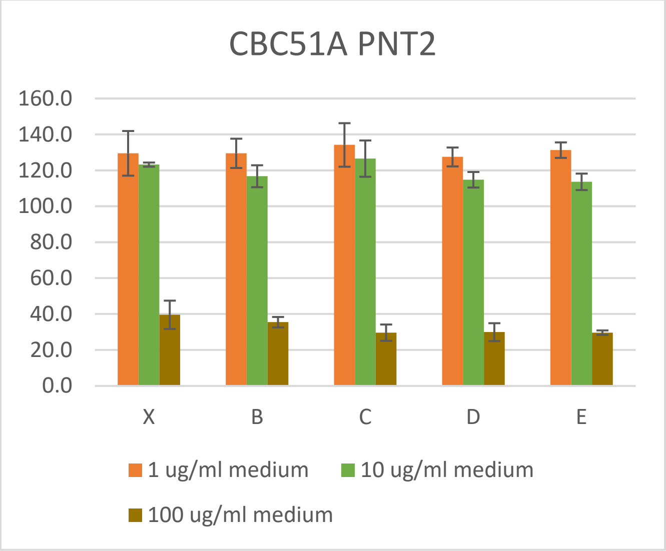

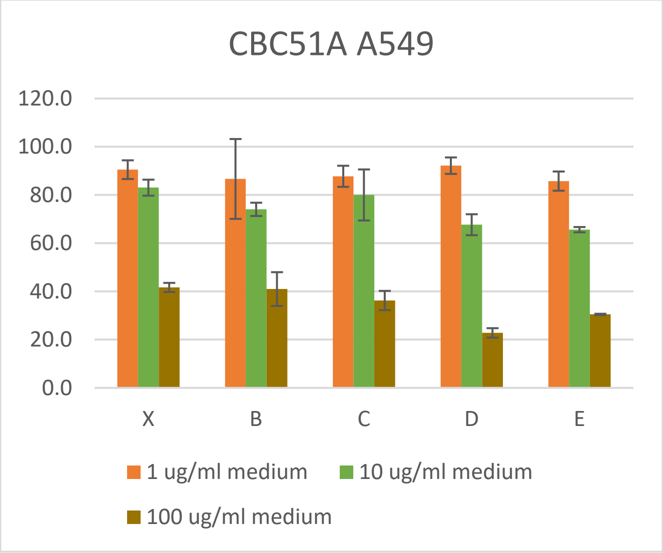

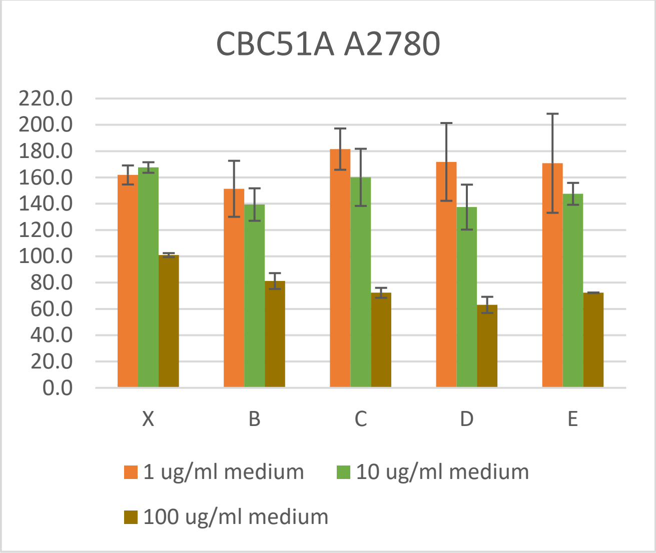

CBC 53 A: (Miozoa)

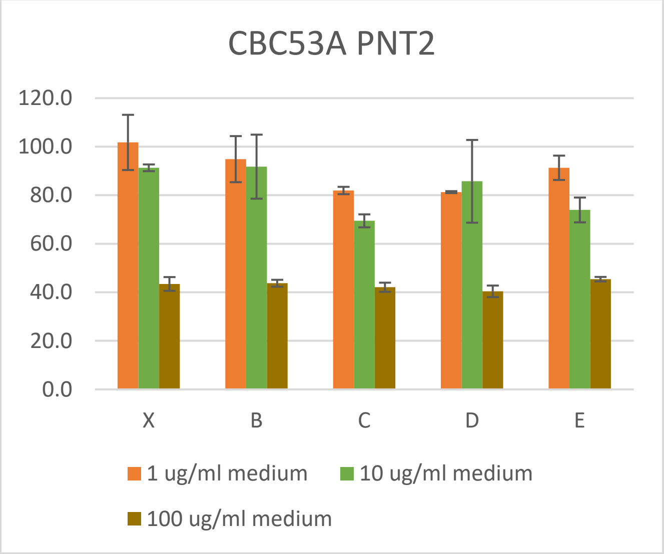

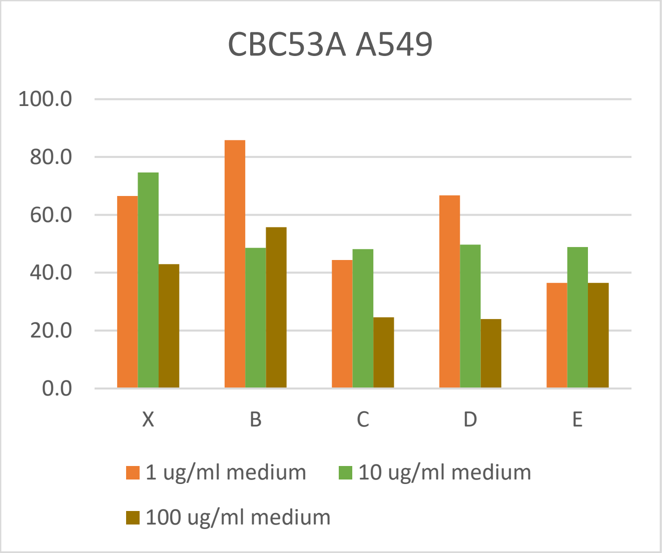

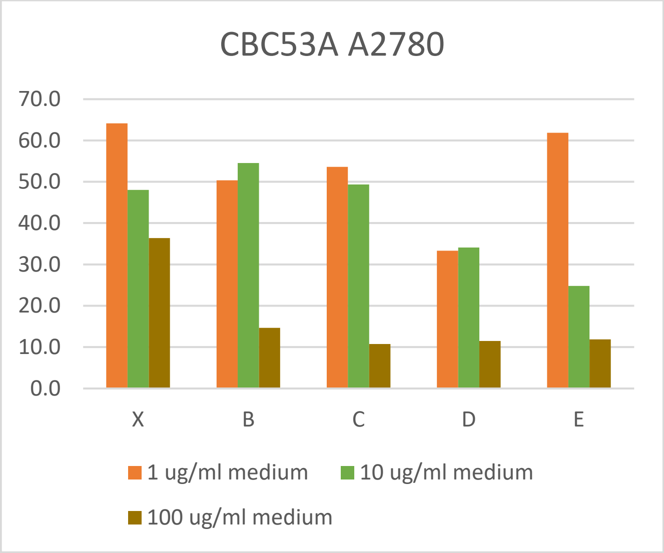

CBC 55 A: (Miozoa)

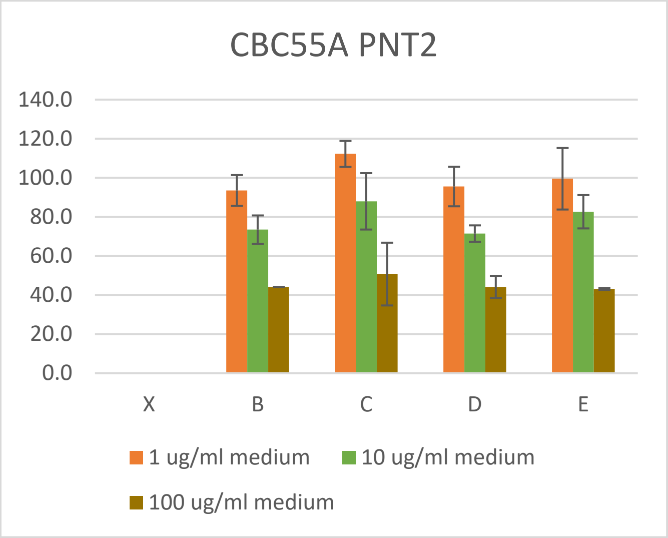

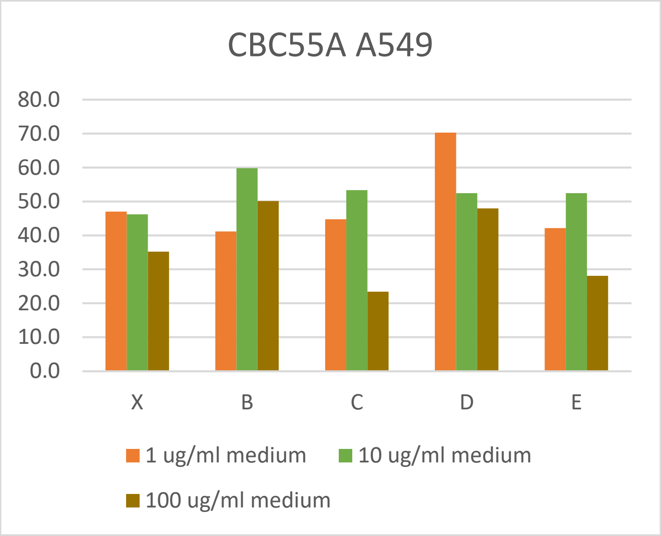

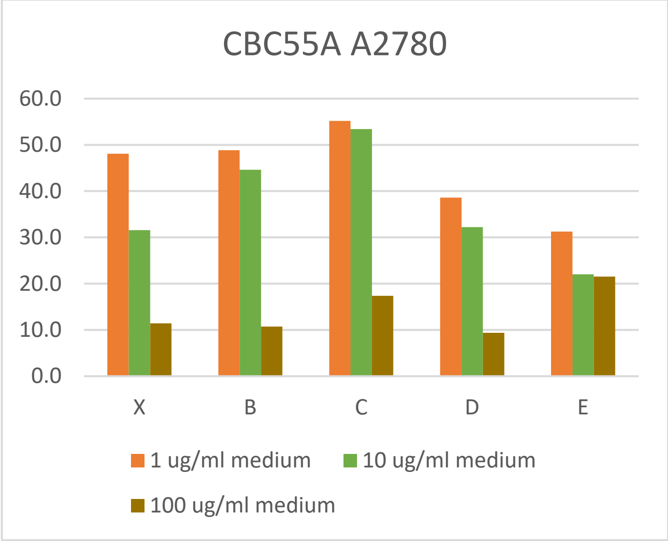

CBC 56 A: (Porifera)

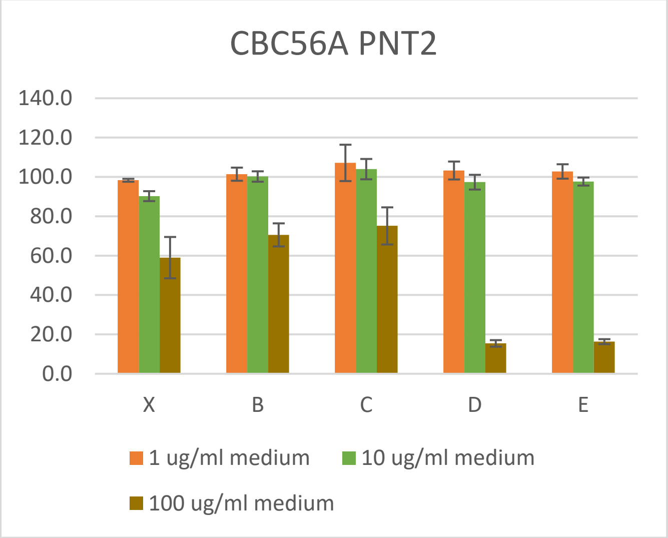

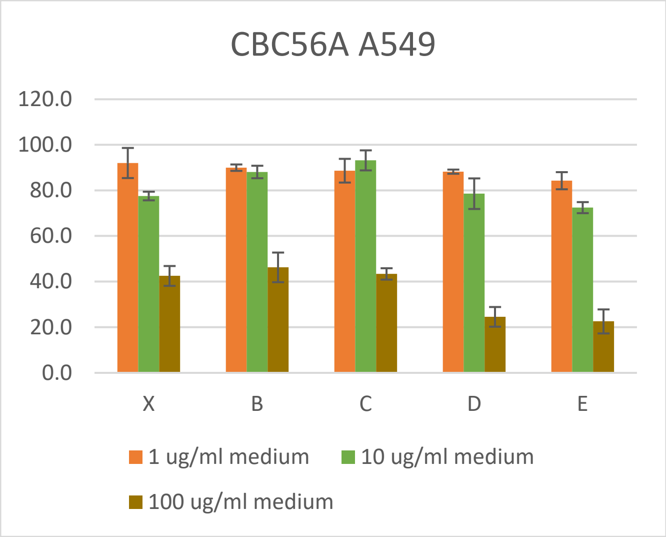

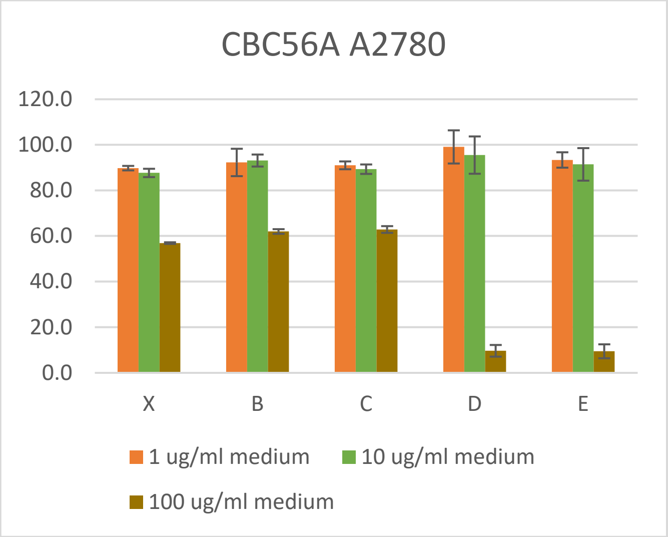

CBC 57 A: (Porifera)

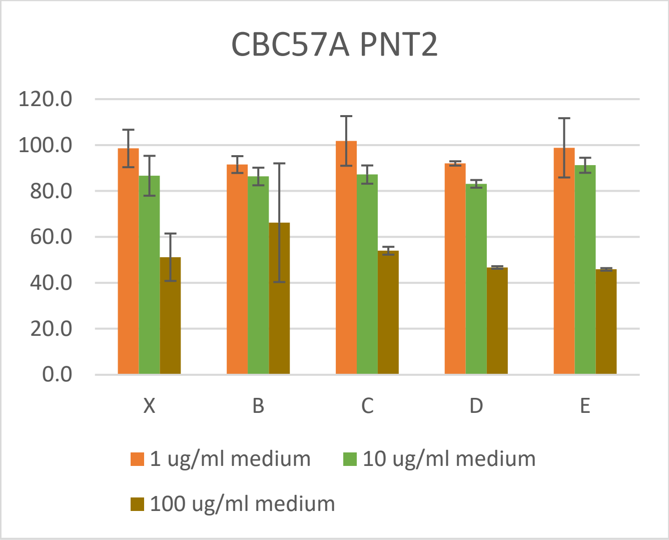

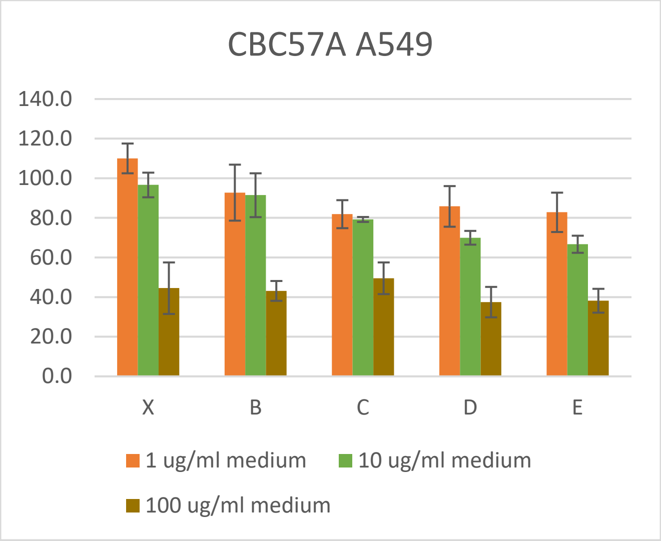

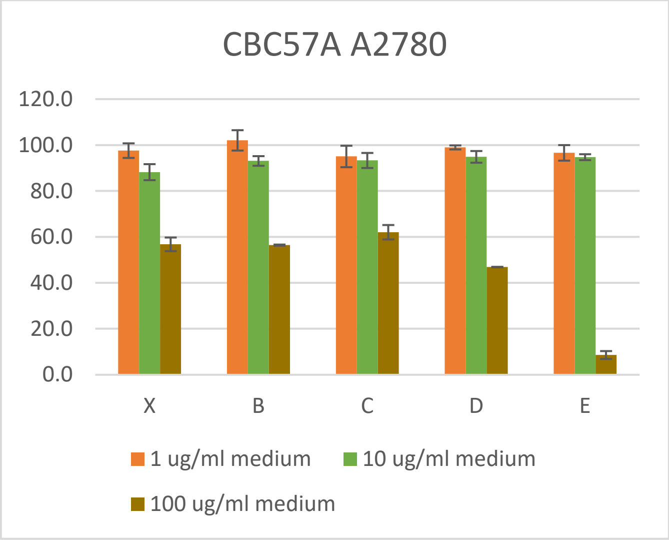

CBC 62 A: (Cnidaria)

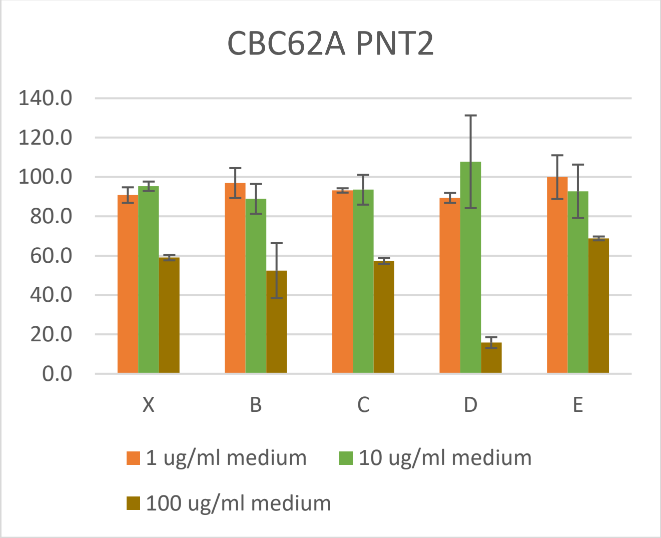

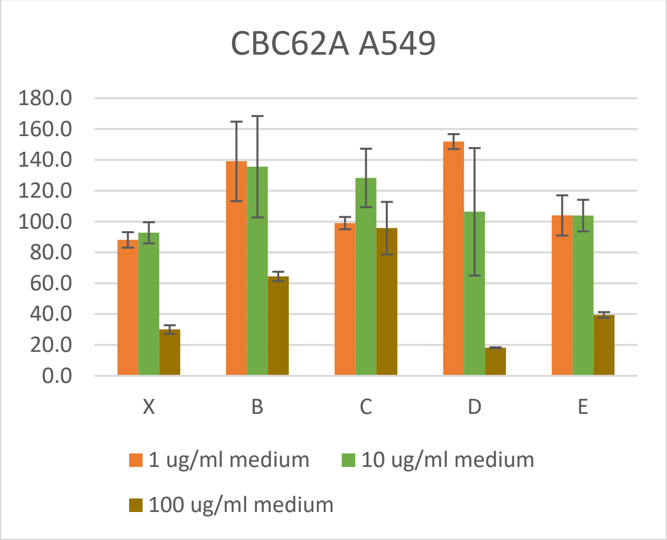

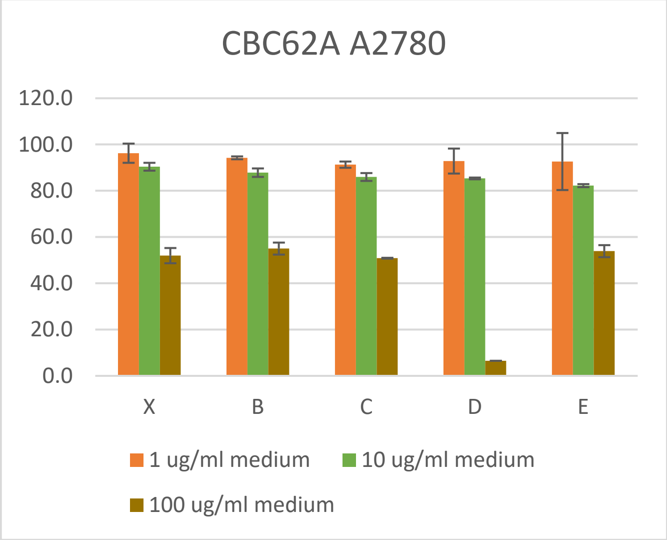

CBC 74 A (Num 1): (Porifera)

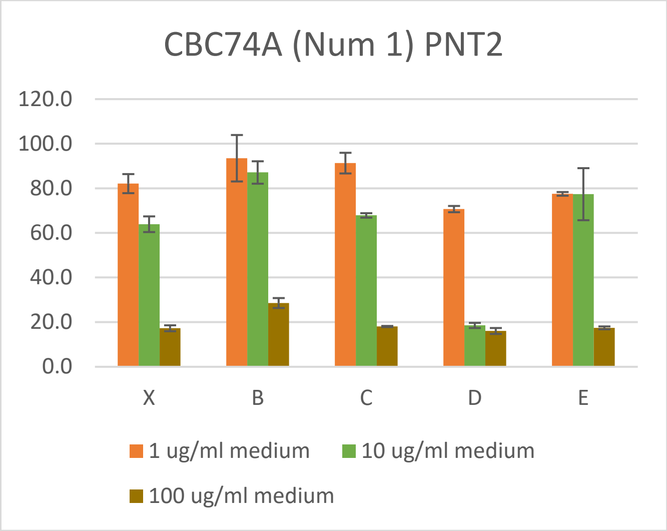

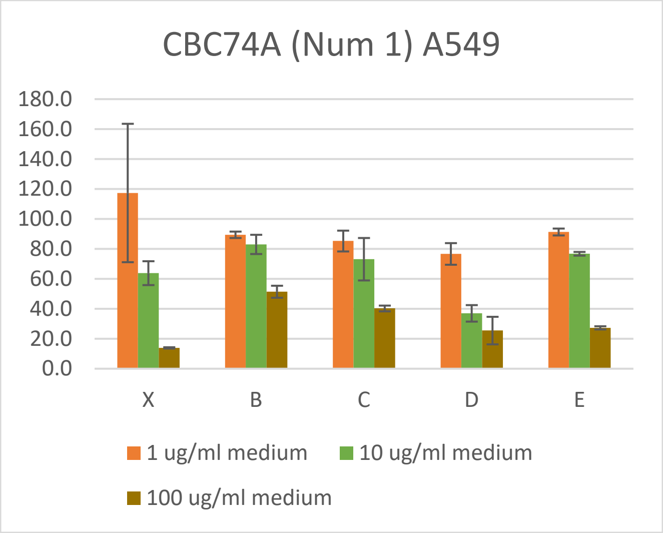

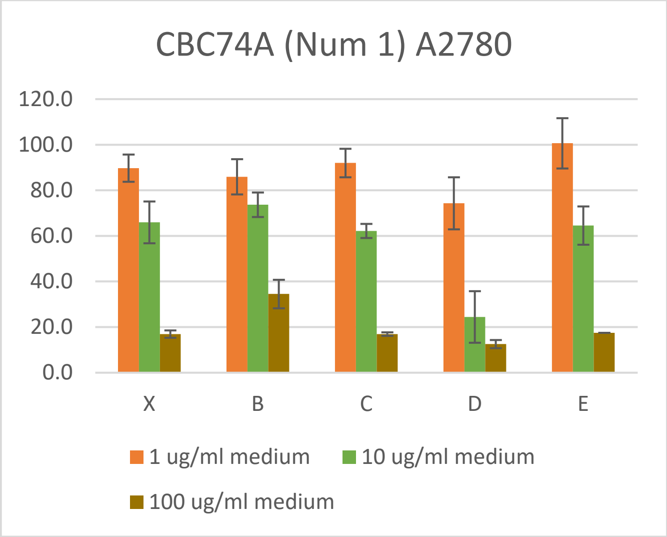

CBC 74 A (Num 3): (Porifera)

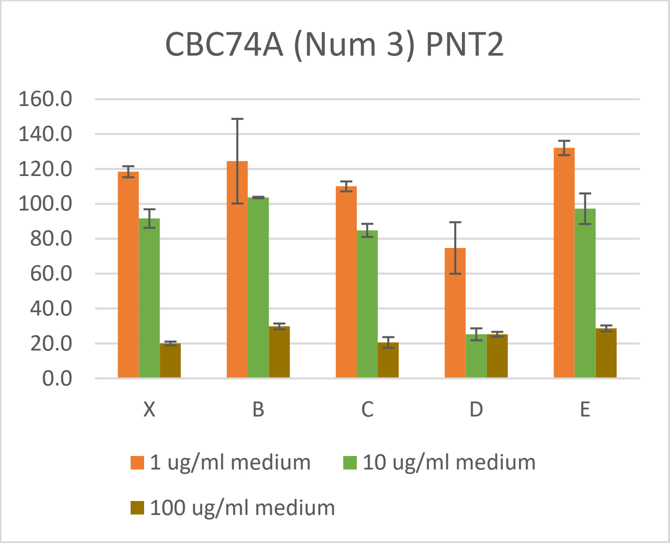

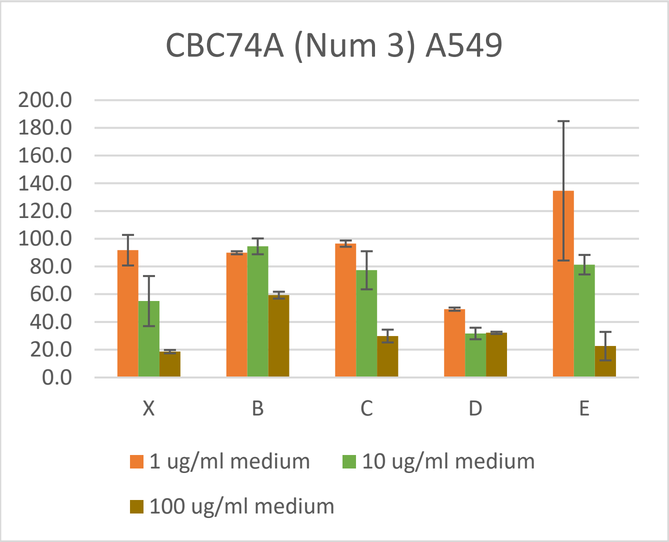

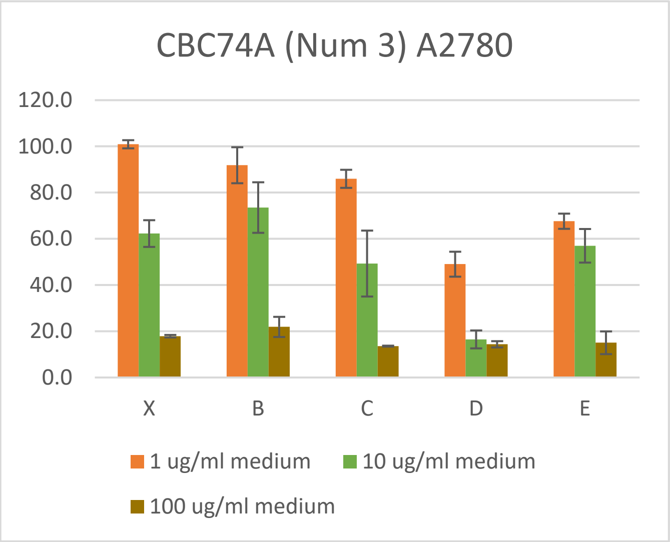

CBC 74 B: (Porifera)

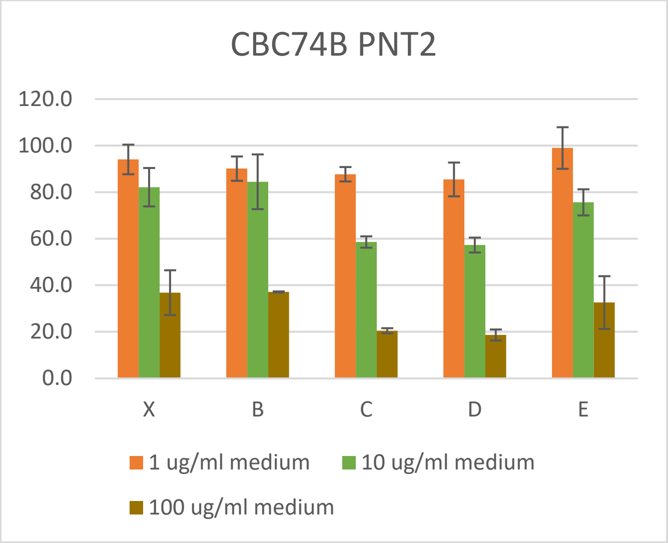

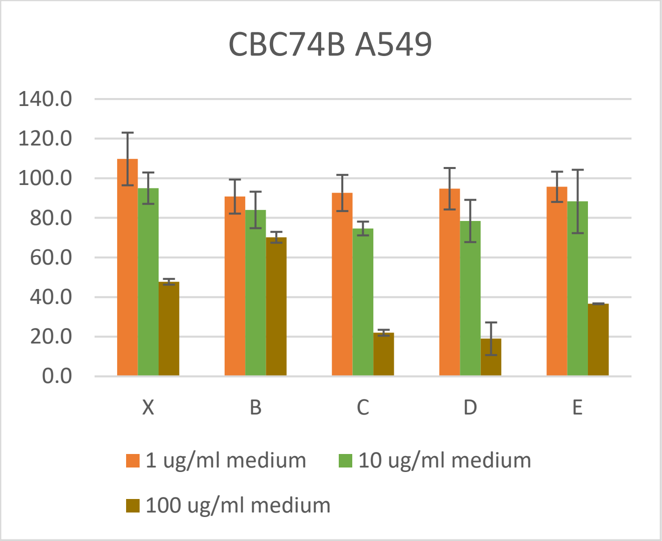

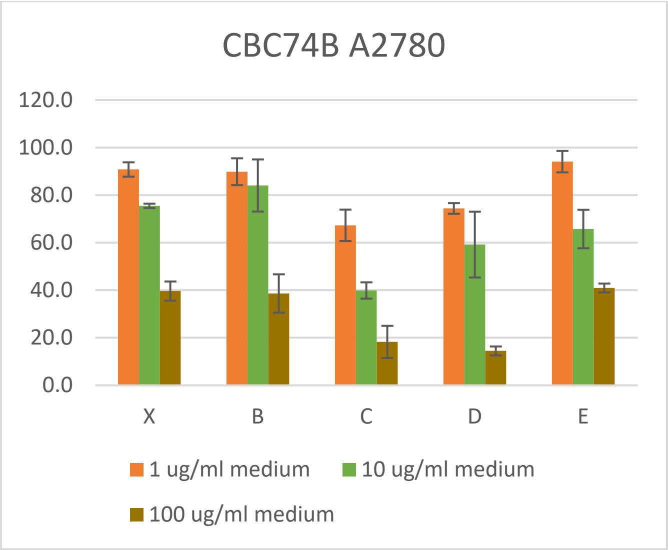

CBC 75 A: (Porifera)

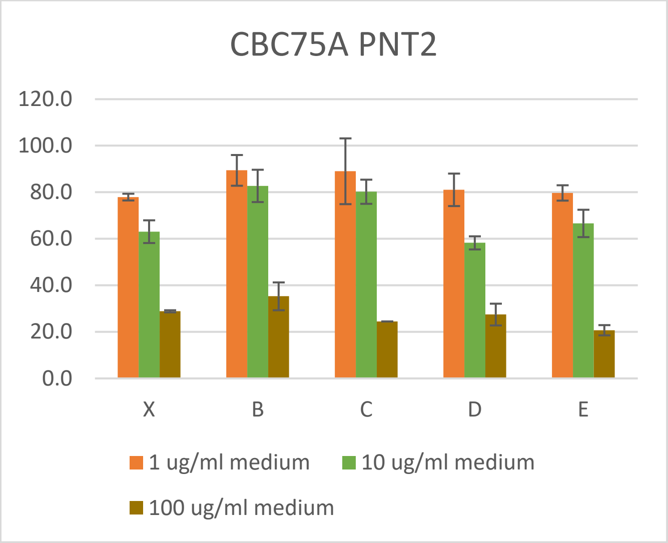

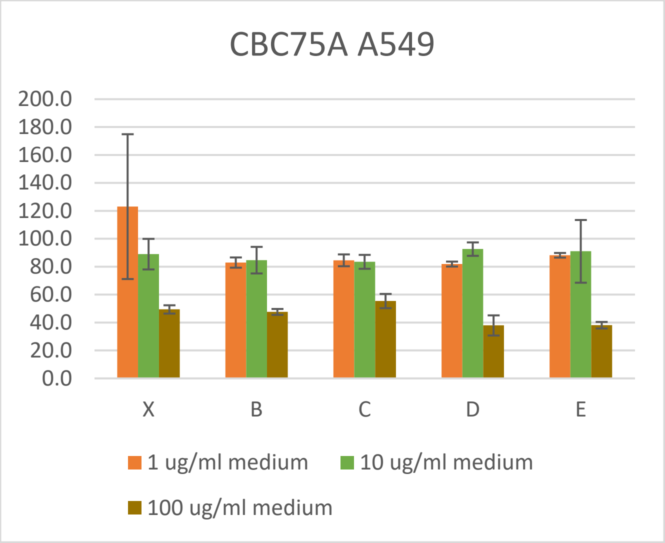

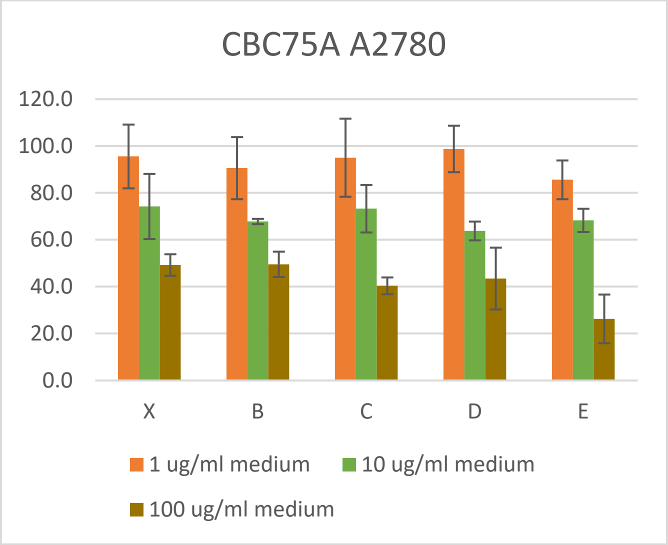

CBC 77 A: (Porifera)

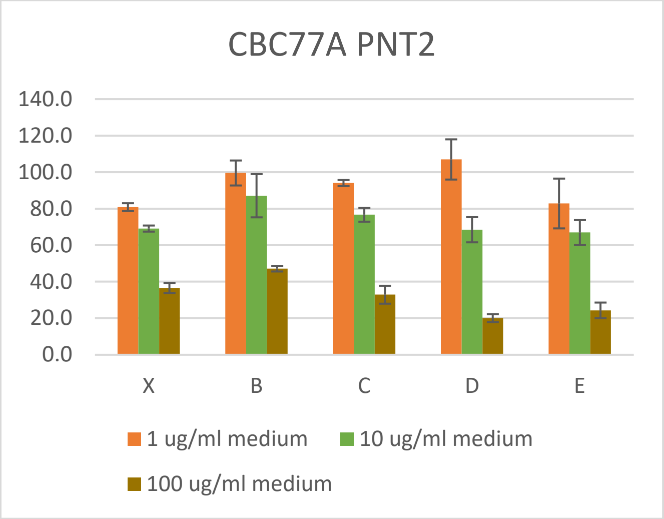

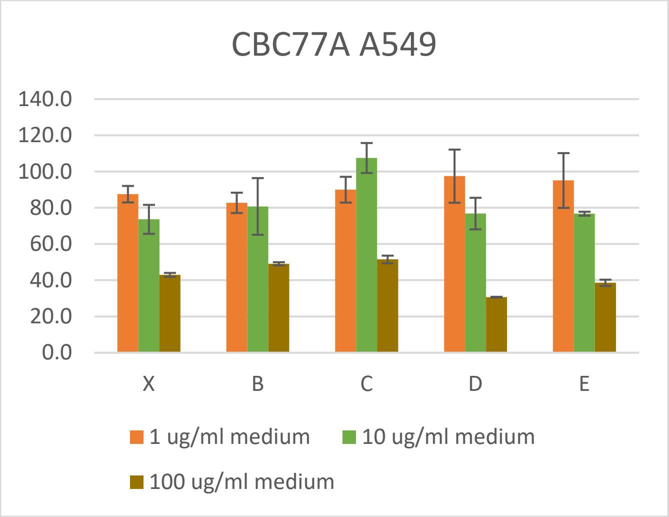

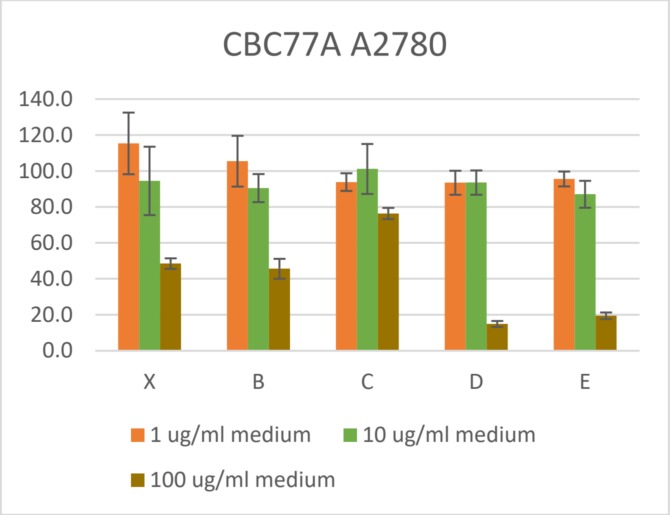

Supplement: Supplementary file 1 [file marinedrugs-19-00640-s001.zip › File S1 (cytotoxicity data).pdf]
