# Supplementary material for: Probing the Therapeutic Potential of Marine Phyla by SPE Extraction
Source: Mar Drugs. 2021 Nov 16;19(11):640. doi: 10.3390/md19110640 (PMC8625500; doi:10.3390/md19110640)
Supplement: Supplementary file 1 [file marinedrugs-19-00640-s001.zip › File S2 (antibacterial data).pdf]

Antibacteric assay of the in-house library of Marine Natural Products on five strains on three Gram + (*Staphylococcus aureus*; *Enterococcus faecalis* and *Streptococcus agalactiae* group B) and two Gram – (*Escherichia coli* and *Pseudomonas aeruginosa*). The bacterial inocula were treated with 50 µg/mL of either extracts (X) or its related SPE fractions (B-E). Results were measured as OD at 600 nm after 24 h of incubation. When the OD average was under 0.05, a value comparable to those obtained in the negative controls (not inoculated), the sample was considered active (A); when the OD average was between 0.05 and under 0,09, the sample was considered weakly active (WA); over these values, was considered non active (-).

	**Gram +**			**Gram -**	
**Identification code**	***S. aureus***	***E. faecalis***	***S. agalactiae***	***E. coli***	***P. aeruginosa***
**CBC 1 A - X**	-	-	-	-	-
**CBC 1 A – B**	-	-	-	-	-
**CBC 1 A – C**	-	-	-	-	-
**CBC 1 A – D**	-	-	**A**	-	-
**CBC 1 A - E**	-	-	-	-	-
**CBC 2 A - X**	-	-	-	-	-
**CBC 2 A – B**	-	-	-	-	-
**CBC 2 A – C**	-	-	-	-	-
**CBC 2 A – D**	-	-	**A**	-	-
**CBC 2 A - E**	-	-	-	-	-
**CBC 3 A - X**	**A**	**A**	**A**	**A**	**A**
**CBC 3 A – B**	**A**	**WA**	**WA**	**A**	**A**
**CBC 3 A – C**	**A**	**WA**	**WA**	**A**	**A**
**CBC 3 A – D**	-	-	-	-	-
**CBC 3 A - E**	-	-	-	-	-
**CBC 4 A - X**	-	-	-	-	-

**CBC 4 A – B**	-	-	-	-	-
**CBC 4 A – C**	-	-	-	-	-
**CBC 4 A – D**	-	-	-	-	-
**CBC 4 A - E**	-	-	-	-	-
**CBC 10 A - X**	-	-	-	-	-
**CBC 10 A – B**	-	-	-	-	-
**CBC 10 A – C**	-	-	-	-	-
**CBC 10 A – D**	-	-	-	-	-
**CBC 10 A - E**	-	-	-	-	-
**CBC 11 A - X**	-	-	-	-	-
**CBC 11 A – B**	-	-	-	-	-
**CBC 11 A – C**	-	-	-	-	-
**CBC 11 A – D**	-	-	**A**	-	-
**CBC 11 A - E**	-	-	-	-	-
**CBC 12 A - X**	**A**	**A**	**A**	-	-
**CBC 12 A – B**	-	-	-	-	-
**CBC 12 A – C**	-	-	-	-	-
**CBC 12 A – D**	-	**A**	**A**	-	-
**CBC 12 A - E**	**A**	**A**	**A**	-	-
**CBC 13 A - X**	-	-	-	-	-
**CBC 13 A – B**	-	-	-	-	-
**CBC 13 A – C**	-	-	-	-	-
**CBC 13 A – D**	-	-	-	-	-
**CBC 13 A - E**	-	-	**A**	-	-
**CBC 14 A - X**	-	-	-	-	-
**CBC 14 A – B**	-	-	-	-	-
**CBC 14 A – C**	-	-	-	-	-

**CBC 14 A – D**	**-**	**-**	**-**	**-**	**-**
**CBC 14 A - E**	**A**	**-**	**A**	**-**	**-**
**CBC 15 A - X**	**-**	**-**	**-**	**-**	**-**
**CBC 15 A – B**	**-**	**-**	**-**	**-**	**-**
**CBC 15 A – C**	**-**	**-**	**-**	**-**	**-**
**CBC 15 A – D**	**-**	**-**	**-**	**-**	**-**
**CBC 15 A - E**	**-**	**-**	**-**	**-**	**-**
**CBC 16 A - X**	**-**	**-**	**-**	**-**	**-**
**CBC 16 A – B**	**-**	**-**	**-**	**-**	**-**
**CBC 16 A – C**	**-**	**-**	**-**	**-**	**-**
**CBC 16 A – D**	**-**	**-**	**-**	**-**	**-**
**CBC 16 A - E**	**-**	**-**	**-**	**-**	**-**
**CBC 17 A - X**	**-**	**-**	**-**	**-**	**-**
**CBC 17 A – B**	**-**	**-**	**-**	**-**	**-**
**CBC 17 A – C**	**-**	**-**	**-**	**-**	**-**
**CBC 17 A – D**	**-**	**-**	**-**	**-**	**-**
**CBC 17 A - E**	**-**	**-**	**-**	**-**	**-**
**CBC 18 A - X**	**-**	**-**	**-**	**-**	**-**
**CBC 18 A – B**	**-**	**-**	**-**	**-**	**-**
**CBC 18 A – C**	**-**	**-**	**-**	**-**	**-**
**CBC 18 A – D**	**-**	**-**	**-**	**-**	**-**
**CBC 18 A - E**	**-**	**-**	**-**	**-**	**-**
**CBC 19 A - X**	**-**	**-**	**-**	**-**	**-**
**CBC 19 A – B**	**-**	**-**	**-**	**-**	**-**
**CBC 19 A – C**	**-**	**-**	**-**	**-**	**-**
**CBC 19 A – D**	**-**	**-**	**-**	**-**	**-**
**CBC 19 A - E**	**-**	**-**	**-**	**-**	**-**

**CBC 20 A - X**	**-**	**-**	**-**	**-**	**-**
**CBC 20 A – B**	**-**	**-**	**-**	**-**	**-**
**CBC 20 A – C**	**-**	**-**	**-**	**-**	**-**
**CBC 20 A – D**	**-**	**-**	**-**	**-**	**-**
**CBC 20 A - E**	**-**	**-**	**-**	**-**	**-**
**CBC 21 A - X**	**-**	**-**	**-**	**-**	**-**
**CBC 21 A – B**	**-**	**-**	**-**	**-**	**-**
**CBC 21 A – C**	**-**	**-**	**A**	**-**	**-**
**CBC 21 A – D**	**-**	**-**	**A**	**-**	**-**
**CBC 21 A - E**	**-**	**-**	**-**	**-**	**-**
**CBC 22 A - X**	**-**	**-**	**-**	**-**	**-**
**CBC 22 A – B**	**-**	**-**	**-**	**-**	**-**
**CBC 22 A – C**	**-**	**-**	**-**	**-**	**-**
**CBC 22 A – D**	**-**	**-**	**-**	**-**	**-**
**CBC 22 A - E**	**-**	**-**	**-**	**-**	**-**
**CBC 23 A - X**	**-**	**-**	**-**	**-**	**-**
**CBC 23 A – B**	**-**	**-**	**-**	**-**	**-**
**CBC 23 A – C**	**-**	**-**	**-**	**-**	**-**
**CBC 23 A – D**	**-**	**-**	**-**	**-**	**-**
**CBC 23 A - E**	**-**	**-**	**-**	**-**	**-**
**CBC 24 A - X**	**-**	**-**	**-**	**-**	**-**
**CBC 24 A – B**	**-**	**-**	**-**	**-**	**-**
**CBC 24 A – C**	**-**	**-**	**-**	**-**	**-**
**CBC 24 A – D**	**-**	**-**	**-**	**-**	**-**
**CBC 24 A - E**	**-**	**-**	**-**	**-**	**-**
**CBC 25 A - X**	**-**	**-**	**-**	**-**	**-**

**CBC 25 A – B**	-	-	-	-	-
**CBC 25 A – C**	-	-	-	-	-
**CBC 25 A – D**	-	-	-	-	-
**CBC 25 A - E**	-	-	-	-	-
**CBC 26 A - X**	-	-	-	-	-
**CBC 26 A – B**	-	-	-	-	-
**CBC 26 A – C**	-	-	**A**	-	-
**CBC 26 A – D**	-	-	-	-	-
**CBC 26 A - E**	-	-	-	-	-
**CBC 27 A - X**	-	-	-	-	-
**CBC 27 A – B**	-	-	-	-	-
**CBC 27 A – C**	-	-	-	-	-
**CBC 27 A – D**	-	-	-	-	-
**CBC 27 A - E**	-	-	-	-	-
**CBC 28 A - X**	-	-	-	-	-
**CBC 28 A – B**	-	-	-	-	-
**CBC 28 A – C**	-	-	-	-	-
**CBC 28 A – D**	-	-	-	-	-
**CBC 28 A - E**	-	-	-	-	-
**CBC 29 A - X**	-	-	-	-	-
**CBC 29 A – B**	-	-	-	-	-
**CBC 29 A – C**	-	-	-	-	-
**CBC 29 A – D**	-	-	-	-	-
**CBC 29 A - E**	-	-	-	-	-
**CBC 30 A - X**	-	-	-	-	-
**CBC 30 A – B**	-	-	-	-	-
**CBC 30 A – C**	-	-	-	-	-

**CBC 30 A – D**	**-**	**-**	**-**	**-**	**-**
**CBC 30 A - E**	**-**	**-**	**-**	**-**	**-**
**CBC 31 A - X**	**-**	**-**	**-**	**-**	**-**
**CBC 31 A – B**	**-**	**-**	**-**	**-**	**-**
**CBC 31 A – C**	**-**	**-**	**-**	**-**	**-**
**CBC 31 A – D**	**-**	**-**	**-**	**-**	**-**
**CBC 31 A - E**	**-**	**-**	**-**	**-**	**-**
**CBC 32 A - X**	**-**	**-**	**-**	**-**	**-**
**CBC 32 A – B**	**-**	**-**	**-**	**-**	**-**
**CBC 32 A – C**	**-**	**-**	**-**	**-**	**-**
**CBC 32 A – D**	**-**	**-**	**WA**	**-**	**-**
**CBC 32 A - E**	**-**	**-**	**-**	**-**	**-**
**CBC 33 A - X**	**-**	**-**	**-**	**-**	**-**
**CBC 33 A – B**	**-**	**-**	**-**	**-**	**-**
**CBC 33 A – C**	**-**	**-**	**-**	**-**	**-**
**CBC 33 A – D**	**-**	**-**	**-**	**-**	**-**
**CBC 33 A - E**	**-**	**-**	**-**	**-**	**-**
**CBC 34 A - X**	**-**	**-**	**-**	**-**	**-**
**CBC 34 A – B**	**-**	**-**	**-**	**-**	**-**
**CBC 34 A – C**	**-**	**-**	**-**	**-**	**-**
**CBC 34 A – D**	**-**	**-**	**-**	**-**	**-**
**CBC 34 A - E**	**-**	**-**	**-**	**-**	**-**
**CBC 35 A - X**	**-**	**-**	**-**	**-**	**-**
**CBC 35 A – B**	**-**	**-**	**-**	**-**	**-**
**CBC 35 A – C**	**-**	**-**	**-**	**-**	**-**
**CBC 35 A – D**	**-**	**-**	**-**	**-**	**-**
**CBC 35 A - E**	**-**	**-**	**-**	**-**	**-**

**CBC 36 A - X**	**-**	**-**	**-**	**-**	**-**
**CBC 36 A – B**	**-**	**-**	**-**	**-**	**-**
**CBC 36 A – C**	**-**	**-**	**-**	**-**	**-**
**CBC 36 A – D**	**-**	**-**	**-**	**-**	**-**
**CBC 36 A - E**	**-**	**-**	**-**	**-**	**-**
**CBC 37 A - X**	**-**	**-**	**-**	**-**	**-**
**CBC 37 A – B**	**-**	**-**	**-**	**-**	**-**
**CBC 37 A – C**	**-**	**-**	**-**	**-**	**-**
**CBC 37 A – D**	**-**	**-**	**-**	**-**	**-**
**CBC 37 A - E**	**-**	**-**	**-**	**-**	**-**
**CBC 38 A - X**	**-**	**-**	**-**	**-**	**-**
**CBC 38 A – B**	**-**	**-**	**-**	**-**	**-**
**CBC 38 A – C**	**-**	**-**	**WA**	**-**	**-**
**CBC 38 A – D**	**-**	**-**	**A**	**-**	**-**
**CBC 38 A - E**	**-**	**-**	**-**	**-**	**-**
**CBC 39 A - X**	**-**	**-**	**-**	**-**	**-**
**CBC 39 A – B**	**-**	**-**	**-**	**-**	**-**
**CBC 39 A – C**	**-**	**-**	**-**	**-**	**-**
**CBC 39 A – D**	**-**	**-**	**-**	**-**	**-**
**CBC 39 A - E**	**-**	**-**	**-**	**-**	**-**
**CBC 40 A - X**	**-**	**-**	**-**	**-**	**-**
**CBC 40 A – B**	**-**	**-**	**-**	**-**	**-**
**CBC 40 A – C**	**-**	**-**	**-**	**-**	**-**
**CBC 40 A – D**	**-**	**-**	**-**	**-**	**-**
**CBC 40 A - E**	**-**	**WA**	**A**	**-**	**-**
**CBC 41 A - X**	**-**	**-**	**-**	**-**	**-**

**CBC 41 A – B**	**-**	**-**	**-**	**-**	**-**
**CBC 41 A – C**	**-**	**-**	**WA**	**-**	**-**
**CBC 41 A – D**	**-**	**-**	**-**	**-**	**-**
**CBC 41 A - E**	**-**	**-**	**-**	**-**	**-**
**CBC 42 A - X**	**-**	**-**	**-**	**-**	**-**
**CBC 42 A – B**	**-**	**-**	**-**	**-**	**-**
**CBC 42 A – C**	**-**	**-**	**WA**	**-**	**-**
**CBC 42 A – D**	**-**	**-**	**-**	**-**	**-**
**CBC 42 A - E**	**-**	**-**	**-**	**-**	**-**
**CBC 43 A - X**	**-**	**-**	**-**	**-**	**-**
**CBC 43 A – B**	**-**	**-**	**-**	**-**	**-**
**CBC 43 A – C**	**-**	**-**	**-**	**-**	**-**
**CBC 43 A – D**	**-**	**-**	**-**	**-**	**-**
**CBC 43 A - E**	**-**	**-**	**-**	**-**	**-**
**CBC 44 A - X**	**-**	**-**	**-**	**-**	**-**
**CBC 44 A – B**	**-**	**-**	**-**	**-**	**-**
**CBC 44 A – C**	**-**	**-**	**-**	**-**	**-**
**CBC 44 A – D**	**-**	**-**	**A**	**-**	**-**
**CBC 44 A - E**	**-**	**-**	**-**	**-**	**-**
**CBC 45 A - X**	**A**	**WA**	**A**	**-**	**-**
**CBC 45 A – B**	**A**	**-**	**-**	**-**	**-**
**CBC 45 A – C**	**A**	**A**	**A**	**-**	**-**
**CBC 45 A – D**	**A**	**A**	**A**	**-**	**-**
**CBC 45 A - E**	**A**	**WA**	**A**	**-**	**-**
**CBC 46 A - X**	**-**	**-**	**-**	**-**	**-**
**CBC 46 A – B**	**-**	**-**	**-**	**-**	**-**
**CBC 46 A – C**	**-**	**-**	**-**	**-**	**-**

CBC 46 A – D	-	-	-	-	-
CBC 46 A - E	-	-	-	-	-
CBC 46 C - X	-	-	-	-	-
CBC 46 C – B	-	-	-	-	-
CBC 46 C – C	A	A	A	-	-
CBC 46 C – D	WA	-	WA	-	-
CBC 46 C - E	-	-	-	-	-
CBC 47 A - X	-	-	-	-	-
CBC 47 A – B	-	-	-	-	-
CBC 47 A – C	-	-	-	-	-
CBC 47 A – D	-	-	A	-	-
CBC 47 A - E	-	-	-	-	-
CBC 48 A - X	-	-	-	-	-
CBC 48 A – B	-	-	-	-	-
CBC 48 A – C	-	-	-	-	-
CBC 48 A – D	-	-	A	-	-
CBC 48 A - E	-	-	-	-	-
CBC 49 A - X	-	-	-	-	-
No sample B	No sample B	No sample B	No sample B	No sample B	No sample B
CBC 49 A – C	-	-	-	-	-
CBC 49 A – D	-	-		-	-
CBC 49 A - E	-	-	-	-	-
CBC 50 A - X	-	-	-	-	-
CBC 50 A – B	-	-	-	-	-
CBC 50 A – C	-	-	-	-	-
CBC 50 A – D	-	-	A	-	-
CBC 50 A - E	-	-	-	-	-

CBC 51 A - X	-	-	-	-	-
CBC 51 A – B	-	-	-	-	-
CBC 51 A – C	-	-	A	-	-
CBC 51 A – D	-	-	A	-	-
CBC 51 A - E	-	-	-	-	-
CBC 53 A - X	-	-	-	-	-
CBC 53 A – B	-	-	-	-	-
CBC 53 A – C	-	-	-	-	-
CBC 53 A – D	-	-	-	-	-
CBC 53 A - E	-	-	-	-	-
CBC 55 A - X	-	-	-	-	-
CBC 55 A – B	-	-	-	-	-
CBC 55 A – C	A	-	-	-	-
CBC 55 A – D	-	-	-	-	-
CBC 55 A - E	-	-	-	-	-
CBC 74 A (Numb. 1) - X	-	-	-	-	-
CBC 74 A (Numb. 1) – B	-	-	-	-	-
CBC 74 A (Numb. 1) – C	-	-	-	-	-
CBC 74 A (Numb. 1) – D	-	-	A	-	-
CBC 74 A (Numb. 1) - E	-	-	-	-	-
CBC 74 A (Numb. 3) - X	-	-	-	-	-
CBC 74 A (Numb. 3) – B	-	-	-	-	-
CBC 74 A (Numb. 3) – C	-	-	-	-	-
CBC 74 A (Numb. 3) – D	-	-	A	-	-
CBC 74 A (Numb. 3) - E	-	-	-	-	-
CBC 74 B - X	-	-	-	-	-

**CBC 74 B – B**	**-**	**-**	**-**	**-**	**-**
**CBC 74 B – C**	**-**	**-**	**-**	**-**	**-**
**CBC 74 B – D**	**-**	**-**	**A**	**-**	**-**
**CBC 74 B - E**	**-**	**-**	**-**	**-**	**-**
**CBC 75 A - X**	**-**	**-**	**-**	**-**	**-**
**CBC 75 A – B**	**-**	**-**	**-**	**-**	**-**
**CBC 75 A – C**	**-**	**-**	**-**	**-**	**-**
**CBC 75 A – D**	**-**	**-**	**-**	**-**	**-**
**CBC 75 A - E**	**-**	**-**	**-**	**-**	**-**
**CBC 77 A - X**	**-**	**-**	**-**	**-**	**-**
**CBC 77 A – B**	**-**	**-**	**-**	**-**	**-**
**CBC 77 A – C**	**-**	**-**	**-**	**-**	**-**
**CBC 77 A – D**	**-**	**-**	**-**	**-**	**-**
**CBC 77 A - E**	**-**	**-**	**-**	**-**	**-**

CBC 56 A, CBC 57 A and CBC 62 A aren’t available for testing.
