# Supplementary material for: Probing the Therapeutic Potential of Marine Phyla by SPE Extraction
Source: Mar Drugs. 2021 Nov 16;19(11):640. doi: 10.3390/md19110640 (PMC8625500; doi:10.3390/md19110640)
Supplement: Supplementary file 1 [file marinedrugs-19-00640-s001.zip › File S3 (antidiabetic data).pdf]

PTP-1B and the T–Cell-PTP counter screen assay of the in-house library of Marine Natural Products: the enzymatic activity was evaluated after treatment with 50 µg/mL of either extracts (X) or its related SPE fractions (B-E). Inhibition was calculated by comparing the measurements with two controls: the negative control was the untreated enzyme activity, establishing the 100% of the normal activity; for the positive control it was used a known inhibitor and it was considered as the 0% of the enzymatic activity. Lower activities (negative value) were possible with strong and unselective substances. In the table below, results were expressed as percentage of enzyme activity. The threshold for considering active the treatment (A) was a residual enzyme activity below 30% (in brackets the measured value). Active samples in the PTP-1B assay were tested on T-Cell-PTP. Selective inhibitors should be no active (NA) or weakly active (WA) on this latter assay. The most interesting fractions have been highlighted in bold.

**Identification code**	**PTP-1B**	**TC-PTP**
CBC 1 A - X	-	-
CBC 1 A – B	-	-
CBC 1 A – C	-	-
CBC 1 A – D	A (7%)	A (-32%)
CBC 1 A – E	-	-
CBC 2 A - X	-	-
CBC 2 A – B	-	-
CBC 2 A – C	-	-
CBC 2 A – D	-	-
CBC 2 A – E	-	-
CBC 3 A - X	-	-
CBC 3 A – B	-	-
CBC 3 A – C	-	-
CBC 3 A – D	-	-
CBC 3 A - E	-	-
CBC 4 A - X	-	-
CBC 4 A – B	-	-
CBC 4 A – C	A (11%)	A (-30%)
CBC 4 A – D	-	-
CBC 4 A - E	-	-
CBC 10 A - X	-	-
CBC 10 A – B	-	-

CBC 10 A – C	-	-.
CBC 10 A – D	-	-
CBC 10 A - E	-	-
CBC 11 A - X	-	-
CBC 11 A – B	-	-
CBC 11 A – C	-	-
CBC 11 A – D	A (8%)	A (-40)
CBC 11 A - E	-	-
CBC 12 A - X	-	-
CBC 12 A – B	-	-
CBC 12 A – C	-	-
CBC 12 A – D	-	-
CBC 12 A - E	-	-
CBC 13 A - X	-	-
CBC 13 A – B	-	-
CBC 13 A – C	-	-
CBC 13 A – D	A (28%)	A (-16%)
CBC 13 A - E	-	-
CBC 14 A - X	-	-
CBC 14 A – B	-	-
CBC 14 A – C	-	-
CBC 14 A – D	-	-
CBC 14 A - E	-	-
CBC 15 A - X	-	-
CBC 15 A – B	-	-
CBC 15 A – C	-	-
CBC 15 A – D	-	-
CBC 15 A - E	-	-
CBC 16 A - X	-	-
CBC 16 A – B	-	-
CBC 16 A – C	-	-
CBC 16 A – D	A (9%)	A (-31%)
CBC 16 A - E	-	-
CBC 17 A - X	-	-
CBC 17 A – B	-	-
CBC 17 A – C	-	-
CBC 17 A – D	A (19%)	A (12%)
CBC 17 A - E	-	-
CBC 18 A - X	-	-
CBC 18 A – B	-	-
CBC 18 A – C	-	-
CBC 18 A – D	-	-

CBC 18 A - E	-	-
CBC 19 A - X	-	-
CBC 19 A – B	-	-
CBC 19 A – C	-	-
CBC 19 A – D	-	-
CBC 19 A - E	-	-
CBC 20 A - X	-	-
CBC 20 A – B	-	-
CBC 20 A – C	-	-
CBC 20 A – D	-	-
CBC 20 A - E	-	-
CBC 21 A - X	-	-
CBC 21 A – B	-	-
CBC 21 A – C	-	-
CBC 21 A – D	A (6%)	A (-49%)
CBC 21 A - E	-	-
CBC 22 A - X	-	-
CBC 22 A – B	-	-
CBC 22 A – C	-	-
CBC 22 A – D	-	-
CBC 22 A - E	-	-
CBC 23 A - X	-	-
CBC 23 A – B	-	-
CBC 23 A – C	-	-
CBC 23 A – D	-	-
CBC 23 A - E	-	-
CBC 24 A - X	-	-
CBC 24 A – B	-	-
CBC 24 A – C	-	-
CBC 24 A – D	-	-
CBC 24 A - E	-	-
CBC 25 A - X	-	-
CBC 25 A – B	-	-
CBC 25 A – C	A (21%)	A (7%)
CBC 25 A – D	-	-
CBC 25 A - E	-	-
CBC 26 A - X	-	-
CBC 26 A – B	-	-
CBC 26 A – C	A (15%)	A (-30%)
CBC 26 A – D	-	-
CBC 26 A - E	-	-

CBC 27 A - X	-	-
CBC 27 A – B	-	-
CBC 27 A – C	A (18%)	A (-13%)
CBC 27 A – D	-	-
CBC 27 A - E	-	-
CBC 28 A - X	-	-
CBC 28 A – B	-	-
CBC 28 A – C	-	-
CBC 28 A – D	-	-
CBC 28 A - E	-	-
CBC 29 A - X	-	-
CBC 29 A – B	-	-
CBC 29 A – C	-	-
CBC 29 A – D	-	-
CBC 29 A - E	-	-
CBC 30 A - X	-	-
CBC 30 A – B	-	-
CBC 30 A – C	-	-
CBC 30 A – D	-	-
CBC 30 A - E	-	-
CBC 31 A - X	-	-
CBC 31 A – B	-	-
CBC 31 A – C	-	-
CBC 31 A – D	-	-
CBC 31 A - E	-	-
CBC 32 A - X	-	-
CBC 32 A – B	-	-
CBC 32 A – C	-	-
CBC 32 A – D	A (4%)	A (1%)
CBC 32 A - E	-	-
CBC 33 A - X	-	-
CBC 33 A – B	-	-
CBC 33 A – C	-	-
CBC 33 A – D	-	-
CBC 33 A - E	-	-
CBC 34 A - X	-	-
CBC 34 A – B	-	-
CBC 34 A – C	-	-
CBC 34 A – D	-	-
CBC 34 A - E	-	-
CBC 35 A - X	-	-
CBC 35 A – B	-	-

CBC 35 A – C	-	-
CBC 35 A – D	-	-
CBC 35 A - E	-	-
CBC 36 A - X	-	-
CBC 36 A – B	-	-
CBC 36 A – C	-	-
CBC 36 A – D	-	-
CBC 36 A - E	-	-
CBC 37 A - X	-	-
CBC 37 A – B	-	-
CBC 37 A – C	-	-
CBC 37 A – D	-	-
CBC 37 A - E	-	-
CBC 38 A - X	-	-
CBC 38 A – B	-	-
CBC 38 A – C	-	-
CBC 38 A – D	A (2%)	A (-57%)
CBC 38 A - E	-	-
CBC 39 A - X	-	-
CBC 39 A – B	-	-
CBC 39 A – C	A (-2%)	A (-29%)
CBC 39 A – D	-	-
CBC 39 A - E	-	-
CBC 40 A - X	-	-
CBC 40 A – B	-	-
CBC 40 A – C	-	-
CBC 40 A – D	-	-
CBC 40 A - E	A (1%)	A (37%)
CBC 41 A - X	-	-
CBC 41 A – B	-	-
CBC 41 A – C	-	-
CBC 41 A – D	-	-
CBC 41 A - E	-	-
CBC 42 A - X	-	-
CBC 42 A – B	-	-
CBC 42 A – C	A (-3%)	A (-51%)
CBC 42 A – D	-	-
CBC 42 A - E	-	-
CBC 43 A - X	-	-
CBC 43 A – B	-	-
CBC 43 A – C	-	-
CBC 43 A – D	-	-

CBC 43 A - E	-	-
CBC 44 A - X	-	-
CBC 44 A – B	-	-
CBC 44 A – C	-	-
CBC 44 A – D	A (-5%)	A (-25%)
CBC 44 A - E	-	-
CBC 45 A - X	-	-
CBC 45 A – B	A (28%)	A (-18%)
CBC 45 A – C	A (13%)	A (-25%)
CBC 45 A – D	A (-6%)	A (-25%)
CBC 45 A - E	-	-
CBC 46 A - X	-	-
CBC 46 A – B	-	-
CBC 46 A – C	-	-
CBC 46 A – D	-	-
CBC 46 A - E	-	-
CBC 46 C - X	-	-
CBC 46 C – B	-	-
**CBC 46 C – C**	**A (-1%)**	**WA (78%)**
CBC 46 C – D	-	-
CBC 46 C - E	-	-
CBC 47 A - X	-	-
CBC 47 A – B	-	-
CBC 47 A – C	A (-3%)	A (-22%)
CBC 47 A – D	-	-
CBC 47 A - E	-	-
CBC 48 A - X	-	-
CBC 48 A – B	-	-
CBC 48 A – C	-	-
CBC 48 A – D	-	-
CBC 48 A - E	-	-
CBC 49 A - X	-	-
No sample B	No sample B	No sample B
CBC 49 A – C	-	-
CBC 49 A – D	-	-
CBC 49 A - E	-	-
CBC 50 A - X	-	-
CBC 50 A – B	-	-
CBC 50 A – C	-	-
**CBC 50 A – D**	**A (-6%)**	**WA (59 %)**
CBC 50 A - E	-	-

CBC 51 A - X	-	-
CBC 51 A – B	-	-
CBC 51 A – C	-	-
**CBC 51 A – D**	**A (-4%)**	**WA (74%)**
CBC 51 A - E	-	-
CBC 53 A - X	-	-
CBC 53 A – B	-	-
CBC 53 A – C	-	-
CBC 53 A – D	-	-
CBC 53 A - E	-	-
CBC 55 A - X	-	-
CBC 55 A – B	-	-
**CBC 55 A – C**	**A (21%)**	**WA (41%)**
CBC 55 A – D	-	-
CBC 55 A - E	-	-
CBC 74 A (Numb. 1) - X	-	-
CBC 74 A (Numb. 1) – B	-	-
CBC 74 A (Numb. 1) – C	-	-
CBC 74 A (Numb. 1) – D	-	-
CBC 74 A (Numb. 1) - E	-	-
CBC 74 A (Numb. 3) - X	-	-
CBC 74 A (Numb. 3) – B	-	-
CBC 74 A (Numb. 3) – C	-	-
CBC 74 A (Numb. 3) – D	-	-
CBC 74 A (Numb. 3) - E	-	-
CBC 74 B - X	-	-
CBC 74 B – B	-	-
CBC 74 B – C	-	-
CBC 74 B – D	-	-
CBC 74 B - E	-	-
CBC 75 A - X	-	-
CBC 75 A – B	-	-
CBC 75 A – C	-	-
CBC 75 A – D	-	-
CBC 75 A - E	-	-
CBC 77 A - X	-	-
CBC 77 A – B	-	-
CBC 77 A – C	-	-
CBC 77 A – D	A (27%)	A (-44%)
CBC 77 A – E	-	-

CBC56A, CBC57A and CBC62A aren’t available for testing.
