# Supplementary material for: Probing the Therapeutic Potential of Marine Phyla by SPE Extraction
Source: Mar Drugs. 2021 Nov 16;19(11):640. doi: 10.3390/md19110640 (PMC8625500; doi:10.3390/md19110640)
Supplement: Supplementary file 1 [file marinedrugs-19-00640-s001.zip › Supplementary Figures.pdf]

Figure S1. Non-parametric test and Post-hoc analysis: for each cell line (A2780, A549, PNT2) and dose (100, 10, 1 µg/mL) we compute Kruskal-Wallis test (non parametric test). • As the p-value is less than the significance level 0.05, we can conclude that there are significant differences between the Phylum. • A multiple pairwise-comparison between Phylum is then performed to calculate pairwise comparisons between Phylum levels with corrections for multiple testing (Dunnett's Test). • We show only the comparisons vs CTRL

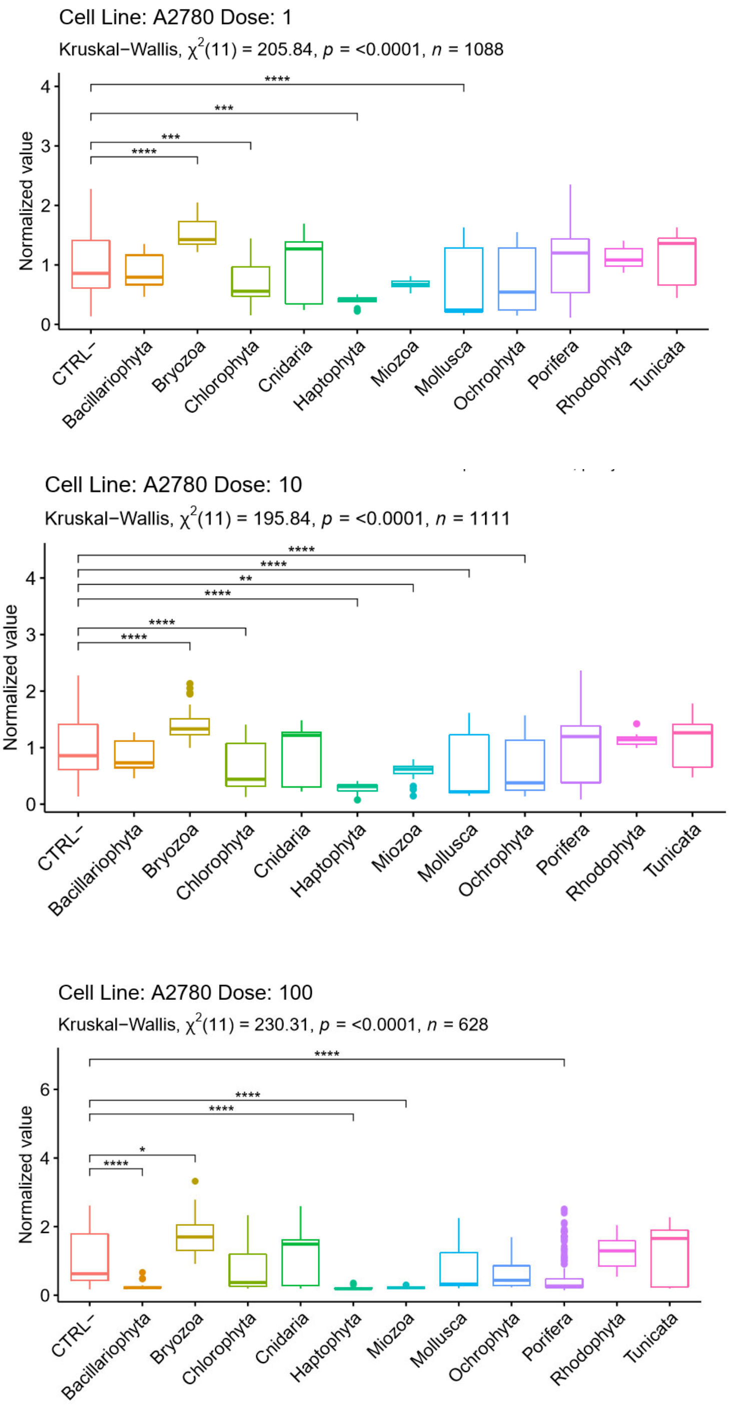

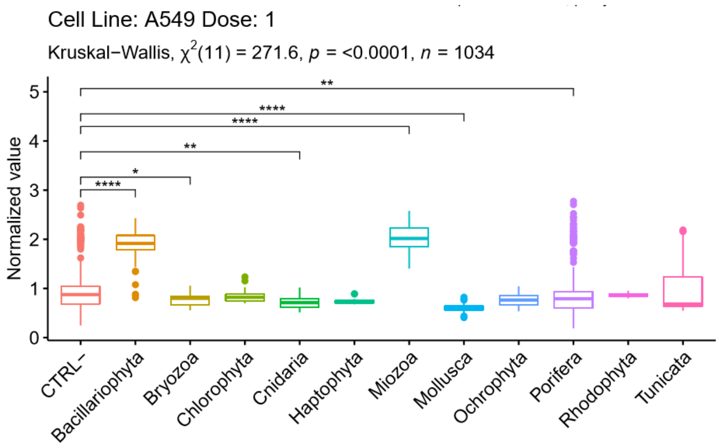
Cell Line: A549 Dose: 1
Kruskal−Wallis, χ2(11) = 271.6, p = <0.0001, n = 1034
Normalized value
0
1
2
3
4
5
CTRL−
Bacillariophyta
Bryozoa
Chlorophyta
Cnidaria
Haptophyta
Miozoa
Mollusca
Ochrophyta
Porifera
Rhodophyta
Tunicata

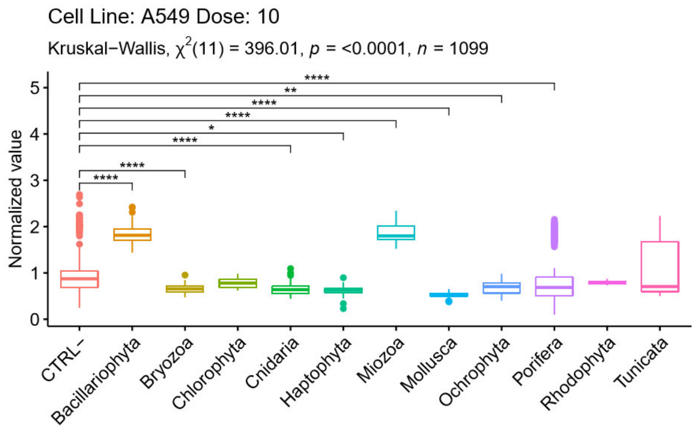
Cell Line: A549 Dose: 10
Kruskal−Wallis, χ2(11) = 396.01, p = <0.0001, n = 1099
Normalized value
0
1
2
3
4
5
CTRL−
Bacillariophyta
Bryozoa
Chlorophyta
Cnidaria
Haptophyta
Miozoa
Mollusca
Ochrophyta
Porifera
Rhodophyta
Tunicata

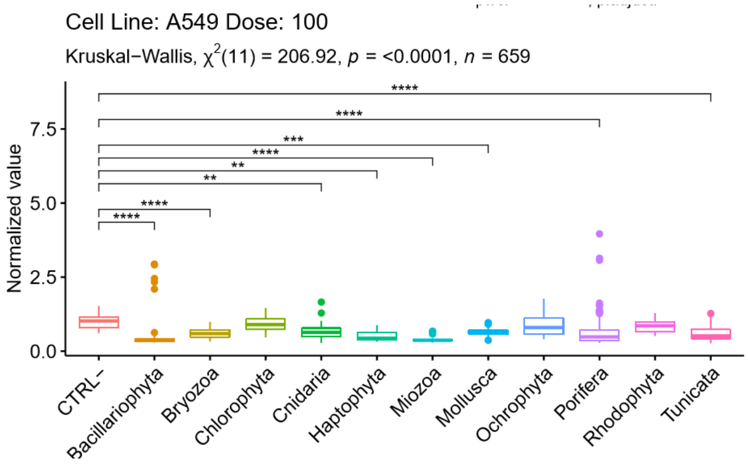
Cell Line: A549 Dose: 100
Kruskal−Wallis, χ2(11) = 206.92, p = <0.0001, n = 659
Normalized value
0.0
2.5
5.0
7.5
CTRL−
Bacillariophyta
Bryozoa
Chlorophyta
Cnidaria
Haptophyta
Miozoa
Mollusca
Ochrophyta
Porifera
Rhodophyta
Tunicata

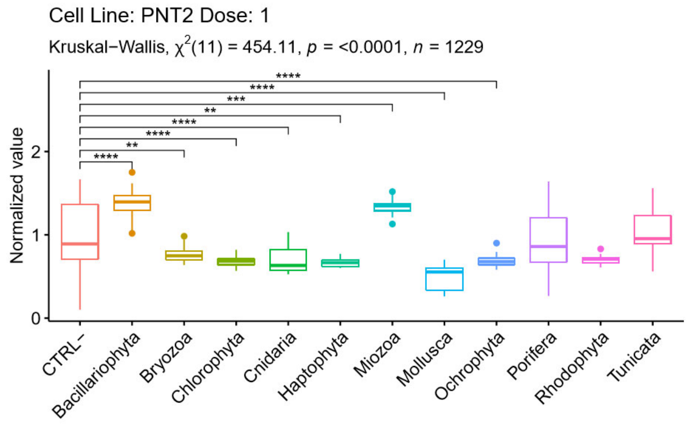
Cell Line: PNT2 Dose: 1
Kruskal−Wallis, χ2(11) = 454.11, p = <0.0001, n = 1229
Normalized value
0
1
2
CTRL−
Bacillariophyta
Bryozoa
Chlorophyta
Cnidaria
Haptophyta
Miozoa
Mollusca
Ochrophyta
Porifera
Rhodophyta
Tunicata

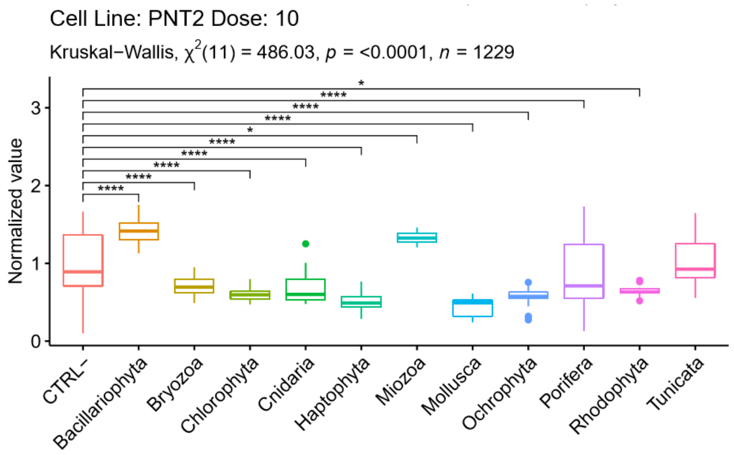
Cell Line: PNT2 Dose: 10
Kruskal−Wallis, χ2(11) = 486.03, p = <0.0001, n = 1229
Normalized value
0
1
2
3
CTRL−
Bacillariophyta
Bryozoa
Chlorophyta
Cnidaria
Haptophyta
Miozoa
Mollusca
Ochrophyta
Porifera
Rhodophyta
Tunicata

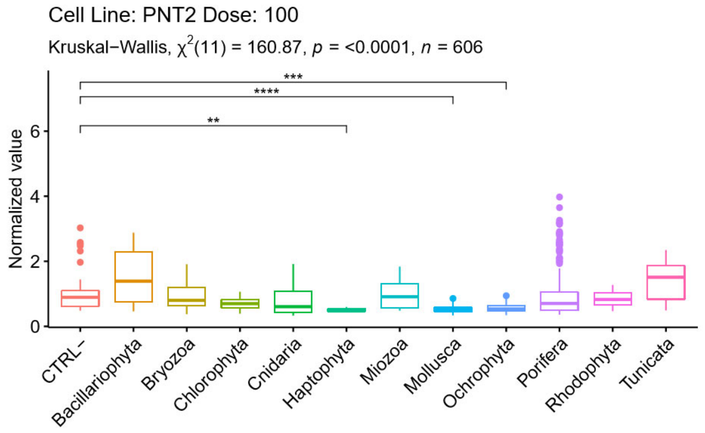
Cell Line: PNT2 Dose: 100
Kruskal−Wallis, χ2(11) = 160.87, p = <0.0001, n = 606
Normalized value
0
2
4
6
CTRL−
Bacillariophyta
Bryozoa
Chlorophyta
Cnidaria
Haptophyta
Miozoa
Mollusca
Ochrophyta
Porifera
Rhodophyta
Tunicata

Figure S2. Barplot: Log Fold-Change for significant comparisons (vs CTRL-)

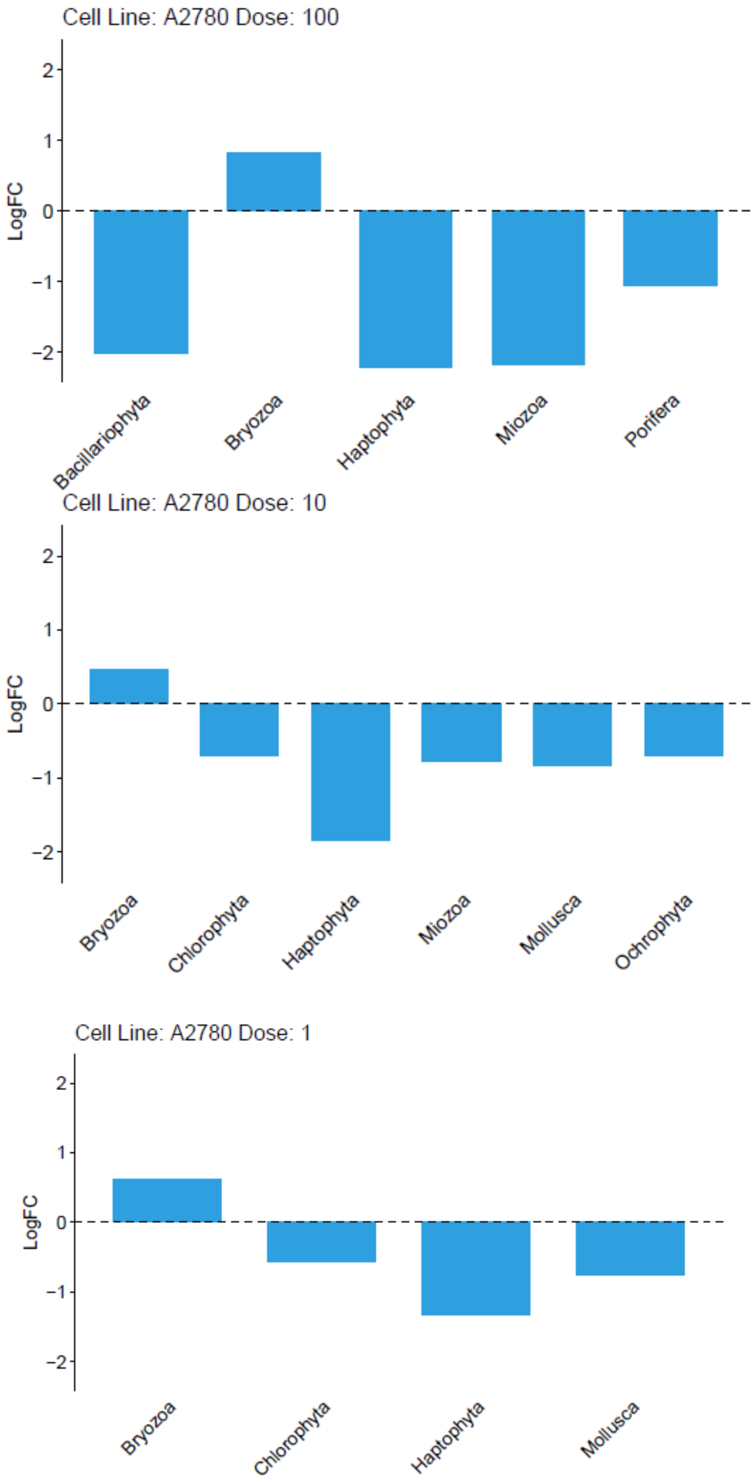

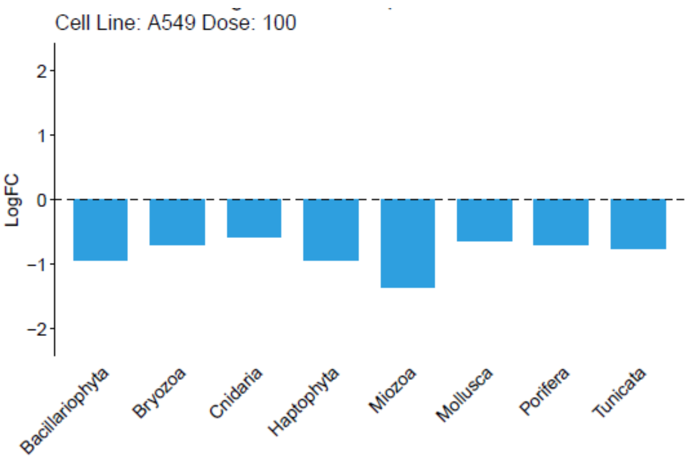
Cell Line: A549 Dose: 100
LogFC
2
1
0
−1
−2
Bacillariophyta
Bryozoa
Cnidaria
Haptophyta
Miozoa
Mollusca
Porifera
Tunicata

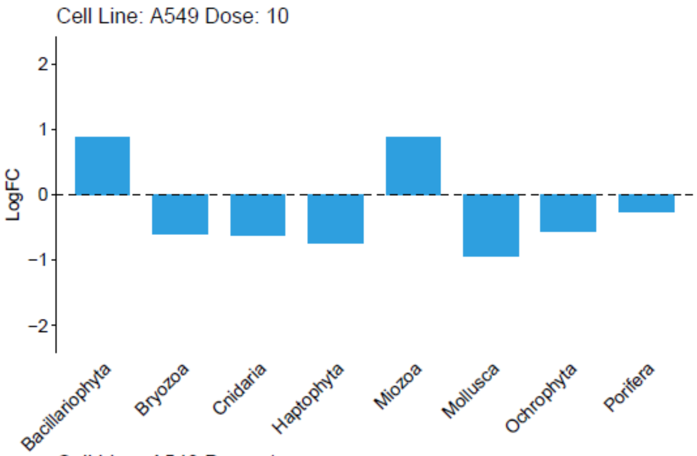
Cell Line: A549 Dose: 10
LogFC
2
1
0
−1
−2
Bacillariophyta
Bryozoa
Cnidaria
Haptophyta
Miozoa
Mollusca
Ochrophyta
Porifera

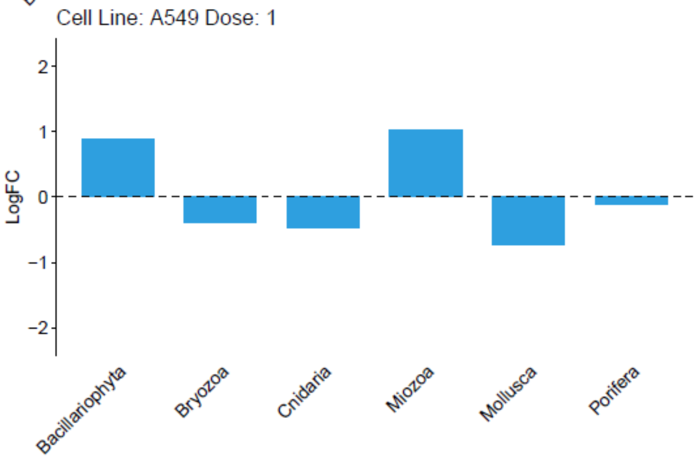
Cell Line: A549 Dose: 1
LogFC
2
1
0
−1
−2
Bacillariophyta
Bryozoa
Cnidaria
Miozoa
Mollusca
Porifera

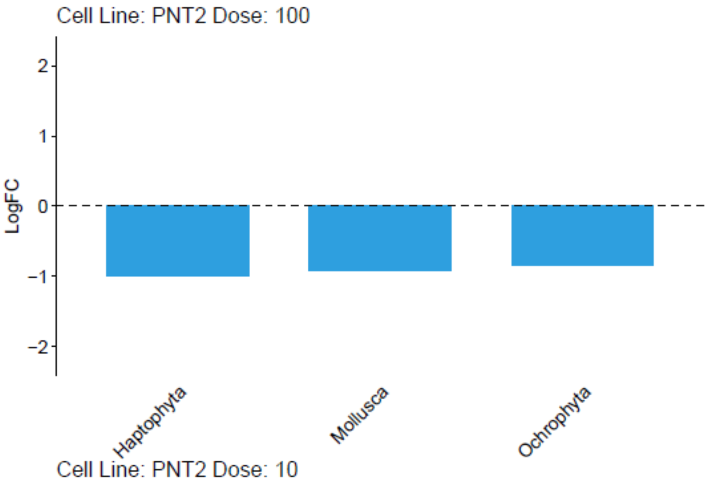
Cell Line: PNT2 Dose: 100
LogFC
2
1
0
−1
−2
Haptophyta
Mollusca
Ochrophyta

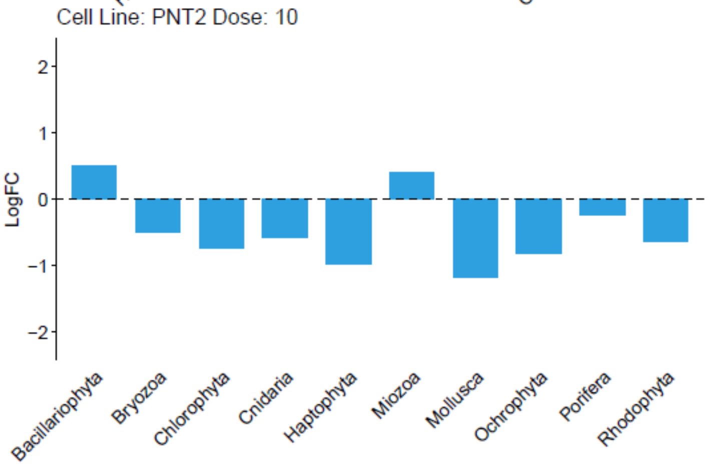
Cell Line: PNT2 Dose: 10
LogFC
2
1
0
−1
−2
Bacillariophyta
Bryozoa
Chlorophyta
Cnidaria
Haptophyta
Miozoa
Mollusca
Ochrophyta
Porifera
Rhodophyta

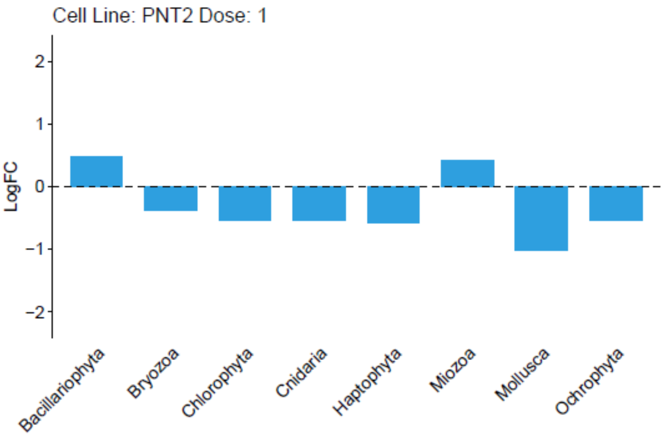
Cell Line: PNT2 Dose: 1
LogFC
2
1
0
−1
−2
Bacillariophyta
Bryozoa
Chlorophyta
Cnidaria
Haptophyta
Miozoa
Mollusca
Ochrophyta

Figure S3. Heatmap showing the percentage of vitality on the three cell lines (A549, A2780; PTN2) after treatment with the raw extracts (X) and the corresponding four SPE-fractions (B, C, D, E) at 1 and 10 µg/ml.

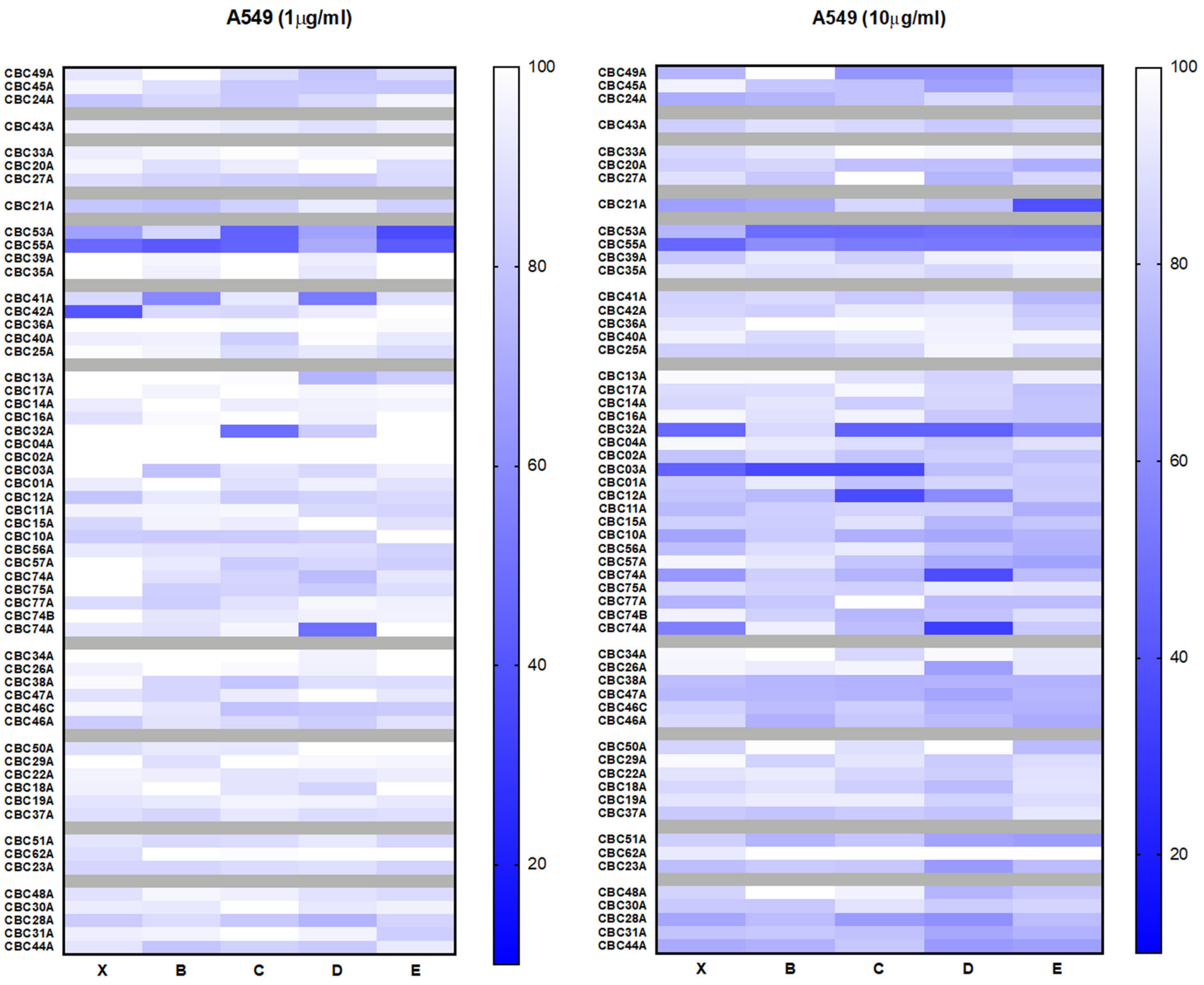

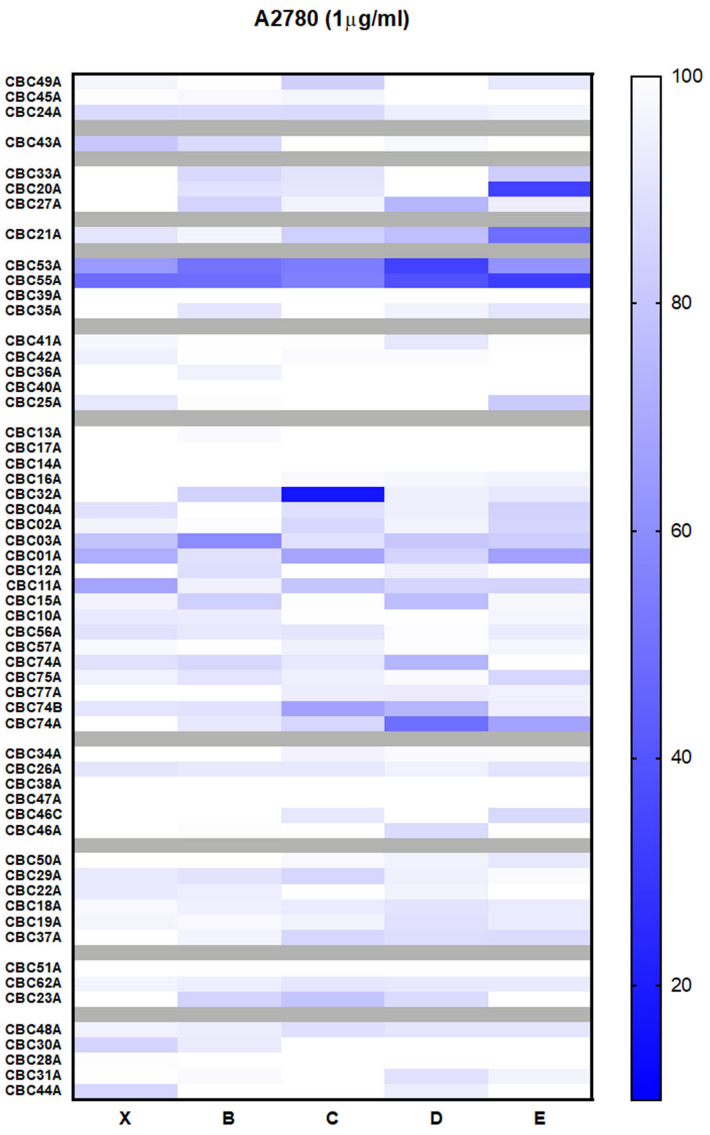
A2780 (1µg/ml)
CBC49A
CBC45A
CBC24A
CBC43A
CBC33A
CBC20A
CBC27A
CBC21A
CBC53A
CBC55A
CBC39A
CBC35A
CBC41A
CBC42A
CBC36A
CBC40A
CBC25A
CBC13A
CBC17A
CBC14A
CBC16A
CBC32A
CBC04A
CBC02A
CBC03A
CBC01A
CBC12A
CBC11A
CBC15A
CBC10A
CBC56A
CBC57A
CBC74A
CBC75A
CBC77A
CBC74B
CBC74A
CBC34A
CBC26A
CBC38A
CBC47A
CBC46C
CBC46A
CBC50A
CBC29A
CBC22A
CBC18A
CBC19A
CBC37A
CBC51A
CBC62A
CBC23A
CBC48A
CBC30A
CBC28A
CBC31A
CBC44A
X
B
C
D
E
100
80
60
40
20

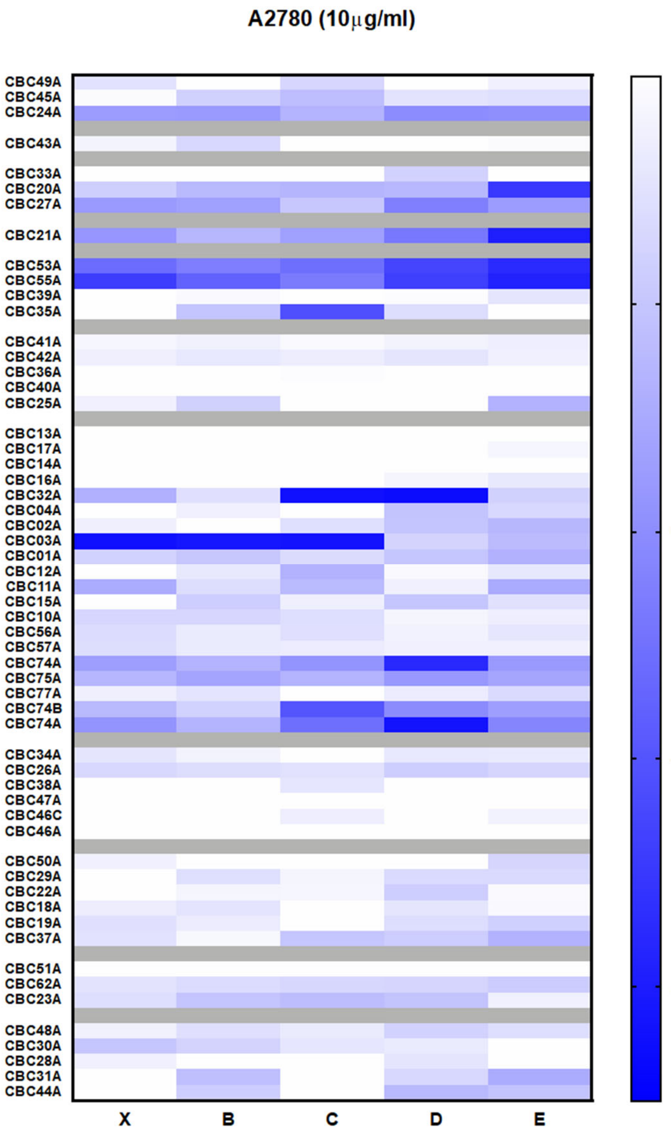
A2780 (10µg/ml)
CBC49A
CBC45A
CBC24A
CBC43A
CBC33A
CBC20A
CBC27A
CBC21A
CBC53A
CBC55A
CBC39A
CBC35A
CBC41A
CBC42A
CBC36A
CBC40A
CBC25A
CBC13A
CBC17A
CBC14A
CBC16A
CBC32A
CBC04A
CBC02A
CBC03A
CBC01A
CBC12A
CBC11A
CBC15A
CBC10A
CBC56A
CBC57A
CBC74A
CBC75A
CBC77A
CBC74B
CBC74A
CBC34A
CBC26A
CBC38A
CBC47A
CBC46C
CBC46A
CBC50A
CBC29A
CBC22A
CBC18A
CBC19A
CBC37A
CBC51A
CBC62A
CBC23A
CBC48A
CBC30A
CBC28A
CBC31A
CBC44A
X
B
C
D
E

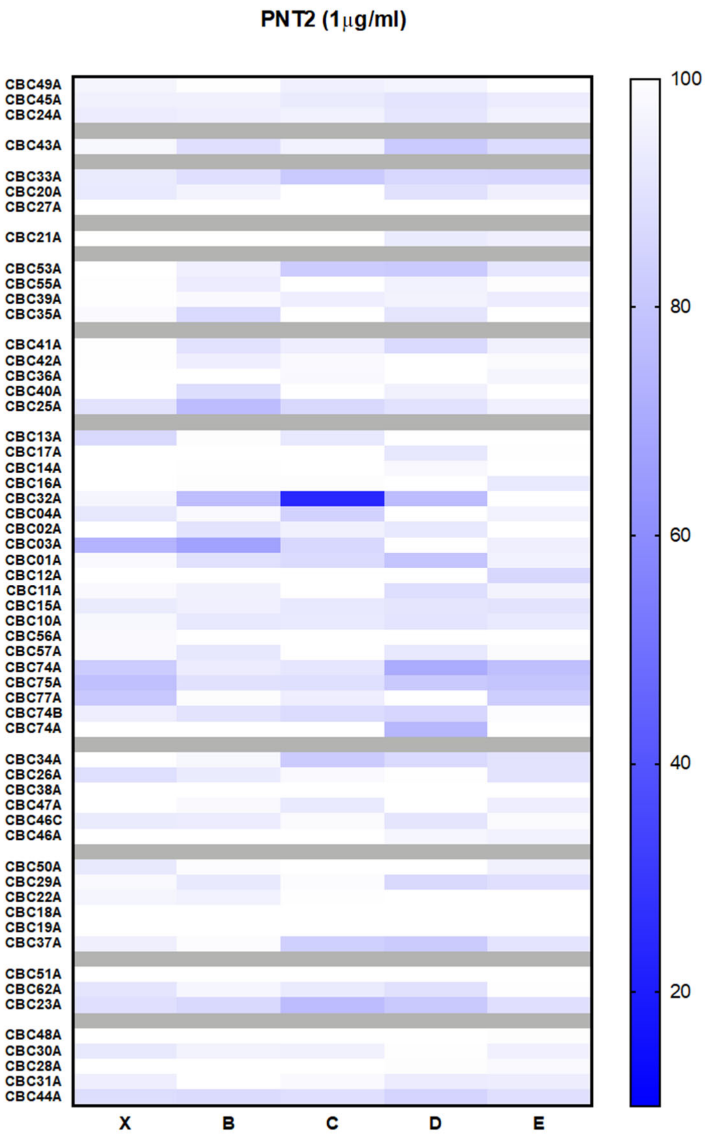
PNT2 (1µg/ml)
CBC49A
CBC45A
CBC24A
CBC43A
CBC33A
CBC20A
CBC27A
CBC21A
CBC53A
CBC55A
CBC39A
CBC35A
CBC41A
CBC42A
CBC36A
CBC40A
CBC25A
CBC13A
CBC17A
CBC14A
CBC16A
CBC32A
CBC04A
CBC02A
CBC03A
CBC01A
CBC12A
CBC11A
CBC15A
CBC10A
CBC56A
CBC57A
CBC74A
CBC75A
CBC77A
CBC74B
CBC74A
CBC34A
CBC26A
CBC38A
CBC47A
CBC46C
CBC46A
CBC50A
CBC29A
CBC22A
CBC18A
CBC19A
CBC37A
CBC51A
CBC62A
CBC23A
CBC48A
CBC30A
CBC28A
CBC31A
CBC44A
X
B
C
D
E
100
80
60
40
20
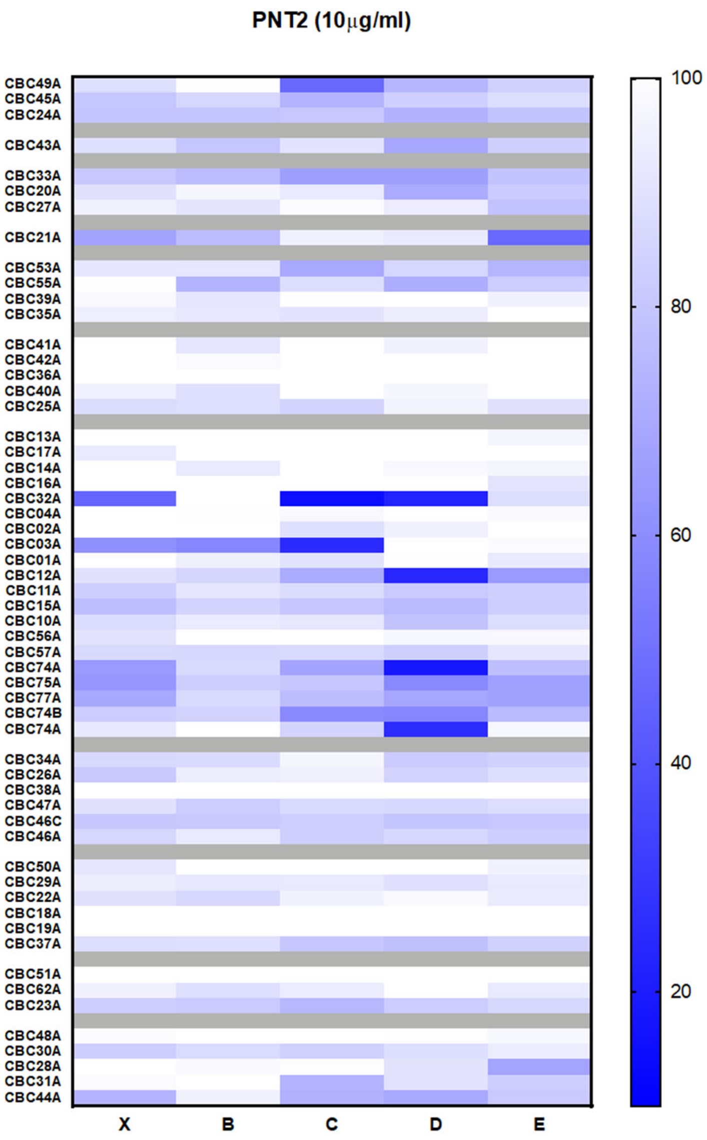
PNT2 (10µg/ml)
CBC49A
CBC45A
CBC24A
CBC43A
CBC33A
CBC20A
CBC27A
CBC21A
CBC53A
CBC55A
CBC39A
CBC35A
CBC41A
CBC42A
CBC36A
CBC40A
CBC25A
CBC13A
CBC17A
CBC14A
CBC16A
CBC32A
CBC04A
CBC02A
CBC03A
CBC01A
CBC12A
CBC11A
CBC15A
CBC10A
CBC56A
CBC57A
CBC74A
CBC75A
CBC77A
CBC74B
CBC74A
CBC34A
CBC26A
CBC38A
CBC47A
CBC46C
CBC46A
CBC50A
CBC29A
CBC22A
CBC18A
CBC19A
CBC37A
CBC51A
CBC62A
CBC23A
CBC48A
CBC30A
CBC28A
CBC31A
CBC44A
X
B
C
D
E
100
80
60
40
20
